# Integrated Fecal Microbiome and Metabolomics Reveals a Novel Potential Biomarker for Predicting Tibial Dyschondroplasia in Chickens

**DOI:** 10.3389/fphys.2022.887207

**Published:** 2022-05-12

**Authors:** Shucheng Huang, Chaodong Zhang, Tingting Xu, Aftab Shaukat, Yanfeng He, Pan Chen, Luxi Lin, Ke Yue, Qinqin Cao, Xishuai Tong

**Affiliations:** ^1^ College of Veterinary Medicine, Henan Agricultural University, Zhengzhou, China; ^2^ National Center for International Research on Animal Genetics, Breeding and Reproduction (NCIRAGBR), Huazhong Agricultural University, Wuhan, China; ^3^ Institutes of Agricultural Science and Technology Development (Joint International Research Laboratory of Agriculture and Agri-Product Safety of the Ministry of Education of China)/College of Veterinary Medicine, Yangzhou University, Yangzhou, China

**Keywords:** tibial dyschondroplasia, gut microbiome, metabolome, diagnosis, biomarker

## Abstract

Tibial dyschondroplasia (TD) is a metabolic tibial-tarsal disorder occurring in fast-growing poultry, and its diagnosis is mainly based on an invasive method. Here, we profiled the fecal gut microbiome and metabolome of broilers with and without TD to identify potential non-invasive and non-stress biomarkers of TD. First, TD broilers with the most pronounced clinical signs during the experiment were screened and faecal samples were collected for integrated microbiome and metabolomics analysis. Moreover, the diagnostic potential of identified biomarkers was further validated throughout the experiment. It was noted that the microbial and metabolic signatures of TD broilers differed from those of normal broilers. TD broilers were characterized by enriched bacterial OTUs of the genus *Klebsiella*, and depleted genera [*Ruminococcus*], *Dorea*, *Ruminococcus*, *Oscillospira*, *Ochrobactrum*, and *Sediminibacterium*. In addition, a total of 189 fecal differential metabolites were identified, mainly enriched in the purine, vitamin and amino acid metabolism, which were closely associated with differential microbiota and tibia-related indicators. Furthermore, three fecal metabolites were screened, including 4-hydroxybenzaldehyde, which distinguished TD from normal broilers with extremely high specificity and was superior to serum bone markers. These results indicated that gut microbiota equilibrium might influence the pathogenesis of TD by modulating host metabolism, and the identified fecal metabolite 4-hydroxybenzaldehyde might be a potential and non-invasive biomarker for predicting TD in chickens.

## Introduction

Tibial dyschondroplasia (TD) is a metabolic cartilage disease that can occur in humans or animals, including poultry, especially broilers and turkeys, accompanied by a rapid growth rate (Poulos, 1978; Leach and Monsonego-Ornan, 2007; Xu et al., 2021). It is characterized by an abnormality in the bone growth plate. This cartilage template is not properly resorbed but replaced by bone, a common cause of deformity and lameness in the broiler chicken (Farquharson and Jefferies, 2000; Rath et al., 2005; Huang et al., 2018). Evidence shows that 12.5 billion birds worldwide have leg problems each year (Almeida Paz et al., 2010). Derakhshanfar *et al.* found that TD affects the skeletal system of 30% of meat chickens and 90% of turkeys (Derakhshanfar et al., 2013). In addition, the abnormalities of growth and development of the skeleton in poultry indirectly lead to reduce gross profits (about 10%–40%) in the poultry industry (Almeida Paz et al., 2010). But, it is challenging to accurately assess the prevalence of TD in broiler management due to its sub-clinical signs and symptoms (Groves and Muir, 2017). However, the formation of broiler TD causes leg weakness, dyskinesias and leg deformities that ultimately prevent standing (Genin et al., 2012; Huang et al., 2019), thus compromising the welfare of the chickens.

Broilers with apparent leg disease can be easily diagnosed based on clinical manifestations, but the condition is usually severe and has no therapeutic value. The most effective method for diagnosing TD in broilers is mainly based on an invasive technique, namely observation of morphological changes in the tibial growth plate (Farquharson and Jefferies, 2000; Rath et al., 2007; Huang et al., 2018). In addition, radiological methods can be used for assessment of broiler TD, but there is a delay in the early diagnosis of sub-clinical TD in broilers and it is complex and time-consuming for the farms (Poulos, 1978; Pelicia et al., 2012). It is well known that early diagnosis is essential for the treatment of metabolic and developmental diseases (Edwards and Veltmann, 1983). Therefore, a non-invasive, low-stress or non-stress method is desired for the darly diagnosis of TD in broiler chickens.

The microbiome that resides in the gastrointestinal tract reflects physiological and metabolic properties. These microorganisms live symbiotically with the host to produce microbial metabolites, forming the host-microbe metabolic axis, which plays a vital role in animal nutritional metabolism and immune homeostasis, including the occurrence and development of disease (Zaiss et al., 2019; Zheng et al., 2019; Kim et al., 2020; Wang et al., 2020; Ling et al., 2021; Murga-Garrido et al., 2021). More evidence has demonstrated that gut microbiota is a critical regulator in bone and that alterations in microbiota composition can contribute to pathological bone loss or reduced bone mineral density. While the changes in microbiota composition can improve calcium absorption and mineral levels by adding nutritional supplements, suggesting that gut microbiota directly and indirectly affects bone metabolism (Di Stefano et al., 2001; Stotzer et al., 2003; Chen et al., 2017; Lu et al., 2021). Furthermore, previous studies have shown significant changes in the structure of the gut microbiota of broiler chickens affected by TD (Tong et al., 2018). Until now, the gut microbial characteristics of broiler TD have not been fully elucidated. Recent studies have highlighted that the detection of characteristics of 16S rRNA gene profiling in gut microbiota provides a plentiful thread for the diagnosis and assessment of diseases, and serves as a predictor or a monitor of disease prognosis of therapeutic intervention (Pan et al., 2020; Hao et al., 2021). This approach may inspire the identification of predictive microbial markers of TD in broilers. Although 16S rRNA gene sequencing technology has been successfully applied in clinical diagnosis, the phylogenetic resolution of the 16S rRNA gene is limited that restricts the universality and significance of these studies (Rodriguez-R et al., 2018; Zhu et al., 2021). The gut metabolome, rather than the gut microbiota itself, may also directly involve TD formation (He et al., 2020; Lu et al., 2021; Xu et al., 2021). Furthermore, fecal metabolomics is derived not only from microbial metabolism that accounts for 15%, but also jointly from diet and host metabolism (HolmesLi et al., 2012; Kim et al., 2020; Murga-Garrido et al., 2021), which provides a complementary functional readout of microbial activity, and combining these two omics-approaches is a mature strategy for identifying potentially disease-related gut microbes and their functional metabolites (Klein et al., 2016; Stewart et al., 2017).

To address these challenges, we analyzed the microbial compositions and metabolic changes of fecal samples in the thiram-induced TD chicken model by combined 16S rRNA gene sequencing and UPLC/MS/MS-based metabolomics. Previous studies have confirmed that the analysis of these two methods is a well-established strategy for uncovering both gut microbial composition and functional features in various diseases (Zheng et al., 2019; Kim et al., 2020; Wang et al., 2020; Chen et al., 2021). Therefore, based on this well-established strategy, we identified the microbial and metabolic signatures of TD in broilers to explore their reciprocal interactions in the gut ecosystem stability of TD. The relationship between altered gut microbes, fecal metabolites and tibia-related indexes (e.g., tibia weight, tibia length, tibia diameter, and tibia growth plate width) was evaluated. Moreover, based on these multi-omic data, we identified three novel differential fecal metabolite biomarkers that can accurately distinguish TD in broiler chickens.

## Results

### Changes in the Tibiae and Their Growth Plate in Broilers With TD

**FIGURE 1:**
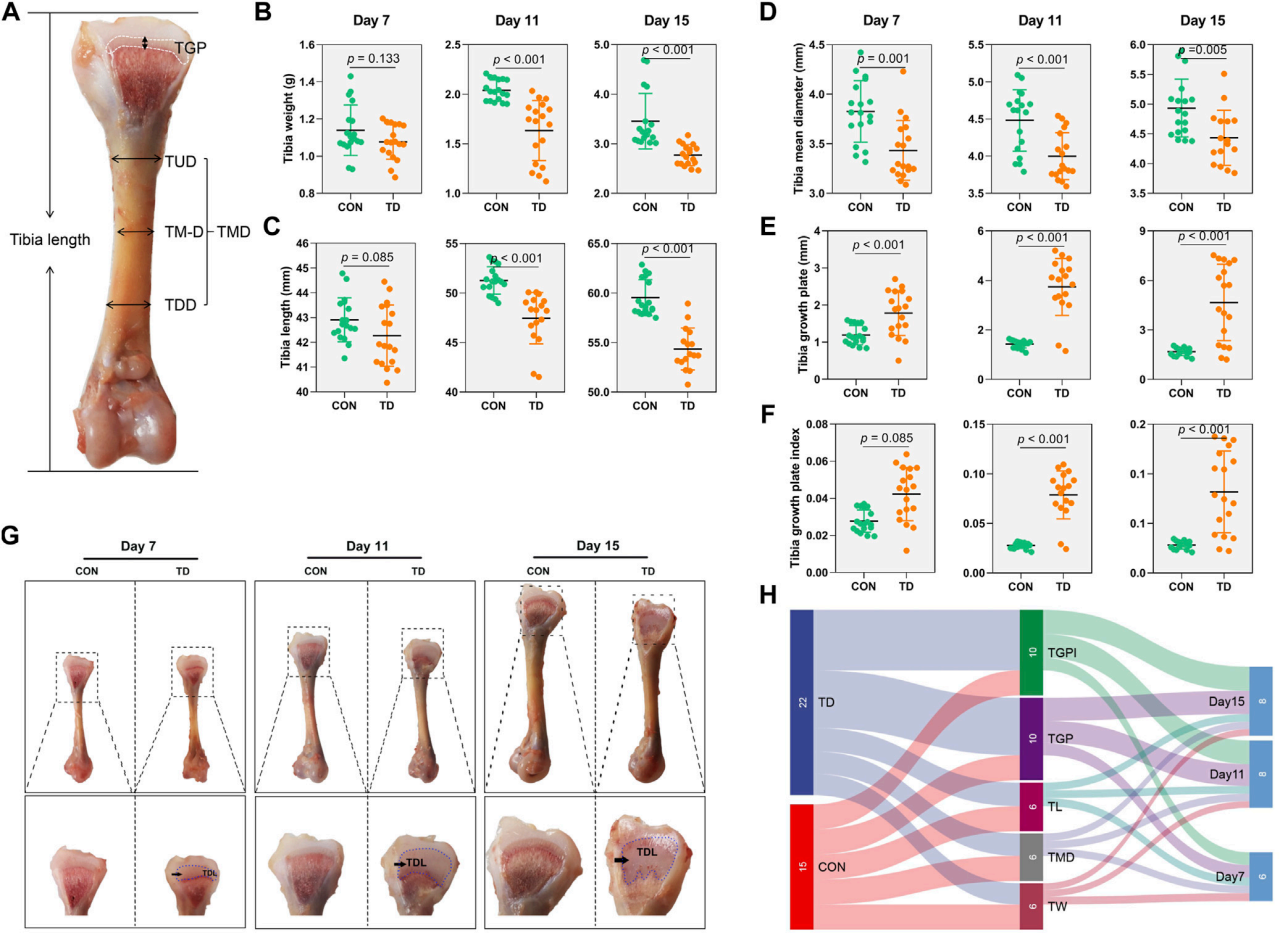
Tibia related-parameters of broiler chickens. **(A)** Standard for the measurement of tibia length, TUD (Upper 1/2 diameter of the tibia), TMD (tibia mid-diameter), and TLD (Lower 1/2 diameter of the tibia). TMD (tibia mean diameter) comes from the average of the TUD, TMD, and TLD. **(B–F)** The tibia weight, tibia length, tibia mean diameter (TMD), tibia growth plate, and tibia growth plate index were recorded and compared between the CON and TD groups from 7-, 11-, and 15-day-old broiler chickens. A statistical difference of p-value less than 0.05; data represent means ± SD, two-tailed unpaired Student’s t-test. N = 18. **(G)** Morphological observation of the tibial growth plate in the CON and TD groups from 7-, 11-, and 15-day-old broiler chickens. The black arrows indicate TD lesion. **(H)** Sankey diagram depicting tibia related-parameters by test point in the CON and TD groups of broiler chickens.

To determine the changes in tibia-related parameters in broilers in the CON and TD groups, tibia weight, tibia length and tibia diameter were measured (Figures 1A–F). The tibia weight and length of broiler chickens in the TD group were markedly (*p* < 0.001 and *p* < 0.001, respectively) decreased compared to the CON group on days 11 and 15. Similarly, the tibia mean diameter of broiler chickens with TD was also significantly (*p* = 0.001, *p* < 0.001, and *p* = 0.005, respectively) decreased during the experiment compared to normal broiler chickens of CON group. Our previous study showed the dynamics of cartilaginous growth plates in broiler chickens play central roles in the proper development and growth of the tibiae (Huang et al., 2018). The morphological observation found that the width of the tibia growth plate (TGP) in the TD group was significantly widened on days 7 and 11 compared to the CON group (Figure 1G). On day 15, the TGP width of the TD group was significantly increased and the area of the TD lesion was increased (Figure 1G). Quantitative histomorphology analysis of the bone showed that the TGP width and its index were markedly higher in the TD group than the CON group on days 7, 11 and 15 (Figures 1E,F). These results collectively demonstrated that tibia-related parameters were obviously altered in TD broilers.

Sankey diagram is a specific type of flow diagram that usually displays the flows and quantities from one set of values to the other proportion, and is used to further determine which experimental stage of TD broiler chickens tibia-related parameters changed most significantly. The results showed that the width of tibia growth plate and its index accounted for a high proportion of tibia-related parameters, mainly derived from the TD group. In addition, the proportion of tibia-related parameters was much more significant on days 10 and 15 than that on day 7 (Figure 1H), indicating that the tibia growth plates of TD broilers showed the most observable changes on days 11 and 15 than on day 7.

### Fecal Microbial Structure of Two Groups in Broiler Chickens

**FIGURE 2:**
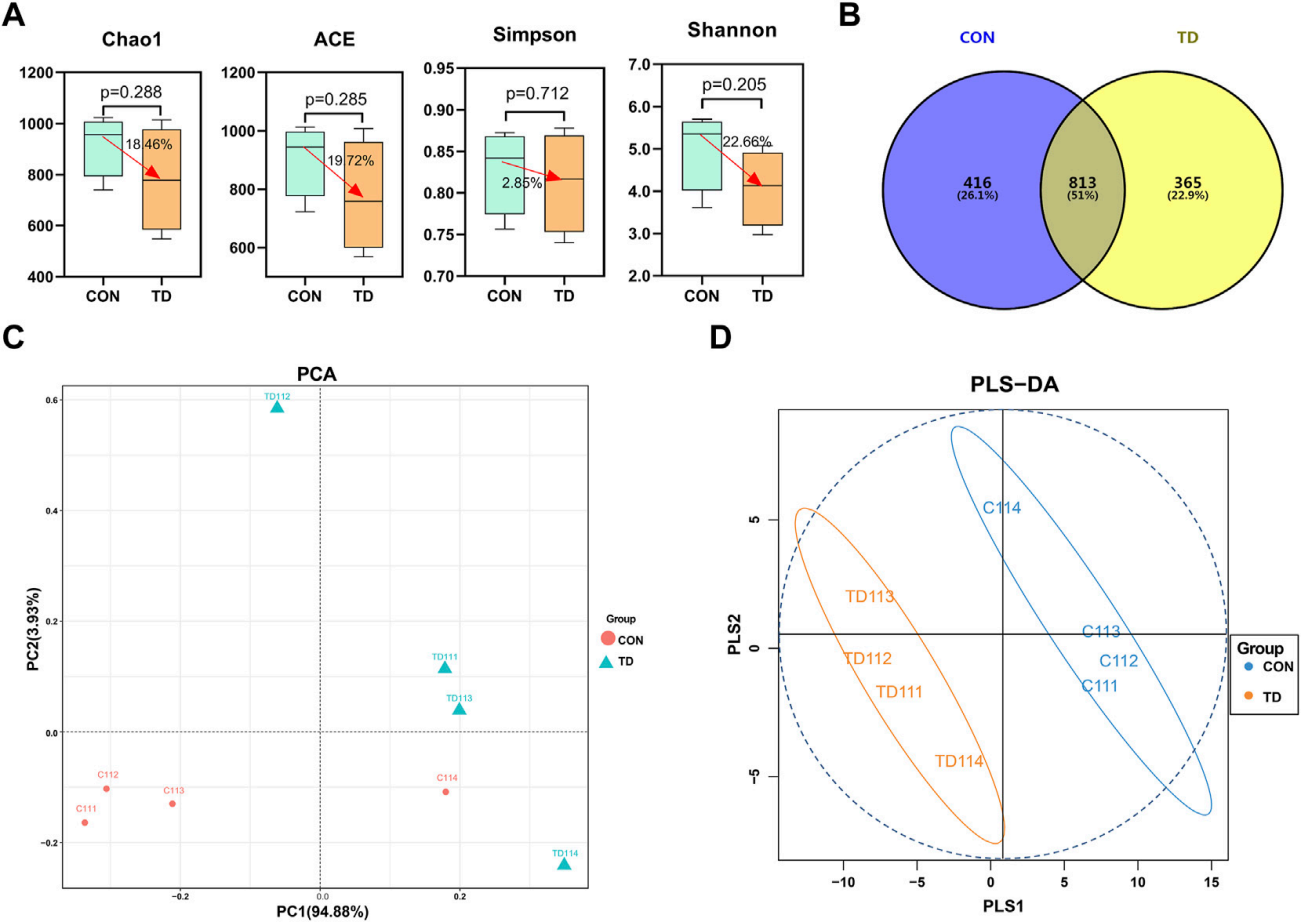
Fecal microbial overall structure of broiler chickens. **(A)** Chao1, ACE, Simpson, and Shannon indices from the CON and TD groups were used to analyze the alpha diversity. Statistical analysis was performed by Kruskal-Willis test, a *p*-value lower than 0.05 (<0.05) has statistically significant, N = 4. **(B)** The Venn diagram showed the common and unique OTUs in the CON and TD groups of broiler chickens. **(C)** Unweighted Unifrac PCA estimates for the gut microbiota of the CON group (red) and TD group (green). **(D)** OPLS-DA analysis of gut-microbiota in the CON group (green) and TD group (orange-yellow) of broiler chickens.

The analysis of tibia-related parameters showed significant changes in TD symptoms in broilers on days 11 and 15. Faecal samples are a non-invasive way to screen biomarkers for poultry (Zhu et al., 2021). Therefore, we further screen the potential biomarkers of TD broilers from 11 days of age. 16S rRNA gene sequencing examined the gut microbiota in the feces of both the CON and TD groups. The α-diversity analyzed using different indexes, including the Chao1 index, ACE index, Simpson index, and Shannon index, showed that there was decrease (18.46, 19.72, 2.85, and 22.66%, respectively) in the indexes of gut microbiota listed in the TD group, but the difference was not significant as compared with the CON group (*p* > 0.05) as shown in Figure 2A. The Venn diagram (Figure 2B) shows that 365 unique OTUs in the TD group compared with the CON group, accounting for approximately 22.9%. To analyze the differences between the CON and TD groups, the bioinformatics analysis methods of unweighted unifrac PCA and OPLS-DA model were performed to evaluate the gut microbiota in feces (Figures 2C,D). The constructed PCA and OPLS-DA score plots showed a clear separation between the CON and TD groups, indicating a significant difference in fecal microbial structure.

### Difference Analysis of Microbiota Composition Between the CON and TD Groups

**FIGURE 3:**
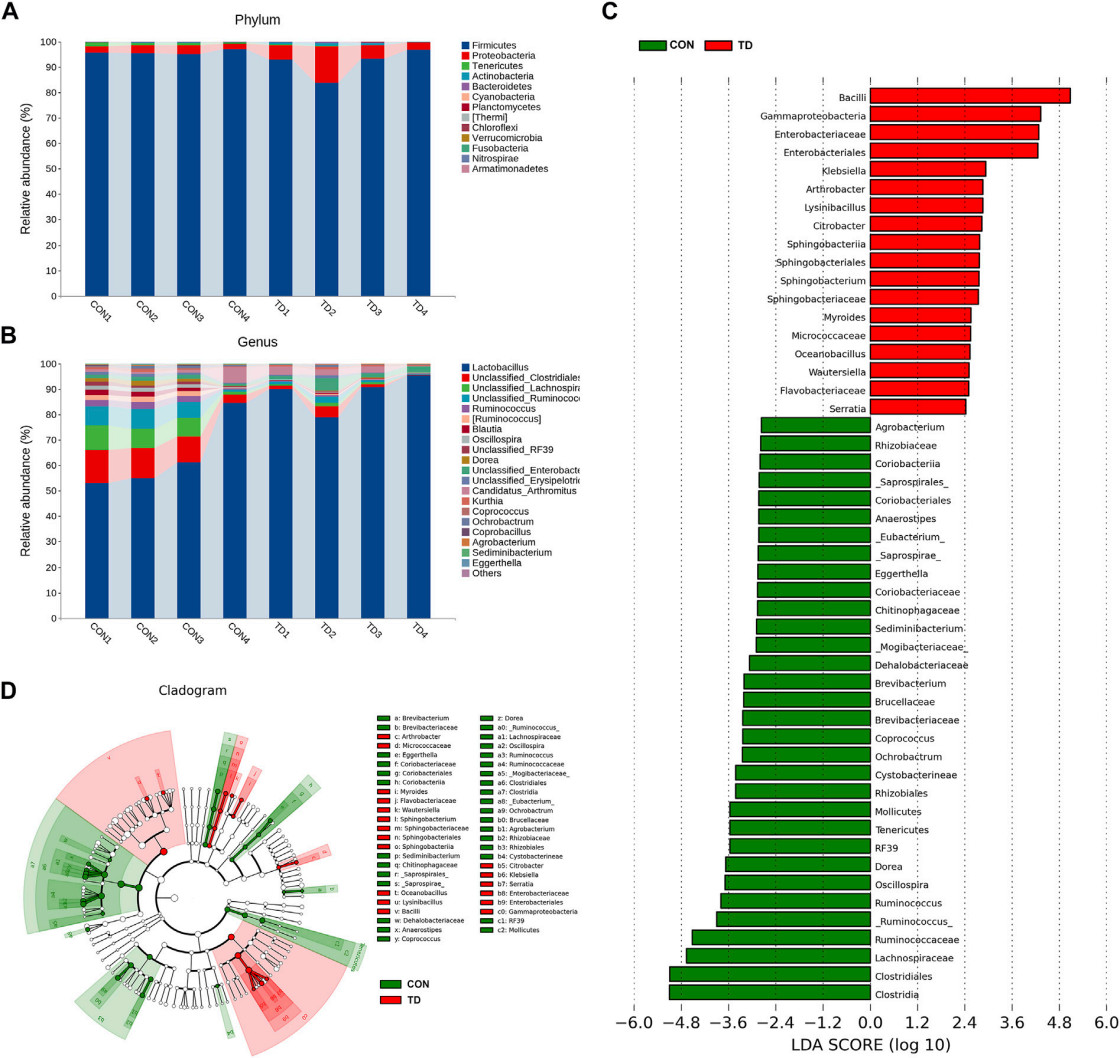
Fecal microbial composition of broiler chickens. **(A,B)** Relative abundance of microbiota at the phylum level and the genus level (Top 20) in CON and TD groups. **(C,D)** LDA score and cladogram of LDA effect size (LEfSe) comparison analysis between the CON and TD groups. As indicated, the red, green shading represents bacterial taxa that were significantly higher in either the CON or TD group. The selection of discriminative taxa between the CON and TD groups was based on an LDA score cutoff of 2.0, and differences in the relative abundances of taxa were statistically determined based on a Wilcoxon’s signed-rank test at a significance level of 0.05 (N = 4).

Next, we compared the relative abundance features of each group of bacterial taxa to distinguish specific alterations in microbiota. As shown in Figures 3A,B, five phyla with the highest average relative abundance, including the Firmicutes, Proteobacteria, Tenericutes, Actinobacteria and Bacteroidetes, and the proportion of the phyla Proteobacteria (2.82%), Actinobacteria (0.20%) and Bacteroidetes (0.18%) in the CON group was lower than in the TD group (7.09, 0.70, and 0.28%, respectively). Still, the phyla Firmicutes (95.82%) and Tenericutes (0.90%) in the CON group were higher than those in the TD group (91.72 and 0.13%, respectively) (see Supplementary Material S1). In addition, the genera between the CON and TD groups were distinctly enriched in *Lactobacillus*, *Ruminococcus* [*Ruminococcus*], *Blautia*, *Oscillospira*, *Dorea*, *Candidatus_Arthromitus*, *Kurthia*, *Coprococcus*, *Ochrobactrum*, *Coprobacillus*, *Agrobacterium*, *Sediminibacterium* and *Eggerthella* (Figure 3B, Supplementary Material S2).

Differential bacterial taxa between the two groups were further identified using the LEfSe analysis (Figures 3C,D), an algorithm for high-dimensional biomarker discovery and interpretation. The LDA was employed to determine the data and effects on the different species. LEfSe analysis identified 50 discriminative features (genus level; LDA score >2, *p* < 0.05), and the relative abundance was significantly different between the CON and TD groups. Among them, *Klebsiella, Arthrobacter, Lvsinibacillus, Citrobacter, Sphingobacterium, Myroides, Oceanobacillus, Wautersiella* and *Serratia* were concentrated in TD broilers; while *Ruminococcus*, *Oscillospira*, *Dorea*, butyrate-producing genera *Ochrobacturm*, *Coprococcus*, *Brevibacterium*, *Sediminibacterium*, *Eggerthella*, *_Eubacterium*, *Anaerostipes* and *Agrobacterium* were concentrated in normal broilers (see Supplementary Material S3). These results indicated that the fecal microbial composition of TD broiler chickens has been significantly changed compared to that of normal broiler chickens.

### Screening of Differential Fecal Microbiota in TD Broiler Chickens

**FIGURE 4:**
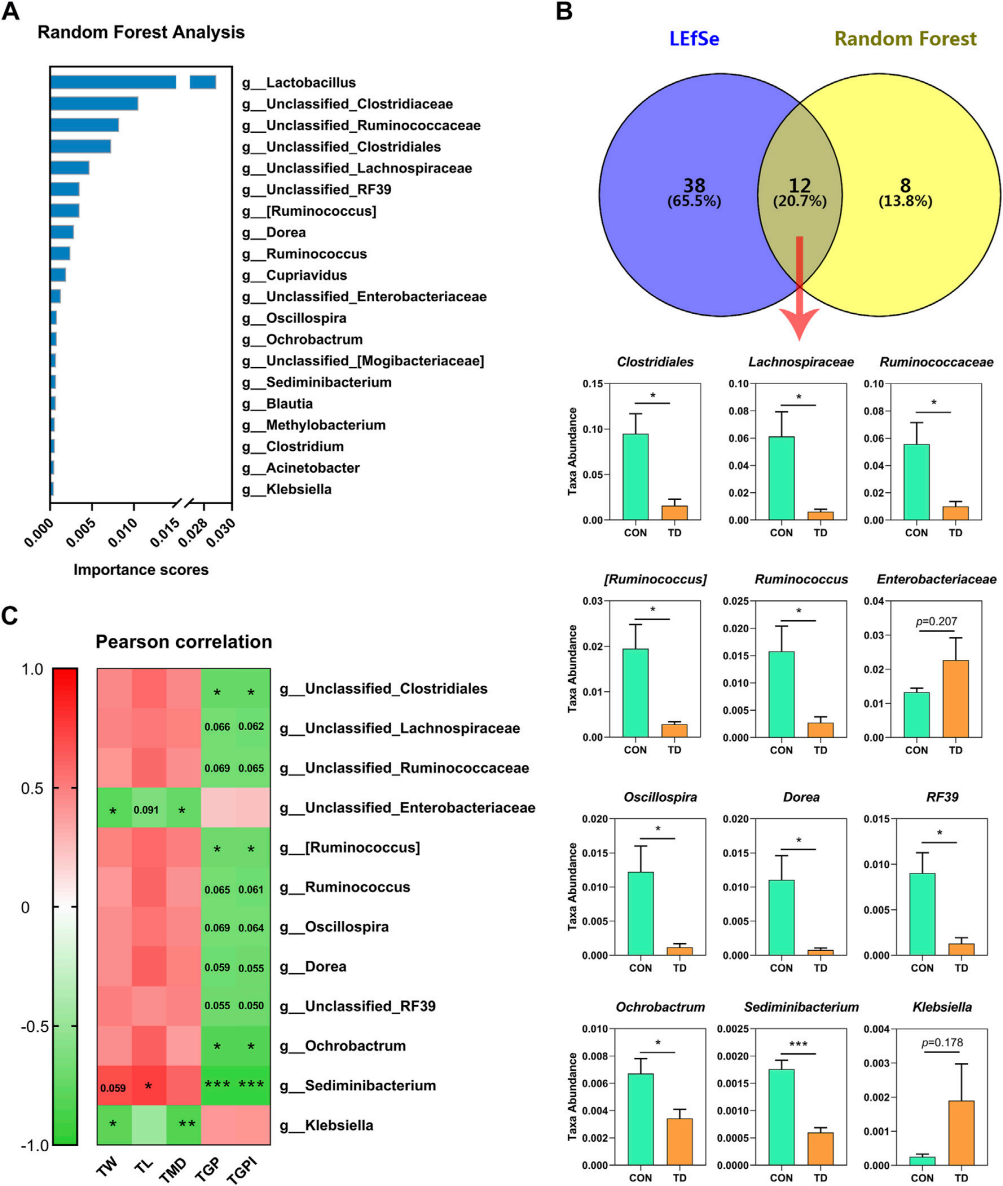
Analysis of differential fecal microbiota in broiler chickens with TD. **(A)** Random forest analysis on fecal microbiome of CON and TD groups of broiler chickens. The *y*-axis, from top to bottom, displays the taxa (TOP 20) ranked by their importance (Mean Decrease Accuracy) for the group classification. **(B)** Venn analysis was performed to screen differential fecal microbiota through LEfSe and random forest model. The overlapping part of the Venn diagram represents the number of differential fecal microbiota in common between groups, and statistical analysis was performed on the relative abundances of the twelve differential bacterial families between groups and significantly different in terms of abundance by the Student’s *t*-test (N = 4). The asterisks indicate statistically significant differences and correspond to *p* < 0.05 (*) and *p* < 0.001 (***). **(C)** Pearson correlation analysis between the differential fecal microbiota and tibia-related parameters of broiler chickens with TD. The asterisks indicate statistically significant differences and correspond to *p* < 0.05 (*), *p* < 0.01 (**), and *p* < 0.001 (***). A *p*-value higher than 0.05 (>0.05) represents not statistically significant.

The random forest analysis was performed to screen the top20 important species, showing the highest important species, including the *Lactobacillus,* Clostridiaceae*,* Ruminococcaceae*,* Clostridiales*,* Lachnospiraceae*, RF39 [Ruminococcus], Dorea, Ruminococcus, Cupriavidus,* Enterobacteriaceae*, Oscillospira, Ochrobactrum* [Mogibacteriaceae]*, Sediminibacterium, Blautia, Methyolbacterium, Clostridium, Acinetobacter* and *Klebsiella* (Figure 4A). In addition, Venn diagram analysis of the LEfSe identified 50 characteristic species (LDA score >2; *p* < 0.05) and the top 20 species in importance scores by random forest analysis showed that 12 species were screened, and we noted the higher abundances of Clostridiales (*p* = 0.013), Lachnospiraceae (*p* = 0.023)*,* Ruminococcaceae (*p* = 0.030) *[Ruminococcus]* (*p* = 0.020)*, Dorea* (*p* = 0.028), *Ruminococcus* (*p* = 0.032)*, RF39* (*p* = 0.016)*, Oscillospira* (*p* = 0.028)*, Ochrobactrum* (*p* = 0.043)*, and Sediminibacterium* (*p* < 0.001) in the TD group were clearly lower than those in the CON group, while the abundances of Enterobacteriaceae (*p* = 0.206) *and Klebsiella* (*p* = 0.178) in the TD group were increased compared with the CON group (Figure 4B). Next, the results of Pearson correlation analysis revealed that the tibia growth plate width and its index were correlated with Clostridiales (r = −0.728 and −0.736; *p =* 0.040 and 0.037, respectively) [*Ruminococcus*] (r = −0.710 and −0.717; *p =* 0.048 and 0.045, respectively)*, Sediminibacterium* (r = −0.913 and −0.917; *p =* 0.002 and 0.001, respectively)*, Ochrobactrum* (r = −0.813 and −0.811; *p =* 0.014 and 0.015, respectively) as shown in Figure 4C. In addition, tibia-related indicators were positively correlated with *Sediminibacterium* (r = 0.689, 0.742 and 0.605; *p =* 0.059, 0.035 and 0.112, respectively), and negatively correlated with Enterobacteriaceae (r = −0.799, −0.634 and −0.743; *p =* 0.017, 0.091 and 0.035, respectively) *and Klebsiella* (r = −0.806, −0.461 and −0.846; *p =* 0.016, 0.250 and 0.008, respectively) (Figure 4C). These results indicated that tibial growth and tibia growth plate quality are closely related to the differential faecal microbiota of TD broiler chickens.

### Analysis of Fecal Differential Metabolites of TD Broiler Chickens

**FIGURE 5:**
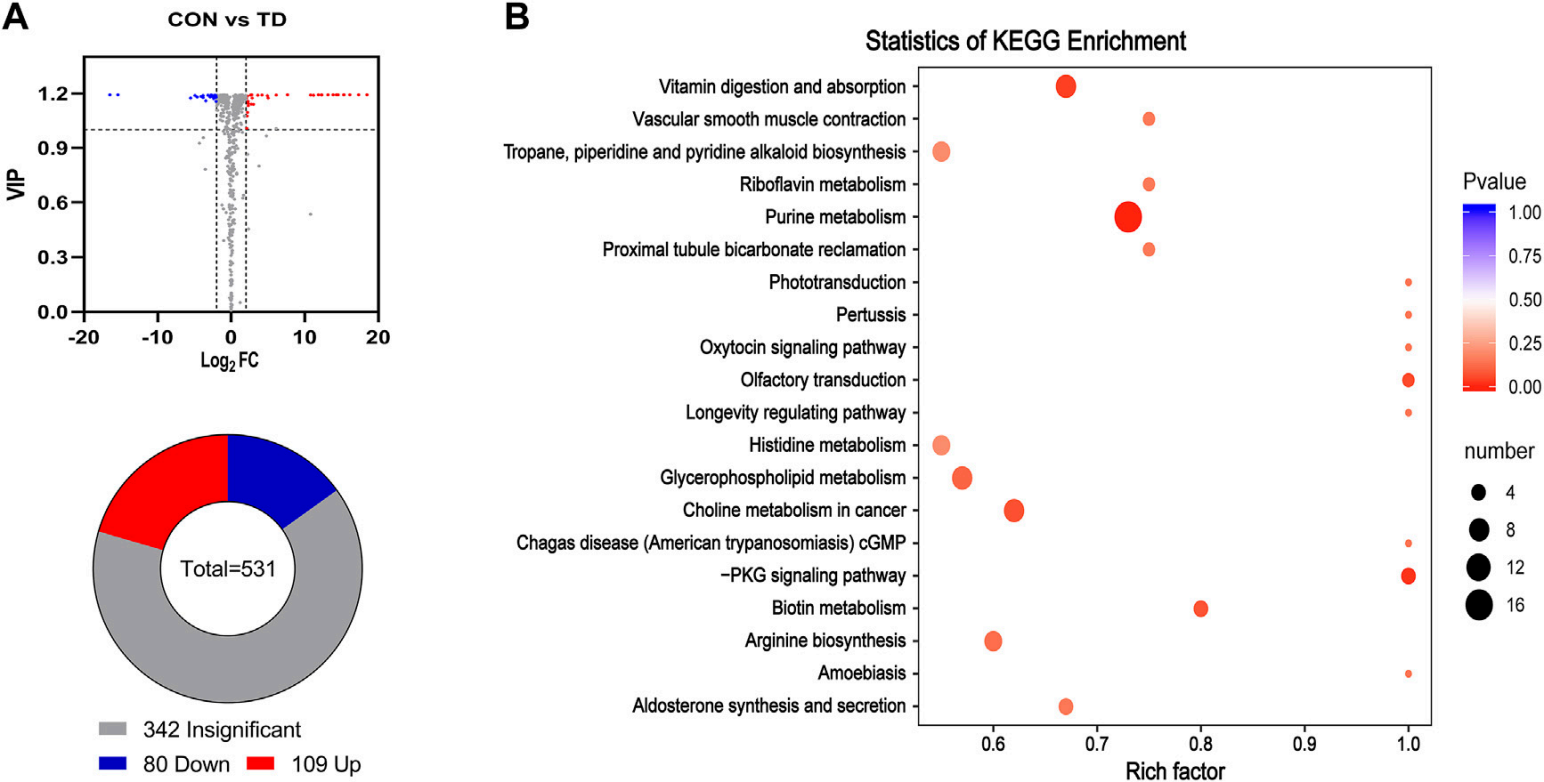
Differential metabolites in feces of broiler chickens with TD. **(A)** Volcano plot analysis of fecal metabolites. Some significantly differential metabolites were selected out by using the criteria of FC ≧2 or FC ≦ 0.5 and VIP≧1 in the volcano plot. Significantly differential metabolites were shown as a red (up) or blue (down) dot, whereas a gray dot represented non-significant difference of metabolites. The number of differential metabolites is shown using a pie chart. **(B)** Potential metabolic pathways analysis based on significantly differential metabolites in feces from the CON and TD group chickens. The bubble size indicates the number of significant differential metabolites enriched in this pathway, and the point with the different gradation of color (from red to blue) represents the scope of *p*-value. The bigger size of each circle indicates a higher degree of enrichment, and the lower *p*-value represents a more significant degree of enrichment.

The gut microbiota identified by 16S rRNA gene sequencing can only reach the genus level and has limitations as a potential biomarker, but metabolites derived from differential faecal microbiota become the optative choice (Kim et al., 2020). Next, to explore the composition of fecal metabolites, metabolomic analysis of the faecal samples was performed using UPLC-MS/MS. The results showed that a total of 531 metabolites were identified between the CON and TD groups (see Supplementary Material S4). PCA analysis revealed that the three biological replicates from the two groups clustered together in different regions, indicating significant differences in metabolism between the two groups (see Supplementary Figure S1A). Additionally, the supervised OPLS-DA method was performed to assess the faecal samples. The OPLS-DA score plot showed a clear separation with high reliability using the permutation test (Q2Y = 0.998) between the CON and TD groups (see Supplementary Figure S1B). The differential metabolites of the comparison groups were screened by combining the criteria of fold-change ≥ 2 or ≤0.5 and VIP value ≥1 of the metabolites. The results presented a total of 189 differentially produced metabolites, including 80 downregulated and 109 upregulated, as illustrated in the Volcano plot and Pie chart (Figure 5A, see Supplementary Material S5). Meanwhile, these differential metabolites between the two groups were mapped to the KEGG database for functional clustering analysis, and the results showed that the differentially expressed metabolites were mainly enriched in purine metabolism, vitamin digestion and absorption, glycerophospholipid metabolism, cGMP-PKG signaling pathway, biotin metabolism, etc. (Figure 5B).

**FIGURE 6:**
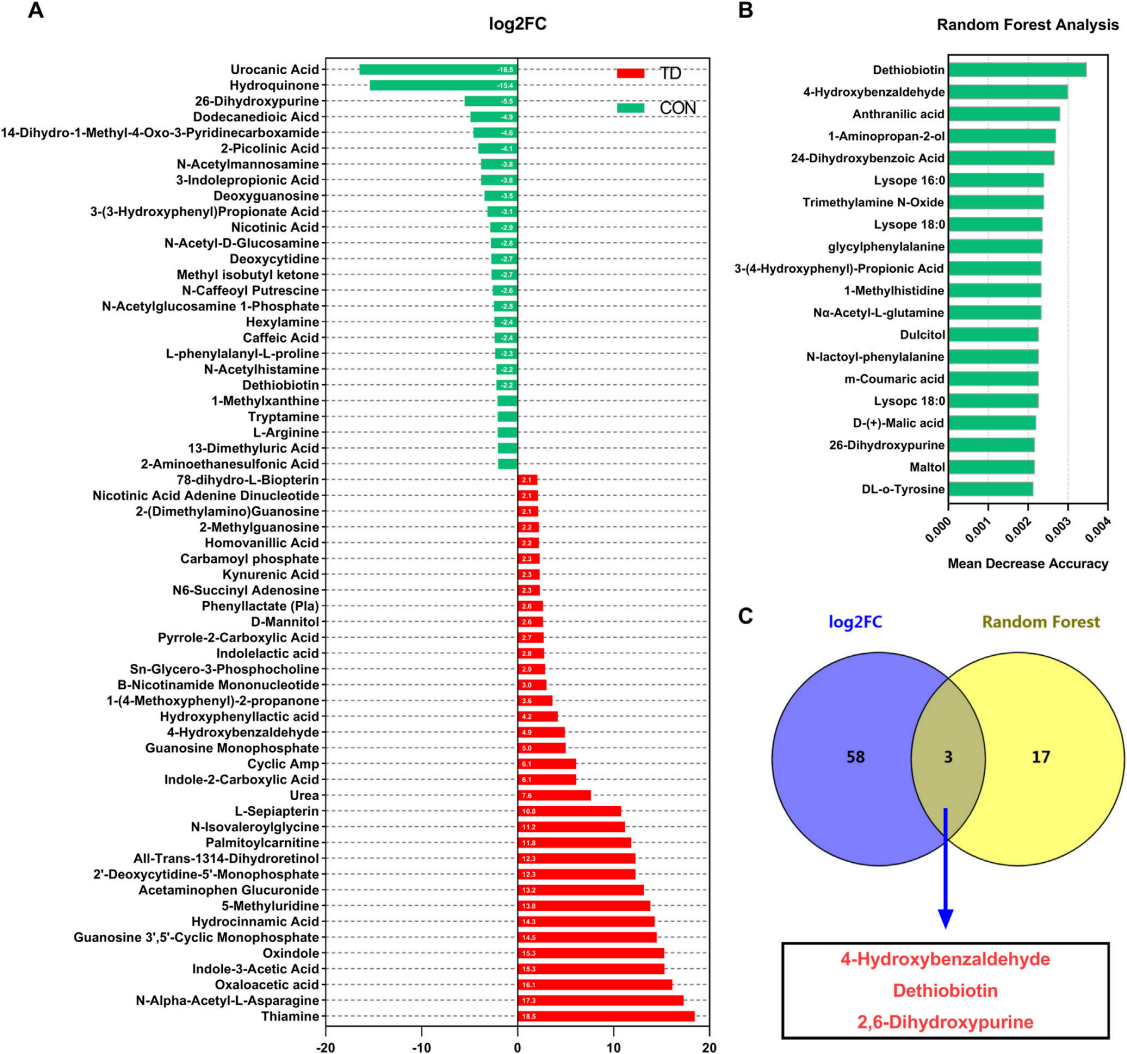
Screening of differential metabolites in feces of broiler chickens. **(A)** Visualization of log2-transformed read counts of differentially metabolites in feces between the CON and TD groups (|log2(FC)|≥2.5 and *p* < 0.05, FC stands for fold-change, N = 4). **(B)** Random forest analysis of differential metabolites in feces of broilers in the CON and TD groups. The *y*-axis, from top to bottom, displays the taxa (TOP 20) ranked by their importance (Mean Decrease Accuracy) for the group classification **(C)** Venn analysis was performed to screen differential fecal metabolites through log2FC and random forest model. The overlapping part of the Venn diagram represents the number of differential fecal microbiota in common between groups.

The difference analysis was used for comparisons of differential metabolites and the log2 fold change (log2FC ≥ 2.5 or ≤ −2.5) represented the comparison against the reference group based on OPLS-DA (Figure 6A). Compared with the CON group, the differential metabolites with the highest abundance in the TD groups were the thiamine (log2FC = 18.46), N-alpha-acetyl-L-asparagine (log2FC = 17.31), oxaloacetic acid (log2FC = 16.12), indole-3-acetic acid (log2FC = 15.31), oxindole (log2FC = 15.28), guanosine 3′,5′-cyclic monophosphate (log2FC = 14.50), hydrocinnamic acid (log2FC = 14.27), 5-methyluridine (log2FC = 13.84), acetaminophen glucuronide (log2FC = 13.17) and 2′-deoxycytidine-5′-monophosphate (log2FC = 12.27), while the content of urocanic acid, hydroquinone, 2,6-dihydroxypurine and dodecanedioic acid were lower (Figure 6A). These results indicated that the fecal metabolites of broilers with TD were significantly fluctuated. Then, the top 20 important metabolites were screened using random forest analysis, including the dethiobiotin, 4-hydroxybenzaldehyde, anthranilic acid, 1-aminopropan-2-ol, 2, 4-dihydroxybenzoic acid, lysope (16: 0), trimethylamine N-Oxide, lysope (18: 0), glycylphenylalanine, 3-(4-hydroxyphenyl)-propionic acid, 1-methylhistidine, Nα-acetyl-L-glutamine, dulcitol, N-lactoyl-phenylalanine, M-coumaric acid, lysopc (18: 0), D-(+)-malic acid, 2, 6-dihydroxypurine, daltol, DL-o-tyrosine, N-acetyl-L-tyrosine and biopterin (Figure 6B). Moreover, Venn diagram analysis of OPLS-DA (identified 61 characteristic metabolites (log2FC ≥ 2.5 or ≤ -2.5)) and random forest analysis (the top 20 important metabolites) showed that three potential biomarkers were screened, including 4-Hydroxybenzaldehyde, Dethiobiotin, and 2,6-Dihydroxypurine (Figure 6C).

### Predictive Ability of Fecal Metabolites for TD Broiler Chickens

**FIGURE 7:**
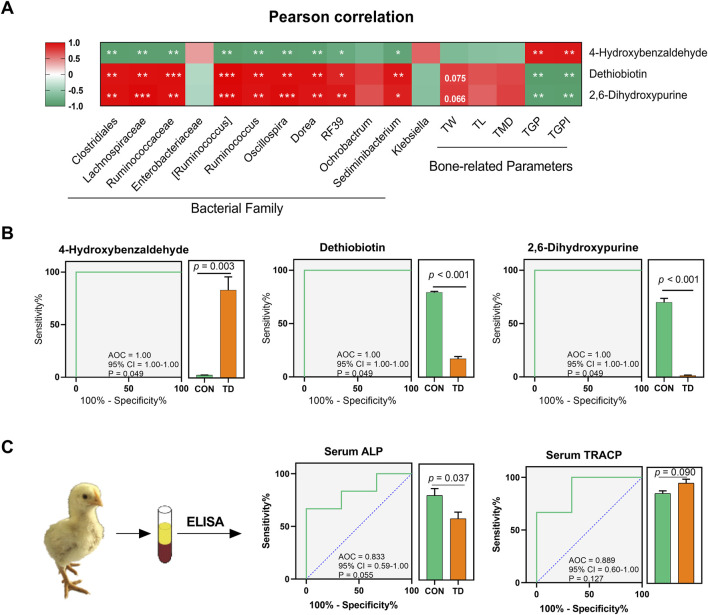
Fecal metabolite biomarkers for the diagnosis of TD broiler chickens. **(A)** Pearson correlation analysis between the differential microbiota and metabolites, differential metabolites and tibia-related parameters in feces of broiler chickens with TD. The asterisks indicate statistically significant differences and correspond to *p* < 0.05 (*), *p* < 0.01 (**), and *p* < 0.001 (***). A *p*-value higher than 0.05 (>0.05) represents statistically non-significant (N = 4). **(B)** Curves of ROC of differential metabolite biomarkers for diagnosis of TD in an independent test set. An AUC close to 1.0 indicates high sensitivity and specificity and statistical analysis was performed on the relative abundances of the three differential metabolites between groups by the Student’s *t*-test, a *p*-value < 0.05 was considered to be significant (N = 4). **(C)** ROC curve of serum ALP and TRACP for diagnosis of TD (N = 3).

Pearson analysis was performed to unveil the correlations between the abundance of 12 differential fecal microbiota, three differential fecal metabolites, and tibia-related parameters in the host (Figure 7A). The results revealed that dethiobiotin and 2,6-dihydroxypurine were positively correlated with the relative abundances of several important bacterial genera, including the Clostridiales, Lachnospiraceae, Ruminococcaceae [*Ruminococcus*], *Dorea*, *Ruminococcus*, *RF39*, *Oscillospira*, and *Sediminibacterium.* However, 4-hydroxybenzaldehyde was negatively correlated with these important bacterial genus (Figure 7A). More interestingly, the damage indexes (TGP and TGPI) of the tibial growth plate of TD broilers were negatively correlated with the differential metabolites dethiobiotin and 2,6-dihydroxypurine, whereas the damage indexes of the tibial growth plate were positively correlated with 4-hydroxybenzaldehyde (Figure 7A). These results indicated that damage to the tibia growth plate in TD broilers is associated with changes in differential metabolites, which is also closely related to the gut-microbiota in feces.

Receiver operating characteristic (ROC) curve analysis was performed to evaluate the predictive ability and diagnostic performance of differential fecal metabolites in distinguishing TD from normal broiler chickens. Three metabolites with higher importance scores among the differential fecal metabolites, namely 4-hydroxybenzaldehyde, dethiobiotin, and 2,6-dihydroxypurine, were screened and their ROC analysis showed high values of sensitivity, specificity and the area under the curve (AUC) (AUC = 1, *p* = 0.049; Figure 7B).

**FIGURE 8:**
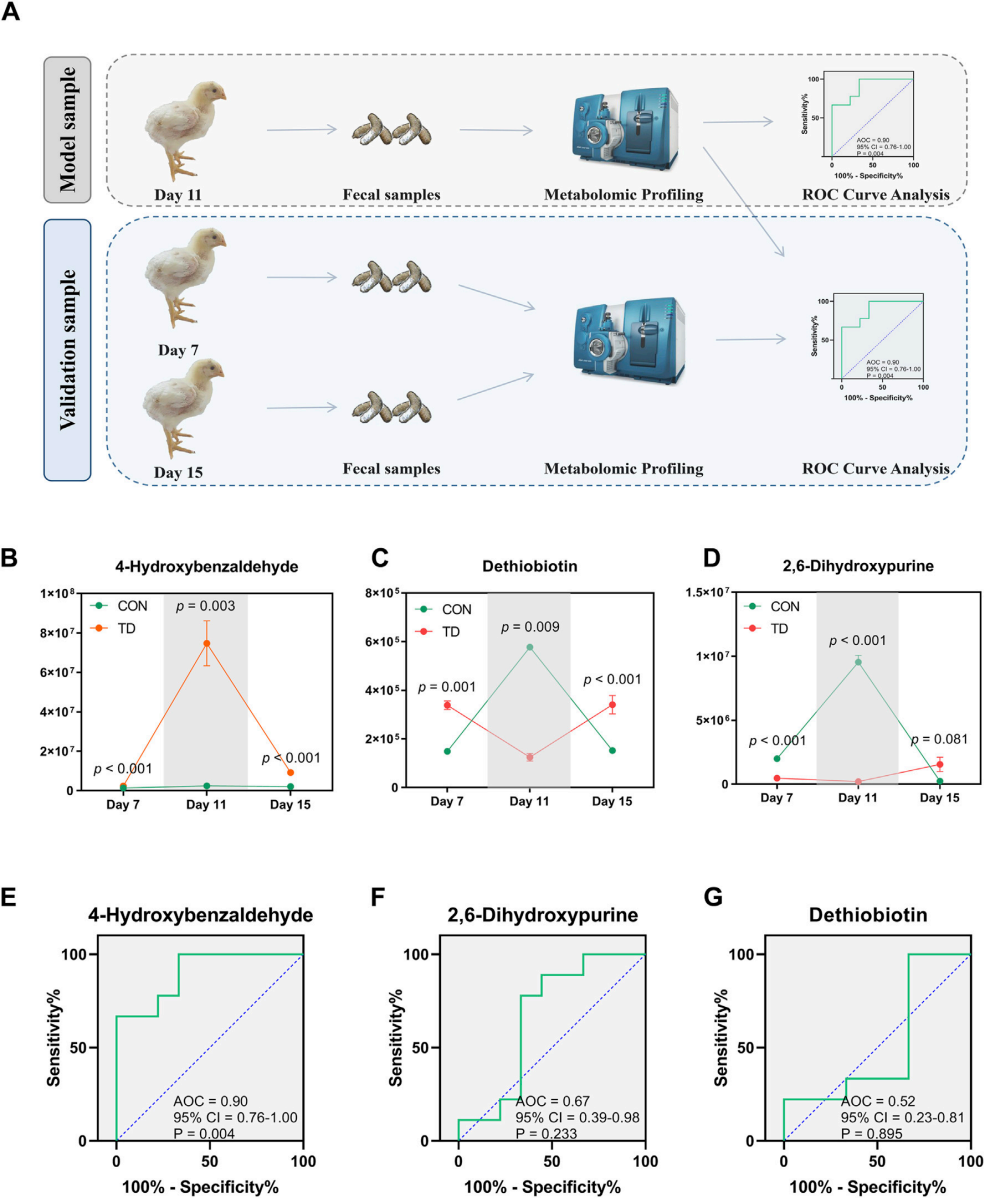
Validation and evaluation of the predictive power of the three metabolites. **(A)** Design of verification experimental approach. **(B–D)** Statistical analysis was performed on the relative abundances of the three differential metabolites between groups by the Student’s *t*-test, a *p*-value < 0.05 was considered to be significant (N = 4). **(E–G)** Curves of ROC of differential metabolite biomarkers for diagnosis of TD during the entire trial. An AUC close to 1.0 indicates high sensitivity and specificity, a *p*-value < 0.05 was considered to be significant (N = 4).

Serum ALP and TRACP are important indicators for the assessment of normal bone function. Serum ALP secreted by osteoblast has been extensively studied as a biomarker of bone disease, which is essential for osteoid formation and mineralization. TRACP secreted by osteoclasts is a sensitive index of bone resorption, and its activity correlates with resorptive activity in bone diseases (Li et al., 2010). ROC curves of bone funtion biomarkers are shown in Figure 7C, the AUCs of the serum ALP and TRACP were 0.83 and 0.89, respectively; these serum biomarkers can distinguish the TD from normal broilers, which is lower than the predictive ability of the differentially fecal metabolites. To further validate the predictive ability of these three biomarkers, we sequenced the feces from normal and TD broilers on days 7 and 15 based on metabolomics (Figure 8A). Verification and evaluation of the predictive ability in the three potential biomarkers were assessed based on ROC curve analysis, the results showed that the abundance of 4-hydroxybenzaldehyde, dethiobiotin, and 2,6-dihydroxypurine were significantly different (*p* < 0.05; Figures 8B–D) and their AUCs were 0.90 (95% CI = 0.76–1.00; *p* = 0.004), 0.52 (95% CI = 0.23–0.81; *p* = 0.895), and 0.67 (95% CI = 0.39–0.98; *p* = 0.233) during the entire trial, respectively (Figures 8E–G). These results indicated that differential fecal metabolites, especially 4-hydroxybenzaldehyde, which is closely related to differential gut-microbiota, could be used as a valuable biomarker for the assessment and diagnosis of broiler chickens with TD.

## Discussion

In this study, we investigated the faecal gut microbiome structure and composition of normal and TD broilers as well as the changes of fecal metabolites by metabonomics, and further analyzed for correlation with tibiae and their growth plate-related indexes. The results indicated significant differences in the overall structure and composition of fecal microbiota between the two groups. The proportion of the phyla Firmicutes and Tenericutes in the TD broilers was lower than that in the normal broilers. Furthermore, broiler chickens with TD are characterized by enriched *Klebsiella*, and depleted genera *[Ruminococcus*]*, Dorea, Ruminococcus, Oscillospira, Ochrobactrum, and Sediminibacterium,* as well as disturbances of fecal purine metabolism, vitamin metabolism, and amino acid metabolism. Interestingly, the abundance of [*Ruminococcus*]*, Sediminibacterium, and Ochrobactrum* was negatively correlated with tibia growth plate width and tibia-related parameters. Tibia related-parameters were positively correlatied with *Sediminibacterium,* whereas negatively correlated with *Klebsiella,* which provides information on abnormal growth and development of tibias and their growth plates in TD broilers associated with changes in the gut microbiota affecting the metabolism of fecal metabolites. We further screened three fecal differential metabolites closely associated with the differential microbiota and tibia related-indicators based on log2FC and random forest analysis for ROC analysis, which were highly specificity and superior to serum bone markers. Furthermore, verification and evaluation of the predictive ability of the three potential biomarkers during the entire trial found that the AUC of 4-Hydroxybenzaldehyde was 0.90. Therefore, our findings indicated that gut microbiota disturbances or dysbiosis could potentially contribute to TD pathogenesis by modulating abnormal production of fecal metabolism, and fecal metabolite 4-Hydroxybenzaldehyde as a biomarker may be valuable for the assessment and diagnosis of broiler chickens with TD.

Broiler chickens are a poultry breed that is very susceptible to stress due to the genetic selection of broilers for rapid growth and increased breast yields. Therefore, some invasive diagnostic methods are not certainly the best choice in broilers, especially for diagnosing the TD broilers accompanied with sub-clinical symptoms. There is growing evidence that biomarker discovery is a key target for many microbiome and metabolome studies as they implicate potentials for developing sensitive, non-invasive, and scalable early diagnosis (Tsoukalas et al., 2021; Zhu et al., 2021). Moreover, abnormal bone development or pathological bone loss is closely associated with alterations in microbiota composition (Castaneda et al., 2020; Lu et al., 2021). In the present study, we noted that the width of the tibial growth plate, mainly contributed by TD broilers, accounted for the largest proportion of all bone phenotypes. The most significant changes in all bone phenotypes occurred on days 11 and 15 using morphological observation and Sankey diagram analysis. Accordingly, the feces of TD broilers on day 11 were more representative for subsequent biomarker screening.

The richness and diversity of the intestinal bacterial species are essential elements of the animal intestinal microbiome (McKinney et al., 2020). In the current study, the alpha diversity of the faecal microbial community was found to be significantly lower in the TD group than in the CON group by faecal microbiome sequencing. Moreover, the structure and composition of the faecal microbiome of the TD and CON groups were markedly different, indicating the presence of gut microbial dysbiosis in TD broilers (Zhu et al., 2021). Previous studies have revealed that gut microbiota homeostasis is among the most important factors for the host to resist the invasion of pathogenic bacteria and perform various biological functions. Conversely, dysbacteriosis has emerged as a hidden risk factor that can generate bioactive metabolites, enzymes, or small molecules that affect host physiology and pose a great threat to host health (Biver et al., 2019; Wang et al., 2019; Meng et al., 2020). Although the gut microbiota changes dynamically, significant alterations in the microbial community can cause dysbacteriosis and affect intestinal barrier function (Liu et al., 2020). In addition, gut-derived bacterial metabolites can penetrate through the gut into the bloodstream disrupting the bioenergetic machinery of host cells, which provides an essential pathway for the gut microbiota to regulate anatomically distant organs (Lu et al., 2021; Tomasova et al., 2021). Therefore, the effect of changes in microbiome structure and composition on bone physiological functions has gradually attracted attention (Chen et al., 2017; Castaneda et al., 2020; Hao et al., 2021; Ling et al., 2021).

In the this study, the category of predominant phyla in the faecal microbiome of broiler chickens were Firmicutes, Proteobacteria, and Tenericutes, which are different from mammals and humans and indicate a special gut microbiota in broilers (Sánchez-Alcoholado et al., 2020; Zhang et al., 2020). Among them, Firmicutes is mainly composed of Gram-positive bacteria, such as *Lactococcus* and *Lactobacillus, etc.,* most of which are considered to be beneficial bacteria that contribute to maintain the balance of gut microbiota and prevent pathogenic invasion (Garneau et al., 2008). More meaningful for us is that we noticed a relatively low proportion of the phyla Firmicutes and Tenericutes in TD broilers compared with the normal broilers. Moreover, TD broiler chickens were characterized by enriched genus *Klebsiella*, and depleted the genera [*Ruminococcus*]*, Dorea, Ruminococcus, Oscillospira, Ochrobactrum, and Sediminibacterium,* as well as disturbances of fecal purne, vitamin, and amino acid metabolism. The Gram-negative *bacillus* of the genus *Klebsiella* is widely distributed in the gastrointestinal tract and respiratory tract, which may result in organ inflammation, and even the septicemia (Fendukly et al., 2003).

In contrast, the genus *Ruminococcus* can effectively decompose and ferment the cellulose, hemicellulose, and polysaccharides in foods into acetate and succinate (La Reau and Suen, 2018), and *Ochrobactrum* strains can indirectly influence the propionic acid decomposition through the production of vitamin B12, which plays a crucial role in maintaining the functionality and morphology of intestinal epithelial cells, regulating systemic bone mass and preventing pathological bone loss (Watson et al., 2016; Wang et al., 2017; Lucas et al., 2018; Zimmermann et al., 2019). A recent publication has highlighted that lower enrichment of Ruminococcaceae was negatively correlated with the presence of osteoporosis, which is consistent with our findings and provides strong evidence for an association between gut microbiota and bone in humans (Ling et al., 2021). In addition, the genus *Sediminibacterium* has been reported to be closely associated with fat deposition in chickens (Kong et al., 2020). The decrease of these genera in TD broilers will inevitably affect the normal function of the intestinal tract and the production of SCFAs, which may potentially contribute to the pathogenesis of TD. More interestingly, the abundance of [*Ruminococcus*]*, Sediminibacterium,* and *Ochrobactrum* was negatively correlated with the width of the tibia growth plate and its index TGPI. Tibia related-parameters were positively correlated with *Sediminibacterium* and negatively correlated with *Klebsiella,* which further provides evidence that abnormal growth and development of the tibiae and their growth plates in TD broiler chickens are associated with changes in the gut microbiota and affect the metabolism of fecal metabolites.

The results demonstrated that the metabolites of TD broilers are different from those of normal broilers, especially amino acid metabolomics, organic acid derivatives, indole derivatives, vitamins, and carbohydrate metabolomics. Previous studies have shown that gut metabolites affect the balance of intestinal microecology and regulate anatomically distant biological effects by penetrating from the intestine into the bloodstream (Lu et al., 2021; Tomasova et al., 2021). Indole derivatives were first described as one of the microbiota-derived metabolites that contribute to intestinal homeostasis in humans and animals by modulating the immune function of the host (Schiering et al., 2017; Xiao et al., 2020). The previous studies have described the skeletal effect of SCFAs, as key regulatory metabolites produced by the gut microbiota, showing that butyrate promotes mineralized nodule formation and osteoprotegerin expression (Katono et al., 2008; Lu et al., 2021). Next, 189 fecal differential metabolites, mainly enriched in purine metabolism, vitamin metabolism and amino acid metabolism, etc., were screened and closely correlated with the differential gut microbiota and tibia-related indicators. In birds, uric acid, a purine compound, serves as the end product of nitrogen catabolism originating from the microbes, is rapidly cleared from the blood by the kidneys. Purines have been shown to be involved in continuous bone remodeling by bone cells, allowing the skeleton to grow and repair itself, which is achieved in a balance of osteoclasts and osteoblasts, eventually purine metabolism likely contributing to dynamic bone homeostasis (Schwartz, 1978; Stentoft et al., 2015; Zuccarini et al., 2021). Studies on the vitamin metabolism affecting bone growth and development mainly focused on vitamin D, which is associated with osteoporosis (Binkley, 2012). Besides, vitamin B, such as thiamine, folate (vitamin B9), and cobalamin (vitamin B12), are also associated with bone metabolism and bone quality, and contribute to fracture risk by affecting homocysteine/folate metabolism (Ahn et al., 2020). An emerging report through fecal and serum metabolomics analyses also implies that amino acid metabolism is clearly associated with the identified microbial biomarkers and osteoporosis, respectively (Ling et al., 2021). Therefore, these enriched metabolisms are closely related to bone homeostasis, and the occurrence of TD in broilers may be associated with metabolite changes in related pathways. The detection of relevant differential metabolites may be a biomarker for early diagnosis or prediction of TD in broilers.

ROC curves are currently used as a fundamental tool for medical diagnostics (Zheng et al., 2019; Wang et al., 2020), and are rarely used in veterinary medicine. Here, we selected the three fecal metabolites based on the log2FC and the random forest analysis, and the isolated metabolic markers could robustly distinguish TD broilers from normal broilers. Furthermore, the separate metabolic markers showed extremely high specificity in TD broilers, better than serum bone markers, including serum ALP and TRACP. Further validation revealed that 4-hydroxybenzaldehyde was significantly increased in TD broilers and had a higher specificity throughout the broilers suffering from TD. These findings suggested that separate fecal metabolic biomarker has a non-invasive diagnostic potential for TD.

Despite many novel insights, our study has certain limitations. First, our study results cannot conclusively determine the causal relationship between the imbalance of faecal microbial community and TD, and the causal contribution of the gut microbiota and its corresponding metabolites to TD. Therefore, a multitude of more direct experimental evidence is needed for further verification, such as gut microbiota transplantation study, intervention study of differential metabolites, etc. (Witkowski et al., 2020). In addition, further animal experiments are needed to explore how the interactions between microbial species and metabolites, specific gut microbiota-dependent regulatory pathways, and downstream differential metabolites may affect TD pathogenesis. The 16S rRNA sequencing technology has relatively limitations in this study. Therefore, further experiments will use larger cohorts and integrated data of different levels to analyze the taxonomy, composition, pathways, and functions of gut microbiome in TD broiler chickens using a high-resolution shotgun metagenomic data sequencing approach. On these foundations, robust microbial biomarkers were established to diagnose TD in broiler chickens. Furthermore, it is important to collect more clinical faecal samples from nature TD broilers to screen the differential gut microbiota and fecal metabolites and verify our existing findings. Notably, the fecal sample sampling process is susceptible to interference from other factors and therefore requires a high level of fecal sample collection process. These are crucial to optimize biomarkers and to evaluate the sensitivity and accuracy of predicting the occurrence of TD in chickens.

In conclusion, we summarized the characteristics and interaction analysis of altered tibia related-parameters and their growth plate morphology, faecal microbiota, and metabolites in TD broilers by using multi-omics data. We found that the dysbiosis of the gut microbiota may participate in the pathogenesis of TD by regulating the host’s fecal metabolism. Moreover, we identified 4-hydroxybenzaldehyde as a key biomarker to distinguish TD broilers from normal broilers and to assess the severity of boiler chickens suffering from TD. Our findings may provide a new potential path for understanding the pathogenesis of TD, and it is possible to develop a non-invasive and non-stress tool for early diagnosis of broiler TD.

## Materials and Methods

### Animals

A total of 180 one-day-old healthy Arbor Acres (AA) broilers were purchased from an intensive commercial broiler farm located in Kaifeng City (Henan, China). They were housed in cages in the livestock laboratory of the Henan Agricultural University (Henan, China). The chickens were reared and performed according to the standard AA broiler management manual as previously described (Huang et al., 2018).

All animal experiments and procedures were performed in strict accordance with the guidance of the Animal Care and Use Committee of Henan Agricultural University and the Care and Use of Laboratory Animals of the College of Veterinary Medicine (approved no: 17–0126), none of the chickens exhibited signs of distress before their death during the experiment. Besides, all the possible measures were carried out to ensure the welfare of the broiler chickens.

### TD Establishment

All broilers were allowed to adapt to the environment before the further experiment. Broilers were randomly divided into two separate groups: 15 chicks per cage, total four replicates per group. The grouping of the chickens was as: the control group (CON) and the thiram-induced TD chicken group (TD; the addition of 100 mg/kg of thiram from day 4 to day 7, then followed by feeding with a normal diet until the end of the experiment). All experiments lasted for 15 days, with *ad libitum* provision of feed and water.

### Sample Collection

Ten broilers were randomly selected from each group for weighing on days 7, 11 and 15 of the experiment, respectively. Blood samples were obtained from the wing vein using heparinized syringes and the serum sample was centrifugated at 3,000 rpm for 10 min at 4°C and stored at −20°C.

Chicks were sacrificed by cervical dislocation in the CON and TD groups. Then, tibia specimens were retrieved for measurement of tibia-related parameters, and the growth plate was stripped from the articular cartilage of the proximal tibia using a surgical knife for morphological analysis of the tibial growth plate as in our previous studies (Huang et al., 2017; Huang et al., 2018). Furthermore, faecal samples were collected from the chickens of both groups on days 7, 11, and 15 to analyze the microbiome and metabolome. The sterile cotton swabs were used to take the upper half of freshly removed chicken feces (within 5 min) in a 2 ml sterile eppendrof (EP) tube, mixed and stored at −80°C. To reduce the difference in sampling time, the first group was sampled in turn until the last group was sampled. We mixed the faeces of 4–6 different broilers into one faecal sample and used four mixed faecal samples per group for subsequent 16S rRNA sequencing and metabolomic analyses to reduce sampling error.

### Measurement of Tibial Parameters

All tibial specimens from chinkens were collected to analyze the weight (g), length (mm), mean diameter (mm) and growth plate width (mm) of the tibia. These bone histomorphometry parameters were determined by an electronic balance sensitive to 0.001 g (#FA1204N; Jinghai Instrument Co., Ltd. Shanghai, China) and Digital Calipers (#SATA91511; TATA Company, Shanghai, China), respectively. In addition, the tibia growth plate index (TGPI, mm/mm) was determined as the width of the tibia growth plate in the broiler. Tibia mean diameter was calculated as the average of the TUD (upper 1/2 diameter of the tibia), TMD (tibia mid-diameter), and TLD (lower 1/2 diameter of the tibia) (Figure 1A).

### Analysis of Serum Biochemical Parameters

To determine the levels of calcium (Ca) and phosphorus (P) in serum samples from the CON and TD groups, the electrolyte indicators were quantified by a veterinary reagent tray (Chengdu Puli Taisheng Technology Co., Ltd, Chengdu, China). Quantitative assays were performed with a portable, fully automated veterinary biochemistry analyzer (#SMT-120 V; Seamaty Technology Co., Ltd, Chengdu, China).

### Quantification of TRACP and ALP in Serum

Serum concentrations of alkaline phosphatase (ALP) and tartrate-resistant acid phosphatase (TRACP) were measured by an enzyme-linked immunosorbent assay (ELISA) kit (Nanjing Jiancheng Biotechnology Co. Ltd, Nanjing, China) according to the manufacturer’s instructions. The optical density values of each well were determined within 15 min at a wavelength of 450 nm using microplate reader (Model: 30086376 Spark, Tecan Group Ltd, Mannedorf, Switzerland).

### Analysis of 16S rRNA Gene Sequencing

According to the manufacturer’s instructions, faecal microbial genome DNA was extracted using a QIAamp DNA tool mini kit (Qiagen, Hilden, Germany). The V3 and V4 hypervariable regions of the bacterial 16S rRNA were PCR-amplified using bacterial primers 338 (forward: 5′-ACT​CCT​ACG​GGA​GGC​AGC​A-3′) and 806 (reverse: 5′-GGACTACHVGGGTWTCTAAT-3′). The amplicons were purified and quantified using Agencourt AMPure Beads and the PicoGreen dsDNA Assay Kit (Invitrogen, Carlsbad, CA, United States), respectively. TheDNA libraries were constructed and then sequencing was performed on an Illumina Miseq 250 platform (Illumina Inc. San Diego, CA, United States) according to the standard protocols by Shanghai Personalbio Technology Co., Ltd, China. Raw Illumina read data for all samples were deposited in the NCBI Sequence Read Archive with the accession code PRJNA755075.

As previously described, the bioinformatics analysis procedure of raw reads was performed (Kong et al., 2020). The bacterial community was compared with their beta diversity using the distance matrices generated from the principal component analysis (PCA) and partial least squares discriminant analysis (PLS-DA). The key bacterial taxa responsible for discrimination between the two groups were identified using linear discriminant analysis (LEfSe) with linear discriminant analysis (LDA) score >2.0 based on the Galaxy online analysis platform (http://huttenhower.sph.harvard.edu/galaxy/). The random forest classifier was performed by 10-fold cross-validation to predict the discrimination between the CON and TD groups in broilers.

### Analysis of Widely Targeted Metabolomics

Metabolomic analysis was conducted by a commercial service company (Wuhan Metware Biotechnology Co., Ltd, Wuhan, China) following previously reported methods (Huang et al., 2021). Briefly, 50 mg faecal samples were processed with 1,000 μL methanol/water (ice-cold, 70%, v/v) and 5 μl 2-chlorophenylalanine (1 μg/ml). Two pre-cooled sterile steel balls were added to the sample mixture at −20°C for 2 min and homogenized at 30 Hz for 3 min. The sample mixture was centrifuged at 12,000 g for 10 min at 4°C. The supernatant was collected and filtered through a 0.22 μm filter membrane, and quality control (QC) analysis of samples was performed before UPLC-MS/MS analysis (see Supplementary Material S2).

All data analyses were based on the self-built MWDB database (Metware Biotechnology Co., Ltd. Wuhan, China). PCA analysis and orthogonal partial least squares discriminant analysis (OPLS-DA) were performed for the identified metabolites. UPLC-MS/MS analyses were performed, and differentially accumulated metabolites with fold change (FC) ≥ 2 or ≤0.5 and variable importance in project (VIP) ≥ 1 were used as criteria for screening potential biomarkers. In addition, KEGG annotation and enrichment analysis were performed according to our previously described methods [64].

### Statistical Analysis

GraphPad Prism version 8 (GraphPad Software, La Jolla, CA, United States) was used to perform statistical analysis and drawing. All data are presented as the means ± SD. Comparisons between groups were conducted using two tailed unpaired student’s *t*-test. Correlations between the altered gut microbes, significantly different faecal metabolites, and tibia related-indexes (e.g., tibia weight, tibia length, tibia diameter and tibia growth plate width) in the CON and TD groups were analyzed by Pearson correlation test and meanwhile visualized by using GraphPad Prism software. Receiver operating characteristic (ROC) curve analysis obtained (GraphPad Prism V.8) for the display of the constructed models, then the area under the ROC curve (AUC) was used to designate the ROC effect. *p* < 0.05 was considered to significant.

## Data Availability

The data presented in the study are deposited in the National Center for Biotechnology Information (NCBI) repository, accession number PRJNA755075.

## Abbreviations

AA, arbor acres; ALP, alkaline phosphatase; AUC, area under the ROC curve; Ca, calcium; ELISA, enzyme-linked immunosorbent assay; LDA, linear discriminant analysis; LEfSe, linear discriminant analysis; OPLS-DA, orthogonal partial least squares discriminant analysis; P, phosphorus; PCA, principal analysis; PLS-DA, partial least squares discriminant analysis; ROC, receiver operating characteristic; TD, tibial chondrodysplasia; TGP, tibia growth plate; TGPI, tibia growth plate index; TRACP, tartrate-resistant acid phosphatase; VIP, variable importance in project.

## References

[B1] Ahn T.-K. Kim J. O. An H. J. Park H. S. Choi U. Y. Sohn S. (2020). 3'-UTR Polymorphisms of Vitamin B-Related Genes Are Associated with Osteoporosis and Osteoporotic Vertebral Compression Fractures (OVCFs) in Postmenopausal Women. Genes 11, 612. 10.3390/genes11060612 PMC7349196 32498429

[B2] Almeida Paz I. Garcia R. Bernardi R. Nääs I. Caldara F. Freitas L. (2010). Selecting Appropriate Bedding to Reduce Locomotion Problems in Broilers. Rev. Bras. Cienc. Avic. 12, 189–195. 10.1590/S1516-635X2010000300008

[B3] Binkley N. (2012). Vitamin D and Osteoporosis-Related Fracture. Archives Biochem. Biophysics 523, 115–122. 10.1016/j.abb.2012.02.004 22349359

[B4] Biver E. Berenbaum F. Valdes A. M. Araujo de Carvalho I. Bindels L. B. Brandi M. L. (2019). Gut Microbiota and Osteoarthritis Management: An Expert Consensus of the European Society for Clinical and Economic Aspects of Osteoporosis, Osteoarthritis and Musculoskeletal Diseases (ESCEO). Ageing Res. Rev. 55, 100946. 10.1016/j.arr.2019.100946 31437484

[B5] Castaneda M. Strong J. M. Alabi D. A. Hernandez C. J. (2020). The Gut Microbiome and Bone Strength. Curr. Osteoporos. Rep. 18, 677–683. 10.1007/s11914-020-00627-x 33030683 PMC7736097

[B6] Chen Y.-C. Greenbaum J. Shen H. Deng H.-W. (2017). Association between Gut Microbiota and Bone Health: Potential Mechanisms and Prospective. J. Clin. Endocrinol. Metab. 102, 3635–3646. 10.1210/jc.2017-00513 28973392 PMC5630250

[B7] Chen F. Chen Z. Chen M. Chen G. Huang Q. Yang X. (2021). Reduced Stress-Associated FKBP5 DNA Methylation Together with Gut Microbiota Dysbiosis Is Linked with the Progression of Obese PCOS Patients. npj Biofilms Microbiomes 7, 60. 10.1038/s41522-021-00231-6 34267209 PMC8282850

[B8] Derakhshanfar A. Kheirandish R. Alidadi S. Bidadkosh A. (2013). Study of Long Effects of Administration of Aspirin (Acetylsalicylic Acid) on Bone in Broiler Chickens. Comp. Clin. Pathol. 22, 1201–1204. 10.1007/s00580-012-1550-2

[B9] Di Stefano M. Veneto G. Malservisi S. Corazza G. R. (2001). Small Intestine Bacterial Overgrowth and Metabolic Bone Disease. Dig. Dis. Sci. 46, 1077–1082. 10.1023/a:1010722314493 11341652

[B10] Edwards H. M. Veltmann J. R. (1983). The Role of Calcium and Phosphorus in the Etiology of Tibial Dyschondroplasia in Young Chicks. J. Nutr. 113, 1568–1575. 10.1093/jn/113.8.1568 6681256

[B11] Farquharson C. Jefferies D. (2000). Chondrocytes and Longitudinal Bone Growth: the Development of Tibial Dyschondroplasia. Poult. Sci. 79, 994–1004. 10.1093/PS/79.7.994 10901201

[B12] Fendukly F. Karlsson I. Hanson H. S. Kronvall G. Dornbusch K. (2003). Patterns of Mutations in Target Genes in Septicemia Isolates of *Escherichia coli* and *Klebsiella pneumoniae* with Resistance or Reduced Susceptibility to Ciprofloxacin. Apmis 111, 857–866. 10.1034/j.1600-0463.2003.1110904.x 14510643

[B13] Garneau J. E. Tremblay D. M. Moineau S. (2008). Characterization of 1706, a Virulent Phage from Lactococcus Lactis with Similarities to Prophages from Other Firmicutes. Virology 373, 298–309. 10.1016/j.virol.2007.12.002 18191977

[B14] Genin O. Hasdai A. Shinder D. Pines M. (2012). The Effect of Inhibition of Heat-Shock Proteins on Thiram-Induced Tibial Dyschondroplasia. Poult. Sci. 91, 1619–1626. 10.3382/ps.2012-02207 22700507

[B15] Groves P. J. Muir W. I. (2017). Earlier Hatching Time Predisposes Cobb Broiler Chickens to Tibial Dyschondroplasia. Animal 11, 112–120. 10.1017/S1751731116001105 27297908

[B16] Hao X. Shang X. Liu J. Chi R. Zhang J. Xu T. (2021). The Gut Microbiota in Osteoarthritis: where Do We Stand and what Can We Do? Arthritis Res. Ther. 23, 42. 10.1186/s13075-021-02427-9 33504365 PMC7839300

[B17] He J. Xu S. Zhang B. Xiao C. Chen Z. Si F. (2020). Gut Microbiota and Metabolite Alterations Associated with Reduced Bone Mineral Density or Bone Metabolic Indexes in Postmenopausal Osteoporosis. Aging 12, 8583–8604. 10.18632/aging.103168 32392181 PMC7244073

[B18] HolmesLi E. J. V. Li J. V. Marchesi J. R. Nicholson J. K. (2012). Gut Microbiota Composition and Activity in Relation to Host Metabolic Phenotype and Disease Risk. Cell Metab. 16, 559–564. 10.1016/j.cmet.2012.10.007 23140640

[B19] Huang S.-c. Rehman M. U. Lan Y.-f. Qiu G. Zhang H. Iqbal M. K. (2017). Tibial Dyschondroplasia Is Highly Associated with Suppression of Tibial Angiogenesis through Regulating the HIF-1α/VEGF/VEGFR Signaling Pathway in Chickens. Sci. Rep. 7, 9089. 10.1038/s41598-017-09664-6 28831181 PMC5567304

[B20] Huang S.-c. Zhang L.-h. Zhang J.-l. Rehman M. U. Tong X.-l. Qiu G. (2018). Role and Regulation of Growth Plate Vascularization during Coupling with Osteogenesis in Tibial Dyschondroplasia of Chickens. Sci. Rep. 8, 3680. 10.1038/s41598-018-22109-y 29487404 PMC5829164

[B21] Huang S. Kong A. Cao Q. Tong Z. Wang X. (2019). The Role of Blood Vessels in Broiler Chickens with Tibial Dyschondroplasia. Poult. Sci. 98, 6527–6532. 10.3382/ps/pez497 31433842 PMC8913930

[B22] Huang S.-c. Cao Q.-q. Cao Y.-b. Yang Y.-r. Xu T.-t. Yue K. (2021). Morinda Officinalis Polysaccharides Improve Meat Quality by Reducing Oxidative Damage in Chickens Suffering from Tibial Dyschondroplasia. Food Chem. 344, 128688. 10.1016/j.foodchem.2020.128688 33246686

[B23] Katono T. Kawato T. Tanabe N. Suzuki N. Iida T. Morozumi A. (2008). Sodium Butyrate Stimulates Mineralized Nodule Formation and Osteoprotegerin Expression by Human Osteoblasts. Archives Oral Biol. 53, 903–909. 10.1016/j.archoralbio.2008.02.016 18406397

[B24] Kim M. Vogtmann E. Ahlquist D. A. Devens M. E. Kisiel J. B. Taylor W. R. (2020). Fecal Metabolomic Signatures in Colorectal Adenoma Patients Are Associated with Gut Microbiota and Early Events of Colorectal Cancer Pathogenesis. Mbio 11, e3119–86. 10.1128/mBio.03186-19 PMC7029137 32071266

[B25] Klein M. S. Newell C. Bomhof M. R. Reimer R. A. Hittel D. S. Rho J. M. (2016). Metabolomic Modeling to Monitor Host Responsiveness to Gut Microbiota Manipulation in the BTBRT+tf/j Mouse. J. Proteome Res. 15, 1143–1150. 10.1021/acs.jproteome.5b01025 26928523

[B26] Kong A. Zhang C. Cao Y. Cao Q. Liu F. Yang Y. (2020). The Fungicide Thiram Perturbs Gut Microbiota Community and Causes Lipid Metabolism Disorder in Chickens. Ecotoxicol. Environ. Saf. 206, 111400. 10.1016/j.ecoenv.2020.111400 33010593

[B27] La Reau A. J. Suen G. (2018). The Ruminococci: Key Symbionts of the Gut Ecosystem. J. Microbiol. 56, 199–208. 10.1007/s12275-018-8024-4 29492877

[B28] Leach R. M. Monsonego-Ornan E. (2007). Tibial Dyschondroplasia 40 Years Later. Poult. Sci. 86, 2053–2058. 10.1093/PS/86.10.2053 17878435

[B29] Li C. Jiang Z. Liu X. (2010). Biochemical Mechanism of Gallium on Prevention of Fatal Cage-Layer Osteoporosis. Biol. Trace Elem. Res. 134, 195–202. 10.1007/s12011-009-8467-x 19639269

[B30] Ling C.-w. Miao Z. Xiao M.-l. Zhou H. Jiang Z. Fu Y. (2021). The Association of Gut Microbiota with Osteoporosis Is Mediated by Amino Acid Metabolism: Multiomics in a Large Cohort. J. Clin. Endocrinol. Metab. 106, e3852–e3864. 10.1210/clinem/dgab492 34214160

[B31] Liu Z. Li A. Wang Y. Iqbal M. Zheng A. Zhao M. (2020). Comparative Analysis of Microbial Community Structure between Healthy and Aeromonas Veronii-Infected Yangtze Finless Porpoise. Microb. Cell Fact. 19, 123. 10.1186/s12934-020-01383-4 32503532 PMC7275351

[B32] Lu L. Chen X. Liu Y. Yu X. (2021). Gut Microbiota and Bone Metabolism. FASEB J. 35, e21740. 10.1096/fj.202100451R 34143911

[B33] Lucas S. Omata Y. Hofmann J. Böttcher M. Iljazovic A. Sarter K. (2018). Short-chain Fatty Acids Regulate Systemic Bone Mass and Protect from Pathological Bone Loss. Nat. Commun. 9, 55. 10.1038/s41467-017-02490-4 29302038 PMC5754356

[B34] McKinney C. A. Oliveira B. C. M. Bedenice D. Paradis M.-R. Mazan M. Sage S. (2020). The Fecal Microbiota of Healthy Donor Horses and Geriatric Recipients Undergoing Fecal Microbial Transplantation for the Treatment of Diarrhea. Plos One 15, e0230148. 10.1371/journal.pone.0230148 32155205 PMC7064224

[B35] Meng X. Zhang G. Cao H. Yu D. Fang X. Vos W. M. (2020). Gut Dysbacteriosis and Intestinal Disease: Mechanism and Treatment. J. Appl. Microbiol. 129, 787–805. 10.1111/jam.14661 32277534 PMC11027427

[B36] Murga-Garrido S. M. Hong Q. Cross T.-W. L. Hutchison E. R. Han J. Thomas S. P. (2021). Gut Microbiome Variation Modulates the Effects of Dietary Fiber on Host Metabolism. Microbiome 9, 117. 10.1186/s40168-021-01061-6 34016169 PMC8138933

[B37] Pan R. Zhang X. Gao J. Yi W. Wei Q. Su H. (2020). Analysis of the Diversity of Intestinal Microbiome and its Potential Value as a Biomarker in Patients with Schizophrenia: A Cohort Study. Psychiatry Res. 291, 113260. 10.1016/j.psychres.2020.113260 32763534

[B38] Pelicia K. Aparecido Jr I. Garcia E. Molino A. Santos G. Berto D. (2012). Evaluation of a Radiographic Method to Detect Tibial Dyschondroplasia Lesions in Broilers. Rev. Bras. Cienc. Avic. 14, 129–135. 10.1590/S1516-635X2012000200007

[B39] Poulos P. W. (1978). Tibial Dyschondroplasia (Osteochondrosis) in the turkey. A Morphologic Investigation. Acta Radiol. Suppl. 358, 197–227. 233597

[B40] Rath N. C. Richards M. P. Huff W. E. Huff G. R. Balog J. M. (2005). Changes in the Tibial Growth Plates of Chickens with Thiram-Induced Dyschondroplasia. J. Comp. Pathology 133, 41–52. 10.1016/J.JCPA.2005.01.005 15899490

[B41] Rath N. C. Kannan L. Pillai P. B. Huff W. E. Huff G. R. Horst R. L. (2007). Evaluation of the Efficacy of Vitamin D3 or its Metabolites on Thiram-Induced Tibial Dyschondroplasia in Chickens. Res. Veterinary Sci. 83, 244–250. 10.1016/J.RVSC.2006.12.008 17307209

[B42] Rodriguez-R L. M. Castro J. C. Kyrpides N. C. Cole J. R. Tiedje J. M. Konstantinidis K. T. (2018). How Much Do rRNA Gene Surveys Underestimate Extant Bacterial Diversity? Appl. Environ. Microbiol. 84, e00014–18. 10.1128/AEM.00014-18 29305502 PMC5835724

[B43] Sánchez-Alcoholado L. Ordóñez R. Otero A. Plaza-Andrade I. Laborda-Illanes A. Medina J. A. (2020). Gut Microbiota-Mediated Inflammation and Gut Permeability in Patients with Obesity and Colorectal Cancer. Ijms 21, 6782. 10.3390/ijms21186782 PMC7555154 32947866

[B44] Schiering C. Wincent E. Metidji A. Iseppon A. Li Y. Potocnik A. J. (2017). Feedback Control of AHR Signalling Regulates Intestinal Immunity. Nature 542, 242–245. 10.1038/nature21080 28146477 PMC5302159

[B45] Schwartz M. (1978). [59] Thymidine Phosphorylase from *Escherichia coli* . Methods Enzymol. 51, 442–445. 10.1016/s0076-6879(78)51061-6 357904

[B46] Stentoft C. Røjen B. A. Jensen S. K. Kristensen N. B. Vestergaard M. Larsen M. (2015). Absorption and Intermediary Metabolism of Purines and Pyrimidines in Lactating Dairy Cows. Br. J. Nutr. 113, 560–573. 10.1017/S0007114514004000 25619278

[B47] Stewart C. J. Mansbach J. M. Wong M. C. Ajami N. J. Petrosino J. F. Camargo C. A. (2017). Associations of Nasopharyngeal Metabolome and Microbiome with Severity Among Infants with Bronchiolitis. A Multiomic Analysis. Am. J. Respir. Crit. Care Med. 196, 882–891. 10.1164/rccm.201701-0071OC 28530140 PMC5649976

[B48] Stotzer P. O. Johansson C. Mellström D. Lindstedt G. Kilander A. F. (2003). Bone Mineral Density in Patients with Small Intestinal Bacterial Overgrowth. Hepatogastroenterology 50, 1415–1418. 14571751

[B49] Tomasova L. Grman M. Ondrias K. Ufnal M. (2021). The Impact of Gut Microbiota Metabolites on Cellular Bioenergetics and Cardiometabolic Health. Nutr. Metab. (Lond) 18, 72. 10.1186/s12986-021-00598-5 34266472 PMC8281717

[B50] Tong X. Rehman M. U. Huang S. Jiang X. Zhang H. Li J. (2018). Comparative Analysis of Gut Microbial Community in Healthy and Tibial Dyschondroplasia Affected Chickens by High Throughput Sequencing. Microb. Pathog. 118, 133–139. 10.1016/j.micpath.2018.03.001 29555507

[B51] Tsoukalas D. Sarandi E. Georgaki S. (2021). The Snapshot of Metabolic Health in Evaluating Micronutrient Status, the Risk of Infection and Clinical Outcome of COVID-19. Clin. Nutr. ESPEN 44, 173–187. 10.1016/j.clnesp.2021.06.011 34330463 PMC8234252

[B52] Wang X. Ye T. Chen W.-J. Lv Y. Hao Z. Chen J. (2017). Structural Shift of Gut Microbiota during Chemo-Preventive Effects of Epigallocatechin Gallate on Colorectal Carcinogenesis in Mice. Wjg 23, 8128–8139. 10.3748/wjg.v23.i46.8128 29290650 PMC5739920

[B53] Wang Y. Li A. Zhang L. Waqas M. Mehmood K. Iqbal M. (2019). Probiotic Potential of Lactobacillus on the Intestinal Microflora against *Escherichia coli* Induced Mice Model through High-Throughput Sequencing. Microb. Pathog. 137, 103760. 10.1016/j.micpath.2019.103760 31562897

[B54] Wang X. Liu H. Li Y. Huang S. Zhang L. Cao C. (2020). Altered Gut Bacterial and Metabolic Signatures and Their Interaction in Gestational Diabetes Mellitus. Gut microbes 12, e1840765. 10.1080/19490976.2020.1840765 PMC7714515 33222612

[B55] Watson E. Olin-Sandoval V. Hoy M. J. Li C.-H. Louisse T. Yao V. (2016). Metabolic Network Rewiring of Propionate Flux Compensates Vitamin B12 Deficiency in *C. elegans* . Elife 5, e17670. 10.7554/eLife.17670 27383050 PMC4951191

[B56] Witkowski M. Weeks T. L. Hazen S. L. (2020). Gut Microbiota and Cardiovascular Disease. Circ. Res. 127, 553–570. 10.1161/CIRCRESAHA.120.316242 32762536 PMC7416843

[B57] Xiao H.-w. Cui M. Li Y. Dong J.-l. Zhang S.-q. Zhu C.-c. (2020). Gut Microbiota-Derived Indole 3-propionic Acid Protects against Radiation Toxicity via Retaining Acyl-CoA-Binding Protein. Microbiome 8, 69. 10.1186/s40168-020-00845-6 32434586 PMC7241002

[B58] Xu T. Yue K. Zhang C. Tong X. Lin L. Cao Q. (2021). Probiotics Treatment of Leg Diseases in Broiler Chickens: a Review. Probiotics Antimicro. Prot. 1–11. 10.1007/s12602-021-09869-2 34757604

[B59] Zaiss M. M. Jones R. M. Schett G. Pacifici R. (2019). The Gut-Bone axis: How Bacterial Metabolites Bridge the Distance. J. Clin. Investig. 129, 3018–3028. 10.1172/JCI128521 31305265 PMC6668676

[B60] Zhang L. Jiang X. Li A. Waqas M. Gao X. Li K. (2020). Characterization of the Microbial Community Structure in Intestinal Segments of Yak (Bos Grunniens). Anaerobe 61, 102115. 10.1016/j.anaerobe.2019.102115 31711887

[B61] Zheng P. Li Y. Wu J. Zhang H. Huang Y. Tan X. (2019). Perturbed Microbial Ecology in Myasthenia Gravis: Evidence from the Gut Microbiome and Fecal Metabolome. Adv. Sci. 6, 1901441. 10.1002/advs.201901441 PMC6755540 31559142

[B62] Zhu Q. Hou Q. Huang S. Ou Q. Huo D. Vázquez-Baeza Y. (2021). Compositional and Genetic Alterations in Graves' Disease Gut Microbiome Reveal Specific Diagnostic Biomarkers. Isme J. 15, 3399–3411. 10.1038/s41396-021-01016-7 34079079 PMC8528855

[B63] Zimmermann J. Obeng N. Yang W. Pees B. Petersen C. Waschina S. (2019). The Functional Repertoire Contained within the Native Microbiota of the Model Nematode *Caenorhabditis elegans* . Isme J. 14, 26–38. 10.1038/s41396-019-0504-y 31484996 PMC6908608

[B64] Zuccarini M. Giuliani P. Caciagli F. Ciccarelli R. Di Iorio P. (2021). In Search of a Role for Extracellular Purine Enzymes in Bone Function. Biomolecules 11, 679. 10.3390/biom11050679 33946568 PMC8147220

