# Supplementary material for: Integrated Fecal Microbiome and Metabolomics Reveals a Novel Potential Biomarker for Predicting Tibial Dyschondroplasia in Chickens
Source: Front Physiol. 2022 May 12;13:887207. doi: 10.3389/fphys.2022.887207 (PMC9133743; doi:10.3389/fphys.2022.887207)
Supplement: Supplementary file 1 [file DataSheet1.PDF]

Integrated fecal microbiome and metabolomics reveals a novel potential biomarker for predicting tibial chondrodysplasia in chickens

Shucheng Huang[a,*], Caodong Zhang[a], Tingting Xu[a], Aftab Shaukat[b], Yanfeng He[a], Pan Chen[a], Luxi Lin[a], Ke Yue[a], Qinqin Cao[a], Xishuai Tong[c]

Supplementary material

Figure: 2

Files: 5

Words: 8895

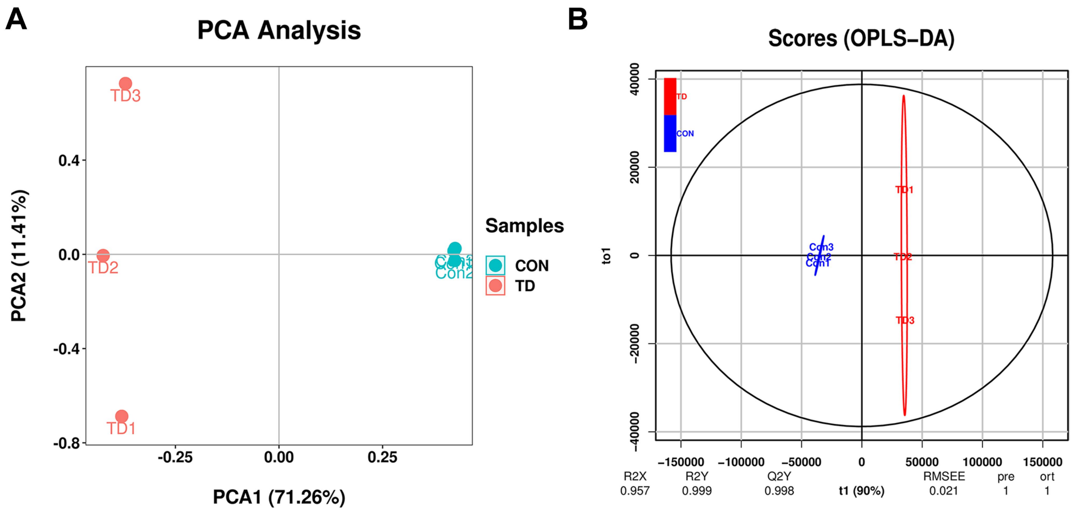

Fig. S1. Fecal metabolomic signatures of broiler chickens.

(**A**) PCA estimates for broiler fecal metabolites of the CON group (red) and TD group (green). (**B**) OPLS-DA analysis of fecal metabolites in the CON group (blue) and TD group (red) of broiler chickens.

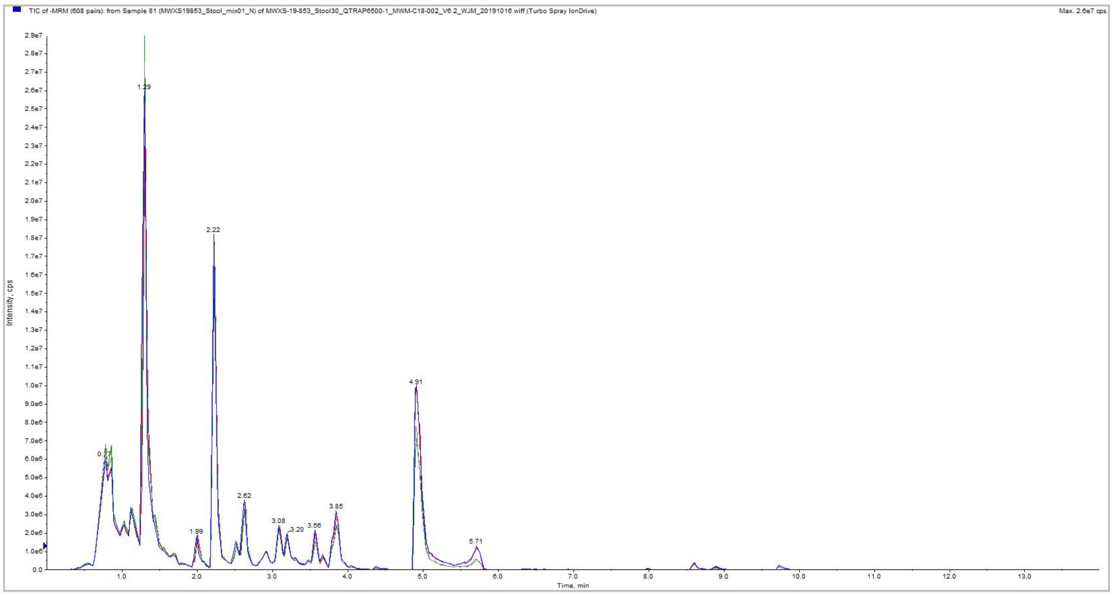

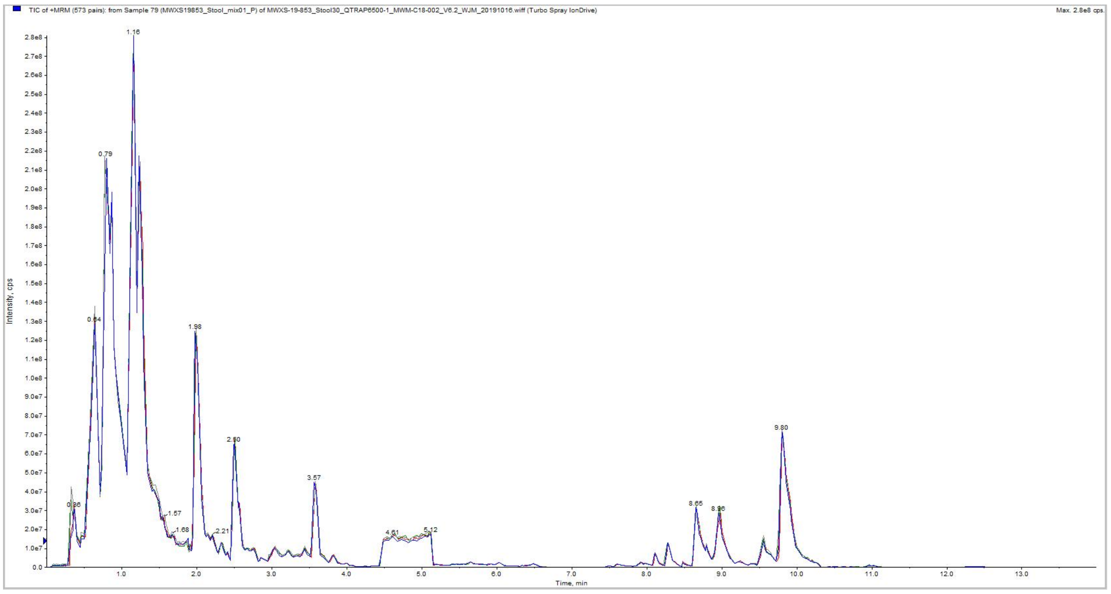

Fig. S2. TIC plot for QC sample mass spectrometry

File S1. Relative abundance of microbiota at the phylum level

Taxon	CON	TD
k__Bacteria;p__Actinobacteria	80.25	288
k__Bacteria;p__Armatimonadetes	0	0.25
k__Bacteria;p__Bacteroidetes	73	115.5
k__Bacteria;p__Chloroflexi	0.5	0.5
k__Bacteria;p__Cyanobacteria	13.5	8.5
k__Bacteria;p__Firmicutes	39203	37491.25
k__Bacteria;p__Fusobacteria	1.25	1.25
k__Bacteria;p__Nitrospirae	0	0.5
k__Bacteria;p__Planctomycetes	6.25	2.25
k__Bacteria;p__Proteobacteria	1152.25	2895.5
k__Bacteria;p__Tenericutes	370	52.5
k__Bacteria;p__Verrucomicrobia	0.75	2
k__Bacteria;p__[Thermi]	12.25	17.25

File S2. Relative abundance of microbiota at the genus level.

Taxon	CON	TD
k__Bacteria;p__Actinobacteria;c__Actinobacteria;o__Actinomycetales;f__Actinomycetaceae;g__Actinomyces	0	1
k__Bacteria;p__Actinobacteria;c__Actinobacteria;o__Actinomycetales;f__Brevibacteriaceae;g__Brevibacterium	2	0
k__Bacteria;p__Actinobacteria;c__Actinobacteria;o__Actinomycetales;f__Corynebacteriaceae;g__Corynebacterium	9.5	238.75
k__Bacteria;p__Actinobacteria;c__Actinobacteria;o__Actinomycetales;f__Dermabacteraceae;g__Brachybacterium	0	1.25
k__Bacteria;p__Actinobacteria;c__Actinobacteria;o__Actinomycetales;f__Intrasporangiaceae;g__Phycicoccus	0.25	0
k__Bacteria;p__Actinobacteria;c__Actinobacteria;o__Actinomycetales;f__Microbacteriaceae;g__Microbacterium	2.75	0.75
k__Bacteria;p__Actinobacteria;c__Actinobacteria;o__Actinomycetales;f__Micrococcaceae;g__Arthrobacter	0	10.25
k__Bacteria;p__Actinobacteria;c__Actinobacteria;o__Actinomycetales;f__Micrococcaceae;g__Rothia	0	3.75
k__Bacteria;p__Actinobacteria;c__Actinobacteria;o__Actinomycetales;f__Nocardiaceae;g__Rhodococcus	4.5	1.75
k__Bacteria;p__Actinobacteria;c__Actinobacteria;o__Actinomycetales;f__Nocardioidaceae;g__Unclassified_Nocardioidaceae	0.25	0.5
k__Bacteria;p__Actinobacteria;c__Actinobacteria;o__Actinomycetales;f__Propionibacteriaceae;g__Propionibacterium	2.25	2.75
k__Bacteria;p__Actinobacteria;c__Actinobacteria;o__Actinomycetales;f__Pseudonocardiaceae;g__Amycolatopsis	4	2.75
k__Bacteria;p__Actinobacteria;c__Actinobacteria;o__Actinomycetales;f__Streptomycetaceae;g__Unclassified_Streptomycetaceae	0.25	0
k__Bacteria;p__Actinobacteria;c__Actinobacteria;o__Bifidobacteriales;f__Bifidobacteriaceae;g__Bifidobacterium	0.5	13.25
k__Bacteria;p__Actinobacteria;c__Coriobacteriia;o__Coriobacteriales;f__Coriobacteriaceae;g__Collinsella	0	4.5
k__Bacteria;p__Actinobacteria;c__Coriobacteriia;o__Coriobacteriales;f__Coriobacteriaceae;g__Eggerthella	51.5	5.5
k__Bacteria;p__Actinobacteria;c__Coriobacteriia;o__Coriobacteriales;f__Coriobacteriaceae;g__Unclassified_Coriobacteriaceae	2.5	1.25
k__Bacteria;p__Armatimonadetes;c__[Fimbriimonadia];o__[Fimbriimonadales];f__[Fimbriimonadaceae];g__Fimbriimonas	0	0.25
k__Bacteria;p__Bacteroidetes;c__Bacteroidia;o__Bacteroidales;f__Bacteroidaceae;g__Bacteroides	0	5.5
k__Bacteria;p__Bacteroidetes;c__Bacteroidia;o__Bacteroidales;f__Porphyromonadaceae;g__Parabacteroides	0	4.5

k__Bacteria;p__Bacteroidetes;c__Bacteroidia;o__Bacteroidales;f__S24-7;g__Unclassified_S24-7	0.75	11
k__Bacteria;p__Bacteroidetes;c__Flavobacteriia;o__Flavobacteriales;f__Flavobacteriaceae;g__Myroides	0	15.75
k__Bacteria;p__Bacteroidetes;c__Flavobacteriia;o__Flavobacteriales;f__[Weeksellaceae];g__Elizabethkingia	0.25	1.25
k__Bacteria;p__Bacteroidetes;c__Flavobacteriia;o__Flavobacteriales;f__[Weeksellaceae];g__Wautersiella	0	9.75
k__Bacteria;p__Bacteroidetes;c__Sphingobacteriia;o__Sphingobacteriales;f__Sphingobacteriaceae;g__Sphingobacterium	0	43.25
k__Bacteria;p__Bacteroidetes;c__[Saprospirae];o__[Saprospirales];f__Chitinophagaceae;g__Sediminibacterium	72	24.5
k__Bacteria;p__Chloroflexi;c__Anaerolineae;o__SBR1031;f__A4b;g__Unclassified_A4b	0.5	0.5
k__Bacteria;p__Cyanobacteria;c__4C0d-2;o__MLE1-12;f__Unclassified_MLE1-12;g__Unclassified_MLE1-12	13	5
k__Bacteria;p__Cyanobacteria;c__Chloroplast;o__Streptophyta;f__Unclassified_Streptophyta;g__Unclassified_Streptophyta	0	3.5
k__Bacteria;p__Cyanobacteria;c__ML635J-21;o__Unclassified_ML635J-21;f__Unclassified_ML635J-21;g__Unclassified_ML635J-21	0.5	0
k__Bacteria;p__Firmicutes;c__Bacilli;o__Bacillales;f__Bacillaceae;g__Anoxybacillus	2.5	0.25
k__Bacteria;p__Firmicutes;c__Bacilli;o__Bacillales;f__Bacillaceae;g__Bacillus	2.75	148.25
k__Bacteria;p__Firmicutes;c__Bacilli;o__Bacillales;f__Bacillaceae;g__Natronobacillus	0.75	2.5
k__Bacteria;p__Firmicutes;c__Bacilli;o__Bacillales;f__Bacillaceae;g__Oceanobacillus	0	16
k__Bacteria;p__Firmicutes;c__Bacilli;o__Bacillales;f__Bacillaceae;g__Unclassified_Bacillaceae	22	6.25
k__Bacteria;p__Firmicutes;c__Bacilli;o__Bacillales;f__Bacillaceae;g__Virgibacillus	0.75	20
k__Bacteria;p__Firmicutes;c__Bacilli;o__Bacillales;f__Paenibacillaceae;g__Paenibacillus	0	2.75
k__Bacteria;p__Firmicutes;c__Bacilli;o__Bacillales;f__Planococcaceae;g__Kurthia	223.75	173
k__Bacteria;p__Firmicutes;c__Bacilli;o__Bacillales;f__Planococcaceae;g__Lysinibacillus	0	55
k__Bacteria;p__Firmicutes;c__Bacilli;o__Bacillales;f__Planococcaceae;g__Sporosarcina	0.25	198.5
k__Bacteria;p__Firmicutes;c__Bacilli;o__Bacillales;f__Planococcaceae;g__Unclassified_Planococcaceae	2	13.5
k__Bacteria;p__Firmicutes;c__Bacilli;o__Bacillales;f__Staphylococcaceae;g__Jeotgalicoccus	0.75	5.75
k__Bacteria;p__Firmicutes;c__Bacilli;o__Bacillales;f__Staphylococcaceae;g__Staphylococcus	0	1.5
k__Bacteria;p__Firmicutes;c__Bacilli;o__Bacillales;f__Staphylococcaceae;g__Unclassified_Staphylococcaceae	0	15

k__Bacteria;p__Firmicutes;c__Bacilli;o__Bacillales;f__Unclassified_Bacillales;g__Unclassified_Bacillales	1.5	155.25
k__Bacteria;p__Firmicutes;c__Bacilli;o__Lactobacillales;f__Aerococcaceae;g__Aerococcus	121.75	109.25
k__Bacteria;p__Firmicutes;c__Bacilli;o__Lactobacillales;f__Aerococcaceae;g__Facklamia	3.75	96
k__Bacteria;p__Firmicutes;c__Bacilli;o__Lactobacillales;f__Carnobacteriaceae;g__Unclassified_Carnobacteriaceae	0	1.75
k__Bacteria;p__Firmicutes;c__Bacilli;o__Lactobacillales;f__Enterococcaceae;g__Enterococcus	50.75	160.75
k__Bacteria;p__Firmicutes;c__Bacilli;o__Lactobacillales;f__Lactobacillaceae;g__Lactobacillus	25482.5	33472
k__Bacteria;p__Firmicutes;c__Bacilli;o__Lactobacillales;f__Lactobacillaceae;g__Pediococcus	0	1.25
k__Bacteria;p__Firmicutes;c__Bacilli;o__Lactobacillales;f__Lactobacillaceae;g__Unclassified_Lactobacillaceae	0	3.25
k__Bacteria;p__Firmicutes;c__Bacilli;o__Lactobacillales;f__Leuconostocaceae;g__Unclassified_Leuconostocaceae	0	54.25
k__Bacteria;p__Firmicutes;c__Bacilli;o__Lactobacillales;f__Leuconostocaceae;g__Weissella	33.25	38.75
k__Bacteria;p__Firmicutes;c__Bacilli;o__Lactobacillales;f__Streptococcaceae;g__Streptococcus	0.5	0.5
k__Bacteria;p__Firmicutes;c__Bacilli;o__Lactobacillales;f__Streptococcaceae;g__Unclassified_Streptococcaceae	1	0
k__Bacteria;p__Firmicutes;c__Bacilli;o__Lactobacillales;f__Unclassified_Lactobacillales;g__Unclassified_Lactobacillales	2.25	22.25
k__Bacteria;p__Firmicutes;c__Clostridia;o__Clostridiales;f__Christensenellaceae;g__Unclassified_Christensenellaceae	29.75	6
k__Bacteria;p__Firmicutes;c__Clostridia;o__Clostridiales;f__Clostridiaceae;g__Candidatus_Arthromitus	860	789
k__Bacteria;p__Firmicutes;c__Clostridia;o__Clostridiales;f__Clostridiaceae;g__Clostridium	9.25	2.5
k__Bacteria;p__Firmicutes;c__Clostridia;o__Clostridiales;f__Clostridiaceae;g__SMB53	0.25	0
k__Bacteria;p__Firmicutes;c__Clostridia;o__Clostridiales;f__Clostridiaceae;g__Unclassified_Clostridiaceae	27.5	1.25
k__Bacteria;p__Firmicutes;c__Clostridia;o__Clostridiales;f__Dehalobacteriaceae;g__Dehalobacterium	2.25	0
k__Bacteria;p__Firmicutes;c__Clostridia;o__Clostridiales;f__Dehalobacteriaceae;g__Unclassified_Dehalobacteriaceae	1.75	0
k__Bacteria;p__Firmicutes;c__Clostridia;o__Clostridiales;f__Eubacteriaceae;g__Anaerofustis	18	3.5
k__Bacteria;p__Firmicutes;c__Clostridia;o__Clostridiales;f__Lachnospiraceae;g__Anaerostipes	25.5	1.5
k__Bacteria;p__Firmicutes;c__Clostridia;o__Clostridiales;f__Lachnospiraceae;g__Blautia	545.25	70

k__Bacteria;p__Firmicutes;c__Clostridia;o__Clostridiales;f__Lachnospiraceae;g__Coprococcus	147.25	7.75
k__Bacteria;p__Firmicutes;c__Clostridia;o__Clostridiales;f__Lachnospiraceae;g__Dorea	452.25	31
k__Bacteria;p__Firmicutes;c__Clostridia;o__Clostridiales;f__Lachnospiraceae;g__Unclassified_Lachnospiraceae	2502.75	249.5
k__Bacteria;p__Firmicutes;c__Clostridia;o__Clostridiales;f__Lachnospiraceae;g__[Ruminococcus]	798	116.25
k__Bacteria;p__Firmicutes;c__Clostridia;o__Clostridiales;f__Peptostreptococcaceae;g__Unclassified_Peptostreptococcaceae	11.75	2
k__Bacteria;p__Firmicutes;c__Clostridia;o__Clostridiales;f__Ruminococcaceae;g__Anaerotruncus	13.25	1
k__Bacteria;p__Firmicutes;c__Clostridia;o__Clostridiales;f__Ruminococcaceae;g__Butyricicoccus	15.25	0.5
k__Bacteria;p__Firmicutes;c__Clostridia;o__Clostridiales;f__Ruminococcaceae;g__Clostridium	1	0
k__Bacteria;p__Firmicutes;c__Clostridia;o__Clostridiales;f__Ruminococcaceae;g__Faecalibacterium	0.75	1.5
k__Bacteria;p__Firmicutes;c__Clostridia;o__Clostridiales;f__Ruminococcaceae;g__Oscillospira	499.5	47.75
k__Bacteria;p__Firmicutes;c__Clostridia;o__Clostridiales;f__Ruminococcaceae;g__Ruminococcus	646.25	110.5
k__Bacteria;p__Firmicutes;c__Clostridia;o__Clostridiales;f__Ruminococcaceae;g__Unclassified_Ruminococcaceae	2278.75	411.25
k__Bacteria;p__Firmicutes;c__Clostridia;o__Clostridiales;f__Unclassified_Clostridiales;g__Unclassified_Clostridiales	3882.75	645.5
k__Bacteria;p__Firmicutes;c__Clostridia;o__Clostridiales;f__Veillonellaceae;g__Megasphaera	0	7
k__Bacteria;p__Firmicutes;c__Clostridia;o__Clostridiales;f__Veillonellaceae;g__Veillonella	0.5	0
k__Bacteria;p__Firmicutes;c__Clostridia;o__Clostridiales;f__[Mogibacteriaceae];g__Unclassified_[Mogibacteriaceae]	13	0
k__Bacteria;p__Firmicutes;c__Clostridia;o__Unclassified_Clostridia;f__Unclassified_Clostridia;g__Unclassified_Clostridia	0.5	0
k__Bacteria;p__Firmicutes;c__Erysipelotrichi;o__Erysipelotrichales;f__Erysipelotrichaceae;g__Coprobacillus	77.5	37.5
k__Bacteria;p__Firmicutes;c__Erysipelotrichi;o__Erysipelotrichales;f__Erysipelotrichaceae;g__Unclassified_Erysipelotrichaceae	357.25	173.75
k__Bacteria;p__Firmicutes;c__Erysipelotrichi;o__Erysipelotrichales;f__Erysipelotrichaceae;g__[Eubacterium]	21.25	0

k__Bacteria;p__Firmicutes;c__Erysipelotrichi;o__Erysipelotrichales;f__Erysipelotrichaceae;g__cc_115	11.25	1.25
k__Bacteria;p__Fusobacteria;c__Fusobacteriia;o__Fusobacteriales;f__Fusobacteriaceae;g__Fusobacterium	1.25	0.75
k__Bacteria;p__Fusobacteria;c__Fusobacteriia;o__Fusobacteriales;f__Leptotrichiaceae;g__Leptotrichia	0	0.5
k__Bacteria;p__Nitrospirae;c__Nitrospira;o__Nitrospirales;f__0319-6A21;g__Unclassified_0319-6A21	0	0.5
k__Bacteria;p__Planctomycetes;c__Phycisphaerae;o__Phycisphaerales;f__Unclassified_Phycisphaerales;g__Unclassified_Phycisphaerales	6.25	2.25
k__Bacteria;p__Proteobacteria;c__Alphaproteobacteria;o__Caulobacterales;f__Caulobacteraceae;g__Brevundimonas	2	37.75
k__Bacteria;p__Proteobacteria;c__Alphaproteobacteria;o__Caulobacterales;f__Caulobacteraceae;g__Unclassified_Caulobacteraceae	13	2.5
k__Bacteria;p__Proteobacteria;c__Alphaproteobacteria;o__Rhizobiales;f__Bradyrhizobiaceae;g__Bradyrhizobium	0	0.25
k__Bacteria;p__Proteobacteria;c__Alphaproteobacteria;o__Rhizobiales;f__Bradyrhizobiaceae;g__Unclassified_Bradyrhizobiaceae	25.75	18
k__Bacteria;p__Proteobacteria;c__Alphaproteobacteria;o__Rhizobiales;f__Brucellaceae;g__Ochrobactrum	274.5	139.75
k__Bacteria;p__Proteobacteria;c__Alphaproteobacteria;o__Rhizobiales;f__Brucellaceae;g__Pseudochrobactrum	1	1.75
k__Bacteria;p__Proteobacteria;c__Alphaproteobacteria;o__Rhizobiales;f__Hyphomicrobiaceae;g__Devosia	0.75	2.25
k__Bacteria;p__Proteobacteria;c__Alphaproteobacteria;o__Rhizobiales;f__Hyphomicrobiaceae;g__Rhodoplanes	2.5	1.25
k__Bacteria;p__Proteobacteria;c__Alphaproteobacteria;o__Rhizobiales;f__Methylobacteriaceae;g__Methylobacterium	18.5	13.75
k__Bacteria;p__Proteobacteria;c__Alphaproteobacteria;o__Rhizobiales;f__Methylobacteriaceae;g__Unclassified_Methylobacteriaceae	1	1
k__Bacteria;p__Proteobacteria;c__Alphaproteobacteria;o__Rhizobiales;f__Phyllobacteriaceae;g__Aminobacter	6	3
k__Bacteria;p__Proteobacteria;c__Alphaproteobacteria;o__Rhizobiales;f__Phyllobacteriaceae;g__Phyllobacterium	4.25	5
k__Bacteria;p__Proteobacteria;c__Alphaproteobacteria;o__Rhizobiales;f__Phyllobacteriaceae;g__Unclassified_Phyllobacteriaceae	6	1
k__Bacteria;p__Proteobacteria;c__Alphaproteobacteria;o__Rhizobiales;f__Rhizobiaceae;g__Agrobacterium	86.25	42
k__Bacteria;p__Proteobacteria;c__Alphaproteobacteria;o__Rhizobiales;f__Unclassified_Rhizobiales;g__Unclassified_Rhizobiales	1.75	0.5

k__Bacteria;p__Proteobacteria;c__Alphaproteobacteria;o__Rhodospirillales;f__Acetobacteraceae;g__Acetobacter	0.75	2.5
k__Bacteria;p__Proteobacteria;c__Alphaproteobacteria;o__Rhodospirillales;f__Rhodospirillaceae;g__Unclassified_Rhodospirillaceae	0.25	0
k__Bacteria;p__Proteobacteria;c__Alphaproteobacteria;o__Sphingomonadales;f__Erythrobacteraceae;g__Erythrobacter	2.5	1
k__Bacteria;p__Proteobacteria;c__Alphaproteobacteria;o__Sphingomonadales;f__Sphingomonadaceae;g__Sphingomonas	9.5	2.75
k__Bacteria;p__Proteobacteria;c__Alphaproteobacteria;o__Sphingomonadales;f__Sphingomonadaceae;g__Unclassified_Sphingomonadaceae	0	0.25
k__Bacteria;p__Proteobacteria;c__Betaproteobacteria;o__Burkholderiales;f__Alcaligenaceae;g__Sutterella	0	3.25
k__Bacteria;p__Proteobacteria;c__Betaproteobacteria;o__Burkholderiales;f__Comamonadaceae;g__Comamonas	2.75	2.5
k__Bacteria;p__Proteobacteria;c__Betaproteobacteria;o__Burkholderiales;f__Comamonadaceae;g__Delftia	6.5	6.25
k__Bacteria;p__Proteobacteria;c__Betaproteobacteria;o__Burkholderiales;f__Comamonadaceae;g__Methylibium	0.25	0
k__Bacteria;p__Proteobacteria;c__Betaproteobacteria;o__Burkholderiales;f__Comamonadaceae;g__Unclassified_Comamonadaceae	43.25	30.25
k__Bacteria;p__Proteobacteria;c__Betaproteobacteria;o__Burkholderiales;f__Oxalobacteraceae;g__Cupriavidus	0.5	1.5
k__Bacteria;p__Proteobacteria;c__Betaproteobacteria;o__Burkholderiales;f__Oxalobacteraceae;g__Ralstonia	0	0.75
k__Bacteria;p__Proteobacteria;c__Betaproteobacteria;o__Burkholderiales;f__Unclassified_Burkholderiales;g__Unclassified_Burkholderiales	0.75	1.5
k__Bacteria;p__Proteobacteria;c__Betaproteobacteria;o__MND1;f__Unclassified_MND1;g__Unclassified_MND1	0	1
k__Bacteria;p__Proteobacteria;c__Deltaproteobacteria;o__MIZ46;f__Unclassified_MIZ46;g__Unclassified_MIZ46	0.5	0.5
k__Bacteria;p__Proteobacteria;c__Deltaproteobacteria;o__Myxococcales;f__0319-6G20;g__Unclassified_0319-6G20	1	0.75
k__Bacteria;p__Proteobacteria;c__Deltaproteobacteria;o__Myxococcales;f__Cystobacterineae;g__Unclassified_Cystobacterineae	3.25	0.5
k__Bacteria;p__Proteobacteria;c__Deltaproteobacteria;o__Myxococcales;f__Myxococcaceae;g__Unclassified_Myxococcaceae	1	0
k__Bacteria;p__Proteobacteria;c__Deltaproteobacteria;o__Myxococcales;f__Unclassified_Myxococcales;g__Unclassified_Myxococcales	0.75	0

k__Bacteria;p__Proteobacteria;c__Gammaproteobacteria;o__Aeromonadales;f__Aeromonadaceae;g__Unclassified_Aeromonadaceae	0.5	5.5
k__Bacteria;p__Proteobacteria;c__Gammaproteobacteria;o__Alteromonadales;f__Shewanellaceae;g__Shewanella	0	0.25
k__Bacteria;p__Proteobacteria;c__Gammaproteobacteria;o__Alteromonadales;f__[Chromatiaceae];g__Unclassified_[Chromatiaceae]	0	1.5
k__Bacteria;p__Proteobacteria;c__Gammaproteobacteria;o__Enterobacteriales;f__Enterobacteriaceae;g__Citrobacter	0	2.25
k__Bacteria;p__Proteobacteria;c__Gammaproteobacteria;o__Enterobacteriales;f__Enterobacteriaceae;g__Enterobacter	0	1.75
k__Bacteria;p__Proteobacteria;c__Gammaproteobacteria;o__Enterobacteriales;f__Enterobacteriaceae;g__Erwinia	0.25	1.75
k__Bacteria;p__Proteobacteria;c__Gammaproteobacteria;o__Enterobacteriales;f__Enterobacteriaceae;g__Klebsiella	10.25	77.5
k__Bacteria;p__Proteobacteria;c__Gammaproteobacteria;o__Enterobacteriales;f__Enterobacteriaceae;g__Providencia	2.5	1034.5
k__Bacteria;p__Proteobacteria;c__Gammaproteobacteria;o__Enterobacteriales;f__Enterobacteriaceae;g__Serratia	0	8
k__Bacteria;p__Proteobacteria;c__Gammaproteobacteria;o__Enterobacteriales;f__Enterobacteriaceae;g__Unclassified_Enterobacteriaceae	543.5	926.25
k__Bacteria;p__Proteobacteria;c__Gammaproteobacteria;o__Pasteurellales;f__Pasteurellaceae;g__Haemophilus	0.75	0
k__Bacteria;p__Proteobacteria;c__Gammaproteobacteria;o__Pseudomonadales;f__Moraxellaceae;g__Acinetobacter	67.5	497.25
k__Bacteria;p__Proteobacteria;c__Gammaproteobacteria;o__Pseudomonadales;f__Moraxellaceae;g__Enhydrobacter	0.75	0
k__Bacteria;p__Proteobacteria;c__Gammaproteobacteria;o__Pseudomonadales;f__Moraxellaceae;g__Unclassified_Moraxellaceae	0.75	9.25
k__Bacteria;p__Proteobacteria;c__Gammaproteobacteria;o__Pseudomonadales;f__Pseudomonadaceae;g__Pseudomonas	4.5	2.75
k__Bacteria;p__Proteobacteria;c__Gammaproteobacteria;o__Vibrionales;f__Vibrionaceae;g__Vibrio	1.25	0
k__Bacteria;p__Proteobacteria;c__Gammaproteobacteria;o__Xanthomonadales;f__Sinobacteraceae;g__Unclassified_Sinobacteraceae	0.5	0
k__Bacteria;p__Proteobacteria;c__Gammaproteobacteria;o__Xanthomonadales;f__Xanthomonadaceae;g__Unclassified_Xanthomonadaceae	2.5	2
k__Bacteria;p__Tenericutes;c__Mollicutes;o__Anaeroplasmatales;f__Anaeroplasmataceae;g__Anaeroplasma	0.75	0
k__Bacteria;p__Tenericutes;c__Mollicutes;o__RF39;f__Unclassified_RF39;g__Unclassified_RF39	369.25	52.5

k__Bacteria;p__Verrucomicrobia;c__Verrucomicrobiae;o__Verrucomicrobiales;f__Verrucomicrobiaceae;g__Akkermansia	0.75	2
k__Bacteria;p__[Thermi];c__Deinococci;o__Deinococcales;f__Deinococcaceae;g__Deinococcus	0.25	0.25
k__Bacteria;p__[Thermi];c__Deinococci;o__Thermales;f__Thermaceae;g__Thermus	12	17

File S3. LEfSe comparison analysis between the CON and TD groups

Taxon	Relative abundance	Group	LDA score	P-value
Bacteria.Firmicutes.Bacilli	5.929761101	TD	5.081616933	0.043308143
Bacteria.Proteobacteria.Gammaproteobacteria	4.798737116	TD	4.324338029	0.020921335
Bacteria.Proteobacteria.Gammaproteobacteria.Enterobacteriales.Enterobacteriaceae	4.700896298	TD	4.278362906	0.020921335
Bacteria.Proteobacteria.Gammaproteobacteria.Enterobacteriales	4.700896298	TD	4.255256332	0.020921335
Bacteria.Proteobacteria.Gammaproteobacteria.Enterobacteriales.Enterobacteriaceae.Klebsiella	3.27812277	TD	2.929290014	0.043308143
Bacteria.Actinobacteria.Actinobacteria.Actinomycetales.Micrococcaceae.Arthrobacter	2.399448546	TD	2.860215159	0.047220904
Bacteria.Firmicutes.Bacilli.Bacillales.Planococcaceae.Lysinibacillus	3.129150602	TD	2.853765628	0.047220904
Bacteria.Proteobacteria.Gammaproteobacteria.Enterobacteriales.Enterobacteriaceae.Citrobacter	1.740659991	TD	2.830507172	0.047220904
Bacteria.Bacteroidetes.Sphingobacteriia	3.024708198	TD	2.773135626	0.047220904
Bacteria.Bacteroidetes.Sphingobacteriia.Sphingobacteriales	3.024708198	TD	2.764813641	0.047220904
Bacteria.Bacteroidetes.Sphingobacteriia.Sphingobacteriales.Sphingobacteriaceae.Sphingobacterium	3.024708198	TD	2.756913421	0.047220904

Bacteria.Bacteroidetes.Sphingobacteriia.Sphingobacteriales.Sphingobacteriaceae	3.024708198	TD	2.742719674	0.047220904
Bacteria.Bacteroidetes.Flavobacteriia.Flavobacteriales.Flavobacteriaceae.Myroides	2.586080946	TD	2.557541554	0.047220904
Bacteria.Actinobacteria.Actinobacteria.Actinomycetales.Micrococcaceae	2.534838004	TD	2.543376803	0.047220904
Bacteria.Firmicutes.Bacilli.Bacillales.Bacillaceae.Oceanobacillus	2.592881112	TD	2.529610078	0.047220904
Bacteria.Bacteroidetes.Flavobacteriia.Flavobacteriales._Weeksellaceae_.Wautersiella	2.377763961	TD	2.509091527	0.047220904
Bacteria.Bacteroidetes.Flavobacteriia.Flavobacteriales.Flavobacteriaceae	2.586080946	TD	2.49676467	0.047220904
Bacteria.Proteobacteria.Gammaproteobacteria.Enterobacteriales.Enterobacteriaceae.Serratia	2.291853687	TD	2.423154113	0.047220904
Bacteria.Firmicutes.Clostridia	5.494741256	CON	5.105008057	0.020921335
Bacteria.Firmicutes.Clostridia.Clostridiales	5.494724261	CON	5.097540012	0.020921335
Bacteria.Firmicutes.Clostridia.Clostridiales.Lachnospiraceae	5.038504162	CON	4.670301838	0.043308143
Bacteria.Firmicutes.Clostridia.Clostridiales.Ruminococcaceae	4.926517396	CON	4.522171319	0.043308143
Bacteria.Firmicutes.Clostridia.Clostridiales.Lachnospiraceae._Ruminococcus_	4.290114025	CON	3.899849605	0.043308143
Bacteria.Firmicutes.Clostridia.Clostridiales.Ruminococcaceae.Ruminococcus	4.198505001	CON	3.794750443	0.043308143

Bacteria.Firmicutes.Clostridia.Clostridiales.Ruminococcaceae.Oscillospira	4.086629006	CON	3.69088814	0.043308143
Bacteria.Firmicutes.Clostridia.Clostridiales.Lachnospiraceae.Dorea	4.043473994	CON	3.675080698	0.043308143
Bacteria.Tenericutes.Mollicutes.RF39	3.955423295	CON	3.570043544	0.020921335
Bacteria.Tenericutes	3.956304459	CON	3.567432164	0.020921335
Bacteria.Tenericutes.Mollicutes	3.956304459	CON	3.566563421	0.020921335
Bacteria.Proteobacteria.Alphaproteobacteria.Rhizobiales	4.019847328	CON	3.421951016	0.043308143
Bacteria.Proteobacteria.Deltaproteobacteria.Myxococcales.Cystobacterineae	1.900016355	CON	3.417188355	0.017960478
Bacteria.Proteobacteria.Alphaproteobacteria.Rhizobiales.Brucellaceae.Ochrobactrum	3.826694012	CON	3.246941391	0.043308143
Bacteria.Firmicutes.Clostridia.Clostridiales.Lachnospiraceae.Coprococcus	3.556156946	CON	3.243592116	0.043308143
Bacteria.Actinobacteria.Actinobacteria.Actinomycetales.Brevibacteriaceae	1.689184641	CON	3.237391605	0.047220904
Bacteria.Proteobacteria.Alphaproteobacteria.Rhizobiales.Brucellaceae	3.828272603	CON	3.217963663	0.043308143
Bacteria.Actinobacteria.Actinobacteria.Actinomycetales.Brevibacteriaceae.Brevibacterium	1.689184641	CON	3.207075046	0.047220904
Bacteria.Firmicutes.Clostridia.Clostridiales.Dehalobacteriaceae	1.990081264	CON	3.067098792	0.047220904

Bacteria.Firmicutes.Clostridia.Clostridiales._Mogibacteriaceae_	2.502043484	CON	2.900777136	0.013874406
Bacteria.Bacteroidetes._Saprospirae_._Saprospirales_.Chitinophagaceae.Sediminibacterium	3.245478136	CON	2.892212354	0.020921335
Bacteria.Bacteroidetes._Saprospirae_._Saprospirales_.Chitinophagaceae	3.245478136	CON	2.86936345	0.020921335
Bacteria.Actinobacteria.Coriobacteriia.Coriobacteriales.Coriobacteriaceae	3.120515219	CON	2.867569101	0.020921335
Bacteria.Actinobacteria.Coriobacteriia.Coriobacteriales.Coriobacteriaceae.Eggerthella	3.099931149	CON	2.85422327	0.020921335
Bacteria.Bacteroidetes._Saprospirae_	3.245478136	CON	2.84994833	0.020921335
Bacteria.Firmicutes.Erysipelotrichi.Erysipelotrichales.Erysipelotrichaceae._Eubacterium_	2.715469799	CON	2.83516256	0.047220904
Bacteria.Firmicutes.Clostridia.Clostridiales.Lachnospiraceae.Anaerostipes	2.794626863	CON	2.828695689	0.020921335
Bacteria.Actinobacteria.Coriobacteriia.Coriobacteriales	3.120515219	CON	2.828626554	0.020921335
Bacteria.Bacteroidetes._Saprospirae_._Saprospirales_	3.245478136	CON	2.821241292	0.020921335
Bacteria.Actinobacteria.Coriobacteriia	3.120515219	CON	2.796772726	0.020921335
Bacteria.Proteobacteria.Alphaproteobacteria.Rhizobiales.Rhizobiaceae	3.323910859	CON	2.786880322	0.043308143
Bacteria.Proteobacteria.Alphaproteobacteria.Rhizobiales.Rhizobiaceae.Agrobacterium	3.323910859	CON	2.765336744	0.043308143

File S4. Relative abundance of metabolites in feces.

Index	Compounds	Class	Con11-1	Con11-2	Con11-3	TD11-1	TD11-2	TD11-3
MEDN005	L-Threonine	Amino Acid metabolomics	362060	342070	331860	175490	168360	210740
MEDN010	L-Citrulline	Amino Acid metabolomics	1038100	1059400	1003700	412400	435270	446830
MEDN013	L-Isoleucine	Amino Acid metabolomics	95851	86603	102240	34801	37204	34033
MEDN022	(5-L-Glutamyl)-L-Amino Acid	Amino Acid metabolomics	238850	297310	255990	519250	537700	459810
MEDN025	3-Hydroxy-3-Methylpentane-1,5-Dioic Acid	Amino Acid metabolomics	1394800	1299100	1387700	411100	473780	529910
MEDN032	Allantoin	Organic Acid And Its Derivatives	130290	187370	149760	360600	407140	354680
MEDN040	Glycyl-L-Proline	Amino Acid metabolomics	67728	57876	46620	68580	63899	81335
MEDN042	L-Asparagine Anhydrous	Amino Acid metabolomics	213430	181500	191630	54862	60978	45988
MEDN045	L-Cysteine	Amino Acid metabolomics	86103	86359	89667	24559	30077	35900
MEDN053	N-Acetylaspartate	Amino Acid metabolomics	75066	61531	70726	100990	76153	65344
MEDN056	N-Acetyl-L-Leucine	Amino Acid metabolomics	134990	145430	140480	164080	166230	161240
MEDN065	O-Phospho-L-Serine	Amino Acid metabolomics	112210	133520	146970	130860	196960	190840
MEDN066	Phenylacetyl-L-Glutamine	Amino Acid metabolomics	50618	48782	53527	105770	88701	85874
MEDN071	S-Sulfo-L-Cysteine	Amino Acid metabolomics	269140	341230	225070	574400	459330	408740
MEDN082	P-Coumaryl Alcohol	Phenols And Its Derivatives	478300	440700	411030	547430	535940	526830
MEDN086	Terephthalic Acid	Benzene and substituted derivatives	463300	566370	509530	469520	469840	502080
MEDN090	2-Methoxybenzoic Acid	Benzoic Acid And Its Derivatives	30408	30843	32712	64328	59985	55204

MEDN092	3,4-Dihydroxybenzeneacetic Acid	Benzene and substituted derivatives	735420	646670	797580	845050	796770	763730
MEDN093	3-Hydroxyanthranilic Acid	Benzoic Acid And Its Derivatives	96780	92110	81277	238660	277170	256120
MEDN097	P–Hydroxyphenyl Acetic Acid	Benzene and substituted derivatives	1102600	1064500	1080800	2310600	2096900	1968700
MEDN098	2-Picolinic Acid	Pyridine And Pyridine Derivatives	1431000	1572300	1515700	62373	88601	113850
MEDN099	4-Pyridoxic Acid	Pyridine And Pyridine Derivatives	2108300	1737000	2076100	1064700	1228600	1210500
MEDN105	Taurocholic Acid	Bile Acids	1686200	2000000	1831700	1012500	852570	725050
MEDN115	Chenodeoxycholic Acid	Bile Acids	219590	210650	217140	52745	109390	90161
MEDN120	Dulcitol	Carbohydrate metabolomics	1517900	1286500	1409500	4185300	5208600	5455900
MEDN122	Inositol	Carbohydrate metabolomics	572740	545980	589570	795210	783630	773490
MEDN124	Protocatechuic Acid	Phenols And Its Derivatives	7168000	7000800	7270600	1691100	1836700	1932500
MEDN128	Vanillin	Phenols And Its Derivatives	18002	19401	15858	37383	37967	35603
MEDN140	2,6-Dihydroxypurine	Nucleotide metabolomics	10471000	8639900	9515900	96269	246130	279930
MEDN159	Flavin Adenine Dinucleotide	Nucleotide metabolomics	88110	109790	100850	19412	32775	33840
MEDN161	Guanosine 3',5'-Cyclic Monophosphate	Nucleotide metabolomics	9	9	9	215170	208900	203610
MEDN166	Nicotinic Acid Adenine Dinucleotide	Nucleotide metabolomics	7213.9	1661.5	5549.1	18310	13882	30485
MEDN170	Uridine 5’-Diphosphate	Nucleotide metabolomics	12845	17830	10659	7385.5	6357.3	11949
MEDN198	Citric Acid	Amino Acid metabolomics	4793600	4302400	4465400	5726900	5007300	4313900
MEDN200	L-Malic Acid	Amino Acid metabolomics	492520	417000	574110	1089400	1131800	1003300

MEDN203	Cis-Aconitic Acid	Amino Acid metabolomics	4329.8	4848.8	4189	3535.3	2288.2	2928.2
MEDN206	Citramalic Acid	Amino Acid metabolomics	479770	399860	422940	902180	1019900	1023900
MEDN208	Melatonin	Tryptamines And Its Derivatives	6871.6	3039.4	3794.4	6154.3	10324	8934.4
MEDN210	1,5-Anhydro-D-Glucitol	Carbohydrate metabolomics	105510	110180	101380	276870	318240	278070
MEDN211	D-Arabitol	Carbohydrate metabolomics	98107	64372	90240	115230	105460	116110
MEDN213	D-Sorbitol	Carbohydrate metabolomics	880450	733010	861650	2750000	3474900	3521600
MEDN214	L-Arabitol	Carbohydrate metabolomics	100990	76036	82429	95568	88981	91392
MEDN220	D-Glucose	Carbohydrate metabolomics	797580	722490	729850	748660	669390	666860
MEDN221	D-Mannose	Carbohydrate metabolomics	1150400	1092900	1019800	811490	904970	916210
MEDN224	D-Trehalose	Carbohydrate metabolomics	237310	213600	271180	340190	224410	160690
MEDN226	D-Erythronolactone	Carbohydrate metabolomics	21854	23456	47667	35922	39845	34117
MEDN227	D-Glucose 6-Phosphate	Carbohydrate metabolomics	2014800	1869000	1893200	3421500	2968300	2871400
MEDN228	D-Arabinose	Carbohydrate metabolomics	1129000	916000	924240	1217400	1267700	1232300
MEDN237	D-Glucoronic Acid	Carbohydrate metabolomics	1433000	1444200	1327200	1591100	1638900	1570700
MEDN240	L-Gulonic-Γ-Lactone	Carbohydrate metabolomics	244600	185660	241980	309050	358750	357320
MEDN245	Pantothenate	CoOthersEnzyme Factor & vitamin	3021800	2908700	2928300	1648000	1922400	2129300
MEDN262	5,6-Dihydroxyindole-2-Carboxylic Acid	Indole And Its Derivatives	15468	13122	11582	8433.6	9134	9819.8
MEDN274	1,7-Dimethyluric Acid	Organic Acid And Its Derivatives	1414.7	1790.4	1901.1	3046.9	1869.2	2741.1
MEDN280	2-Aminoethanesulfonic Acid	Organic Acid And Its Derivatives	5532600	5338000	5395800	1077100	1343400	1538100
MEDN283	2-Hydroxybutanoic Acid	Organic Acid And Its Derivatives	737080	641510	702220	522150	571000	696780

MEDN284	2-Hydroxyisocaproic Acid	Organic Acid And Its Derivatives	1062500	1095000	1058300	2818800	2723400	2733600
MEDN285	2-Methylsuccinic Acid	Organic Acid And Its Derivatives	15121000	14643000	15070000	19777000	23651000	26532000
MEDN290	3-Hydroxy-3-Methyl Butyric Acid	Organic Acid And Its Derivatives	395800	365470	388300	479420	506780	430720
MEDN292	3-Hydroxybutyrate	Organic Acid And Its Derivatives	839050	650490	715490	744590	682070	772340
MEDN299	Adipic Acid	Organic Acid And Its Derivatives	435030	424980	416560	741360	657680	586470
MEDN305	Creatine	Organic Acid And Its Derivatives	100200	84622	87760	121840	135280	145900
MEDN314	Glutaric Acid	Organic Acid And Its Derivatives	14252000	14290000	14296000	19191000	23035000	24315000
MEDN316	Guanidinoethyl Sulfonate	Organic Acid And Its Derivatives	324890	378250	254370	439260	502880	526480
MEDN321	Kinic Acid	Organic Acid And Its Derivatives	793460	693400	727490	1176700	1063300	855220
MEDN324	L-3-Phenyllactic Acid	Organic Acid And Its Derivatives	1699800	1873400	1784800	4896200	4560500	4575000
MEDN325	L-Lactic Acid	Organic Acid And Its Derivatives	1762900	1696000	1683900	2655500	2773900	2817300
MEDN326	L-Tartaric Acid	Organic Acid And Its Derivatives	67977	73886	72778	103430	118490	137080
MEDN333	Malonicacid	Organic Acid And Its Derivatives	763270	792890	683980	889130	895910	757100

MEDN334	Mandelic Acid	Organic Acid And Its Derivatives	1398200	1447300	1455600	2956100	2746800	2546200
MEDN335	Methylmalonic Acid	Organic Acid And Its Derivatives	98101000	92014000	99289000	59920000	72450000	79338000
MEDN338	Phenyllactate (Pla)	Organic Acid And Its Derivatives	515980	549140	537960	3479800	3320100	3151100
MEDN339	Phenylpyruvic Acid	Organic Acid And Its Derivatives	612060	643520	624570	160390	190460	256720
MEDN340	Pyrrole-2-Carboxylic Acid	Organic Acid And Its Derivatives	18382	14505	18050	64178	110120	160440
MEDN343	Shikimic Acid	Organic Acid And Its Derivatives	418180	413870	417560	855030	969000	978610
MEDN362	Lysope 18:1	LipidsOthersPhospholipid	178880	187120	179460	292370	236440	266140
MEDN364	Lysope 18:0	LipidsOthersPhospholipid	299190	329080	408820	772280	621060	815500
MEDN366	Lysope 16:0	LipidsOthersPhospholipid	460440	408670	536590	1260900	1144900	1014900
MEDN376	9,10-DiHOME [(±)9,10-dihydroxy-12Z-octadecenoic acid]	Oxidized lipid	5214.6	4547.5	4087.1	2473.5	2140.6	2167
MEDN381	Hexadecanoic Acid (C16:0)	Lipids_Fatty Acids	27395	28126	20115	26792	32478	28502
MEDN383	Linoleic Acid (C18:2N6C)	Lipids_Fatty Acids	22030	18910	21734	15623	20142	13663
MEDN403	Urocanic Acid	Organic Acid And Its Derivatives	908790	737000	808330	9	9	9
MEDN406	3-(3-Hydroxyphenyl)Propionate Acid	Organic Acid And Its Derivatives	773930	855170	794410	82665	88491	103120
MEDN407	3-Aminosalicylic Acid	Organic Acid And Its	193480	171340	208260	55839	74696	92207

		Derivatives						
MEDN413	Ethylmalonate	Organic Acid And Its Derivatives	99363	89866	95468	127540	149690	159450
MEDN416	Ureidoisobutyric Acid	Organic Acid And Its Derivatives	13510	14368	15669	12380	17850	19315
MEDN417	(Rs)-Mevalonic Acid	Organic Acid And Its Derivatives	148420	133040	170720	302970	313840	228460
MEDN432	5-Hydroxyhexanoic Acid	Organic Acid And Its Derivatives	98835	133350	103460	258340	224730	185930
MEDN441	3,4,5-Trimethoxybenzoic Acid	Benzene and substituted derivatives	2020.7	2508.2	2164.3	2323.4	3025.1	2750.6
MEDN455	Guanine	Nucleotide metabolomics	37711	36633	41937	72973	76865	54092
MEDN463	D-Fructose 6-Phosphate-Disodium Salt	Carbohydrate metabolomics	3054800	3255800	2986300	1764400	1747400	1802300
MEDN469	Citraconic Acid	Organic Acid And Its Derivatives	230810	247030	249050	360050	333990	333320
MEDN471	3-Methoxy-4-Hydroxyphenylethyleneglycol Sulfate	Organic Acid And Its Derivatives	518330	503350	557450	1591100	1637800	1473000
MEDN472	1-Methyluric Acid	Organic Acid And Its Derivatives	353810	363900	364240	301380	317420	330300
MEDN478	Aminomalonic Acid	Organic Acid And Its Derivatives	3736300	3506800	3667300	2258400	2687900	2719100
MEDN481	2,4-Dihydroxybenzoic Acid	Benzene and substituted derivatives	925830	862390	879190	448410	428990	404910

MEDN485	D-Fructose-1,6-Biphosphate-Trisodium Salt	Carbohydrate metabolomics	70825	47614	82270	166770	146530	240430
MEDN486	Palmitaldehyde	Lipids_Fatty Acids	16817	17745	13593	10795	6926.6	10670
MEDN487	Allysine	Organic Acid And Its Derivatives	115180	126220	115940	127770	158060	172420
MEDN490	Fumaric Acid	Amino Acid metabolomics	38335	28070	24955	37881	39147	31764
MEDN496	N-Acetylmethionine	Amino Acid metabolomics	30765	33477	31359	17273	24650	25113
MEDN498	Ribulose-5-Phosphate	Carbohydrate metabolomics	133250	140340	109520	183220	155450	217310
MEDN501	Acetyl Tryptophan	Amino Acid metabolomics	34029	32876	35347	24851	23949	25494
MEDN502	2-Deoxyribose 1-Phosphate	Carbohydrate metabolomics	159370	105200	132750	266490	337670	433640
MEDN506	N-Acetylglucosamine 1-Phosphate	Carbohydrate metabolomics	9151100	9272000	8778600	1284000	1737700	1844400
MEDN523	Indolelactic acid	Indole And Its Derivatives	76505	80537	77984	560930	524490	520610
MEDN528	(3-Methoxy-4-hydroxyphenyl)ethylene glycol sulfate	Organic Acid And Its Derivatives	1841200	1792200	1794100	5197100	5506200	4719500
MEDN530	Pimelic acid	Organic Acid And Its Derivatives	637330	651250	630600	1003200	965360	955600
MEDN531	D-Glucarate	Carbohydrate metabolomics	1058500	1011600	1073000	1970800	1881400	1528800
MEDN533	Xanthosine	Nucleotide metabolomics	828360	791910	848630	174240	130200	335780
MEDN539	3'-Sialyllactose	Carbohydrate metabolomics	155300	129350	164140	251390	217160	160770
MEDN541	DL-3,4-Dihydroxyphenyl glycol	Benzene and substituted derivatives	3103.2	2956.2	2639.5	3425	3144.9	2481
MEDN545	6-Ketoprostaglandin E1	Lipids_Fatty Acids	7038.2	5264.5	6326.3	7579.3	7369.1	4501.6
MEDN551	Indoleacrylic acid	Organic Acid And Its	70284	74399	73668	112570	106890	103610

		Derivatives						
MEDN553	2-Methylbenzoic acid	Benzene and substituted derivatives	13144	8038.9	11815	14786	19774	16106
MEDN554	2-(4-Hydroxyphenyl)ethanol	Benzene and substituted derivatives	6460100	6480800	6877100	4282000	4193200	3980600
MEDN555	Hydroxyphenyllactic acid	Carbohydrate metabolomics	1329000	1293900	1269300	26321000	23922000	21190000
MEDN569	DL-o-Tyrosine	Organic Acid And Its Derivatives	1276900	1035300	1145300	605610	617140	399060
MEDN570	L-Erythrulose	Carbohydrate metabolomics	93276	85297	79143	167020	138520	144900
MEDN579	N-lactoyl-phenylalanine	Organic Acid And Its Derivatives	315090	316340	333280	1162000	1056300	941600
MEDN587	N-Acetylvaline	Organic Acid And Its Derivatives	8698.4	10122	17722	19974	26366	28746
MEDN593	D-(+)-Malic acid	Amino Acid metabolomics	3045000	2901400	2841300	757420	885740	790680
MEDN594	m-Coumaric acid	Benzene and substituted derivatives	5997100	6104400	6029100	1864900	2114000	2476400
MEDN615	Carbamoyl phosphate	Organic Acid And Its Derivatives	14696	21701	15207	100170	91819	61982
MEDN616	O-Acetyl-L-serine	Organic Acid And Its Derivatives	12527	8587.7	8383.9	11399	9338.3	8755.8
MEDN621	Indoxylsulfuric acid	Organic Acid And Its Derivatives	153540	143820	137250	516200	555210	602560
MEDN622	Porphobilinogen	Organic Acid And Its Derivatives	29860	29063	23453	33037	25825	30970
MEDN647	Hydroquinone	Phenols And Its Derivatives	381920	402780	370620	9	9	9

MEDN649	Anthranilic acid	Benzene and substituted derivatives	44238	40902	41842	102970	111810	115650
MEDN651	Succinic anhydride	Organic Acid And Its Derivatives	395450	437520	420100	992480	995140	863040
MEDN662	Gamma-Glu-Leu	Organic Acid And Its Derivatives	146780	142800	143800	195650	176380	184100
MEDN679	Maltol	Heterocyclic compound	10900	10752	8125.9	18940	24824	18525
MEDN686	Methyl propyl disulfide	Others	51501	69437	96604	65635	62715	78383
MEDN689	2,5-Furandicarboxylicacid	Organic Acid And Its Derivatives	73345	52270	47884	49825	76024	66831
MEDN694	2-nonanol	Alcohol	3341	3101.7	5520.3	3905	7590.9	8464.4
MEDN704	Oxaloacetic acid	Organic Acid And Its Derivatives	9	9	9	608770	717700	600510
MEDN706	Butyl??Acetate	Fatty acyls	150550	142590	130190	114700	89301	117910
MEDN720	N-(2-Methylbenzoyl)glycine	Amino Acid metabolomics	3707.8	4025.1	4068.2	9414.6	7001.1	7666
MEDN721	2-Methyl-5-nitroimidazole-1-ethanol	Alcohol	28604	26197	24159	49303	47827	47036
MEDN724	Aspirin	Organic Acid And Its Derivatives	206050	190180	202110	118740	189440	201610
MEDN725	Sorbic acid	Organic Acid And Its Derivatives	1701100	1802100	1842400	1271800	1514200	1490300
MEDN726	Decanal	Aldehyde	50897	66574	71135	55887	73072	74528
MEDN730	4-Ethylphenol	Phenols And Its Derivatives	27807	31815	37684	9	33325	63148
MEDN731	4-tert-Octylphenol	Phenols And Its Derivatives	7263.1	4036.9	4718.9	6998.9	6163.3	3986.7
MEDN734	Cis-3-Hexenylacetate	Fatty acyls	2475800	2645800	2419300	3137500	3512300	4040600

MEDN739	4-Hydroxybenzyl alcohol	Benzene and substituted derivatives	19754	20562	21062	27513	30384	40519
MEDN741	Methanesulfonic acid	Others	635650	640100	600370	636660	602890	620010
MEDN743	2-Nonanone	Ketones	2383300	2597400	2402700	3078200	3521600	4113500
MEDN744	delta-Hexalactone	Lactone	21368	9958.2	22097	9641.3	16413	15063
MEDN748	Methylparaben	Benzoic Acid And Its Derivatives	7279.8	7329.5	6190	2050.7	1480.2	2648.1
MEDN808	Myoinositol	Carbohydrate metabolomics	362670	333020	301910	461120	396790	472180
MEDN811	pelargonate (9:0)	Fatty Acyls	382.02	473.66	520.75	734.31	397.36	431.62
MEDN819	3-Hydroxymandelate	Organic Acid And Its Derivatives	34882	30903	35025	36907	46092	44165
MEDP002	D-Homocysteine	Amino Acid metabolomics	2322500	2221800	1961600	1934200	1884100	1712900
MEDP006	Glycine	Amino Acid metabolomics	265670	329870	226960	359450	245310	231150
MEDP007	L-Cystine	Amino Acid metabolomics	1235900	1240600	1153800	1398800	1217500	1008700
MEDP009	L-Tyrosine	Amino Acid metabolomics	178200000	168670000	170970000	159920000	170610000	161830000
MEDP010	L-Arginine	Amino Acid metabolomics	33544000	34865000	37761000	9113200	8416600	7735700
MEDP011	L-Lysine	Amino Acid metabolomics	52216000	50687000	48913000	12150000	15321000	18204000
MEDP012	L-Ornithine	Amino Acid metabolomics	1684400	1716000	1416600	3571600	3783000	4258400
MEDP013	L-Alanine	Amino Acid metabolomics	18940000	20595000	18372000	11627000	10814000	10888000
MEDP014	L-Aspartic Acid	Amino Acid metabolomics	3334100	3619000	3160100	1990700	2227700	2518100
MEDP016	L-Glutamic Acid	Amino Acid metabolomics	104760000	112780000	101380000	67907000	60879000	60255000
MEDP017	L-Histidine	Amino Acid metabolomics	24405000	28554000	25807000	18759000	17610000	17575000
MEDP019	L-Leucine	Amino Acid metabolomics	555100000	535720000	495020000	353850000	373300000	403460000
MEDP020	L-Methionine	Amino Acid metabolomics	19832000	18602000	17104000	4928500	6454100	6570000
MEDP021	L-Phenylalanine	Amino Acid metabolomics	415750000	424020000	427860000	374030000	373730000	370100000
MEDP022	L-Proline	Amino Acid metabolomics	208540000	220760000	200970000	260240000	246700000	251930000

MEDP024	L-Serine	Amino Acid	metabolomics	7668800	8163200	7593900	8763600	8407500	8041400
MEDP025	L-Tryptophan	Amino Acid	metabolomics	188290000	182470000	187370000	181300000	184940000	170800000
MEDP026	L-Valine	Amino Acid	metabolomics	157170000	170480000	158550000	110400000	119140000	122770000
MEDP031	3-Chloro-L-Tyrosine	Amino Acid	metabolomics	165270	186000	121030	288670	143190	215890
MEDP034	3-N-Methyl-L-Histidine	Amino Acid	metabolomics	314810	362000	372640	1185000	1316100	1132000
MEDP035	5-Hydroxy-L-Tryptopha n	Amino Acid	metabolomics	269740	236200	273530	687240	686760	460830
MEDP036	5-Oxoproline	Amino Acid	metabolomics	1862600	2121800	1448100	628280	1361000	2336400
MEDP037	Asp-Phe	Amino Acid	metabolomics	12615000	12549000	13259000	14535000	13871000	11961000
MEDP039	Betaine	Alkaloid		37948000	40310000	39420000	28173000	28676000	31320000
MEDP041	D-Alanyl-D-Alanine	Amino Acid	metabolomics	485940	452110	468280	459680	433510	375930
MEDP046	Hexanoyl Glycine	Amino Acid	metabolomics	14688	13048	13684	54982	46206	42037
MEDP047	Histamine	Polyamine		8380300	7144600	7735900	3605700	4043300	4960100
MEDP050	L-Carnosine	Amino Acid	metabolomics	602690	416400	438590	373110	433160	355410
MEDP051	L-Cystathionine	Amino Acid	metabolomics	166060	162650	183920	344460	282090	205800
MEDP053	L-Dopa	Amino Acid	metabolomics	1055200	1169000	1117700	358950	638750	605980
MEDP054	L-Glutamine	Amino Acid	metabolomics	39834000	39196000	37742000	20698000	16739000	16685000
MEDP055	L-Homocitrulline	Amino Acid	metabolomics	5478000	5130100	5286100	11360000	10132000	9135700
MEDP056	L-Homocystine	Amino Acid	metabolomics	316260	348150	269020	111530	87132	83886
MEDP058	L-Saccharopine	Amino Acid	metabolomics	7076500	6778000	6794800	4897900	5240200	5404800
MEDP059	L-Theanine	Amino Acid	metabolomics	515300	533240	496980	1259300	1208800	946180
MEDP060	Methionine Sulfoxide	Amino Acid	metabolomics	247510	333530	279370	331470	248920	173030
MEDP061	N,N-Dimethylglycine	Amino Acid	metabolomics	8494400	8547300	8098500	5554700	4932000	4828100
MEDP062	N6-Acetyl-L-Lysine	Amino Acid	metabolomics	38711000	39975000	39122000	100730000	90772000	80728000
MEDP065	N-Acetyl-L-Glutamic Acid	Amino Acid	metabolomics	2802600	2683000	2744500	3648900	3429800	3292200

MEDP066	N-Acetyl-L-Tyrosine	Amino Acid	metabolomics	4861600	4688400	4932600	16846000	15333000	13675000
MEDP067	N-Acetylmannosamine	Amino Acid	metabolomics	24568000	23285000	22772000	1341800	1969200	1712000
MEDP068	N-Acetylneuraminic Acid	Amino Acid	metabolomics	322820	388480	234110	352680	259930	214150
MEDP069	N-Acetylputrescine	Organic Acid And Its Derivatives		2200700	1984500	2072500	4986500	5436600	6015300
MEDP071	N-Glycyl-L-Leucine	Amino Acid	metabolomics	29230000	26691000	28100000	5745300	7717300	8244100
MEDP072	N-Isovaleroylglycine	Amino Acid	metabolomics	9	9	9	21927	28885	12626
MEDP073	N-Phenylacetylglycine	Amino Acid	metabolomics	140030	155730	134070	402680	514380	483970
MEDP075	Nα-Acetyl-L-Arginine	Amino Acid	metabolomics	8201700	8629300	8412200	7552800	7803200	6250400
MEDP078	Phe-Phe	Amino Acid	metabolomics	901050	795850	924100	791990	811320	684650
MEDP079	S-(5-Adenosy)-L-Homocysteine	Amino Acid	metabolomics	9	9	24805	57571	68248	9
MEDP080	S-Adenosyl-L-Methionine	Amino Acid	metabolomics	96220	75314	84996	87136	122180	67967
MEDP081	Serotonin	Indole And Its Derivatives		712520	593460	715140	853770	824180	749770
MEDP083	Trans-4-Hydroxy-L-Proline	Amino Acid	metabolomics	489430000	500460000	480940000	346840000	372510000	402070000
MEDP084	Trimethylamine N-Oxide	Amino Acid	metabolomics	2259300	2272700	2249500	11214000	8985600	6821700
MEDP085	Tyramine	Benzene and substituted derivatives		6140900	5992700	5881500	5156100	6661300	7756000
MEDP086	Urea	Amino Acid	metabolomics	68386	73664	79842	16702000	14313000	13246000
MEDP087	L-Alanyl-L-Lysine	Amino Acid	metabolomics	2006400	2185800	2150200	1766600	1729700	1701200
MEDP089	N-Acetylhistamine	Organic Acid And Its Derivatives		3960700	3441200	3391800	765790	774470	787330

MEDP101	P-Coumaric Acid	Benzene and substituted derivatives	13047000	10805000	11204000	9962600	10321000	8906000
MEDP102	Syringic Acid	Benzene and substituted derivatives	447110	448220	385230	510590	574880	427040
MEDP106	Benzoic Acid	Benzene and substituted derivatives	2452300	2173100	2161500	406480	620620	702690
MEDP107	Methyl Benzoate	Benzene and substituted derivatives	1848500	1398900	1552000	2370000	2156200	1935300
MEDP111	3-(4-Hydroxyphenyl)-Propionic Acid	Benzene and substituted derivatives	3662100	3430800	3663200	9229000	9096200	8379800
MEDP112	4-Ethylbenzoic Acid	Benzene and substituted derivatives	80801	65050	87168	83883	83844	91534
MEDP113	4-Hydroxybenzoic Acid	Benzene and substituted derivatives	11925000	11656000	11682000	8620700	8570300	7692300
MEDP114	Phthalic Acid	Benzene and substituted derivatives	104820	107860	111800	199510	213820	206780
MEDP115	1,4-Dihydro-1-Methyl-4-Oxo-3-Pyridinecarboxamide	Pyridine And Pyridine Derivatives	21729000	22484000	22566000	577520	833790	1302000
MEDP119	6-Hydroxynicotinic Acid	Pyridine And Pyridine Derivatives	1108000	1059900	1084600	913620	1299200	1614000
MEDP120	Theobromine	Nucleotide metabolomics	9	33418	9	322420	338560	266370
MEDP123	Acetylcholine Chloride	Cholines	870640	877710	831320	1434600	1339600	1252100
MEDP125	Choline	Cholines	1016300	978990	906580	592410	544570	521610
MEDP126	1,5-Diaminopentane	Polyamine	365480	445170	463140	330280	352590	453840
MEDP127	Putrescine	Polyamine	35388	53277	63564	59074	41464	37629

MEDP128	Diethanolamine	Polyamine	166360	195310	165400	205630	139930	146000
MEDP129	Phenethylamine	Polyamine	704700	780850	721690	687130	794880	748600
MEDP130	4-Nitrophenol	Phenols And Its Derivatives	781830	822520	668680	742160	564460	785290
MEDP143	Biotin	CoOthersEnzyme Factor & vitamin	831810	916270	900060	298860	337260	318800
MEDP145	1,7-Dimethylxanthine	Nucleotide metabolomics	22504	24035	23327	51719	61013	41389
MEDP146	1-Methyladenine	Nucleotide metabolomics	180940	9	122440	266820	321080	315840
MEDP147	1-Methylhistidine	Amino Acid metabolomics	6474000	6395200	6098500	13823000	14119000	14686000
MEDP148	1-Methylxanthine	Nucleotide metabolomics	4371800	4362900	4660900	867630	1044200	1201200
MEDP149	2'-Deoxyadenosine-5'-Monophosphate	Nucleotide metabolomics	218670	168240	182280	614850	585250	565490
MEDP150	2'-Deoxyinosine	Nucleotide metabolomics	676470	677900	648160	135890	218610	368250
MEDP151	2-Hydroxy-6-Aminopuri ne	Nucleotide metabolomics	1670300	1757200	1449600	611420	807700	1560800
MEDP153	3-Methylxanthine	Nucleotide metabolomics	760150	776680	837640	366090	601050	751120
MEDP154	5,6-Dihydro-5-Methylur acil	Nucleotide metabolomics	375580	340370	369110	391760	490990	510960
MEDP155	5-Methylcytosine	Nucleotide metabolomics	560680	456660	559310	969590	923140	1096100
MEDP156	5-Methyluridine	Nucleotide metabolomics	9	9	9	135740	139930	119230
MEDP158	8-Hydroxyguanosine	Nucleotide metabolomics	70601	207740	88890	112670	122400	83616
MEDP159	Adenine	Nucleotide metabolomics	7437400	6528500	6317900	6501600	7782100	7826200
MEDP160	Adenosine	Nucleotide metabolomics	23219000	21937000	22766000	10545000	9422400	6904600
MEDP161	Adenosine 5'-Diphosphate	Nucleotide metabolomics	89136	84772	79434	96416	102430	102720
MEDP163	Cytidine	Nucleotide metabolomics	1460700	1341800	1257800	424930	398710	233310
MEDP164	Cytidine-5-Monophosph	Nucleotide metabolomics	102730	147090	83092	264380	157490	248050

	ate							
MEDP165	Cytosine	Nucleotide metabolomics	2077100	1986500	1819600	2775800	2276500	2216900
MEDP166	Deoxyguanosine	Nucleotide metabolomics	6133700	5864700	4765200	272560	539060	715410
MEDP167	Guanosine	Nucleotide metabolomics	1389900	1371900	1287900	550560	478080	449240
MEDP169	Guanosine Monophosphate	Nucleotide metabolomics	12367	13373	19399	753050	400170	302700
MEDP170	Hypoxanthine	Nucleotide metabolomics	1193000	582850	640260	1786700	3448300	3249900
MEDP171	Inosine	Nucleotide metabolomics	918870	820180	860560	384500	472720	375510
MEDP174	Purine	Nucleotide metabolomics	917110	855220	866120	756530	1095000	1247800
MEDP176	Thymidine	Nucleotide metabolomics	1601200	1458900	1468500	868760	891700	814400
MEDP177	Thymine	Nucleotide metabolomics	7100600	6671500	6996200	4971100	6026000	6515000
MEDP178	Uracil	Nucleotide metabolomics	1246800	1160000	1192900	188540	339350	403220
MEDP179	Uridine	Nucleotide metabolomics	349290	381960	303740	248390	232970	172680
MEDP180	B-Nicotinamide Mononucleotide	Nucleotide metabolomics	171250	139960	139100	1793800	1172200	644190
MEDP184	3,3',5-Triiodo-L-Thyronine	Hormones	36097	37181	38293	44649	42511	47638
MEDP185	Epinephrine	Hormones	11509000	10639000	11231000	8641400	8780000	8702300
MEDP186	L-Thyroxine	Hormones	24117	22334	25018	16255	20162	24887
MEDP188	Norepinephrine	Hormones	2355600	3830700	3265700	3709500	2752100	2961800
MEDP189	Progesterone	Hormones	24426	23307	22107	31158	24644	33733
MEDP205	L-Carnitine	Camitine	953720	1044400	1037300	949820	940970	912410
MEDP208	Succinic Acid	Amino Acid metabolomics	3933600	4003900	4213100	3347200	4050200	4088100
MEDP212	N-Acetyl-5-Hydroxytryptamine	Tryptamines And Its Derivatives	357940	358560	363810	81909	130200	191190
MEDP214	Tryptamine	Tryptamines And Its	227150	234070	280250	55177	53028	67209

		Derivatives						
MEDP217	Dl-Threitol	Carbohydrate metabolomics	426040	458170	369160	482570	490360	575380
MEDP218	D-Mannitol	Carbohydrate metabolomics	1782500	1568000	1836600	10882000	11057000	10318000
MEDP221	Ribitol	Carbohydrate metabolomics	40588000	9	42475000	9	9	40823000
MEDP224	D-Fructose	Carbohydrate metabolomics	55813000	55577000	53988000	44987000	39800000	37501000
MEDP228	D-Melezitose	Carbohydrate metabolomics	133280	121050	143910	176020	84220	89783
MEDP229	Lactose	Carbohydrate metabolomics	186410	187680	194740	105300	100250	112860
MEDP230	Maltose	Carbohydrate metabolomics	18088	9	37309	394950	247010	116180
MEDP232	N-Acetyl-D-Glucosamin e	Carbohydrate metabolomics	7224700	7196400	7237300	995970	1195500	932900
MEDP233	Raffinose	Carbohydrate metabolomics	146940	122010	166180	130070	97168	86872
MEDP234	D-Glucono-1,5-Lactone	Carbohydrate metabolomics	313950	268640	260700	319480	293500	221450
MEDP236	Gluconic Acid	Carbohydrate metabolomics	702650	601480	786520	587070	429910	735350
MEDP238	Vitamin D3	CoOthersEnzyme Factor & vitamin	19963	13270	14975	40418	36801	48202
MEDP239	L-Ascorbate	CoOthersEnzyme Factor & vitamin	1093100	1500400	1864200	9	1121500	1031300
MEDP240	Pyridoxine	CoOthersEnzyme Factor & vitamin	5277500	4672600	4915800	4254600	4164000	4225100
MEDP241	Orotic Acid	CoOthersEnzyme Factor & vitamin	3954900	3688400	3641900	1412400	1734400	1963900
MEDP242	Nicotinamide	CoOthersEnzyme Factor & vitamin	235760	202540	211790	284950	408440	410470
MEDP244	All-Trans-13,14-Dihydro retinol	CoOthersEnzyme Factor & vitamin	9	9	9	30513	47774	55278
MEDP246	Nicotinic Acid	CoOthersEnzyme Factor &	39728000	39756000	40602000	4130200	5657100	6727200

		vitamin						
MEDP247	Nicotinuric Acid	CoOthersEnzyme Factor & vitamin	209160	338170	260830	496040	624280	572440
MEDP248	N-Methylnicotinamine	CoOthersEnzyme Factor & vitamin	67553	74340	86690	129480	146440	128650
MEDP249	Pantothenol	CoOthersEnzyme Factor & vitamin	11658	7914.7	12693	37017	6805.7	59518
MEDP250	Riboflavin	CoOthersEnzyme Factor & vitamin	19563000	19827000	20139000	6694100	7278400	7247200
MEDP251	Trigonelline	CoOthersEnzyme Factor & vitamin	62999000	63988000	65584000	151750000	145780000	125970000
MEDP271	3-Indolepropionic Acid	Indole And Its Derivatives	8701700	8450500	8758600	396370	614310	839910
MEDP272	5-Hydroxyindole-3-Acetic Acid	Indole And Its Derivatives	247720000	243300000	241630000	103720000	127880000	139820000
MEDP273	5-Hydroxytryptophol	Indole And Its Derivatives	148160	124070	168300	326080	293330	260330
MEDP274	Indole-2-Carboxylic Acid	Indole And Its Derivatives	27382	9	9	793150	674020	425920
MEDP275	Indole-3-Acetic Acid	Indole And Its Derivatives	9	9	9	384590	356410	352710
MEDP276	Indole-3-Carboxaldehyde	Indole And Its Derivatives	1107700	1031700	1079900	1673300	1804200	1817300
MEDP277	Methyl Indole-3-Acetate	Indole And Its Derivatives	2869100	2626000	2701500	3548500	3478700	3270900
MEDP282	1,3-Dimethyluric Acid	Organic Acid And Its Derivatives	206240	181840	202020	37193	40423	64914
MEDP284	1-Naphthylacetic Acid	Organic Acid And Its Derivatives	15379	10590	21952	14292	19581	28604
MEDP285	2,6-Diaminooimelic Acid	Organic Acid And Its	3162500	3391300	2916100	7574700	5924000	5315200

		Derivatives						
MEDP288	2–Furanoic Acid	Organic Acid And Its Derivatives	87687	110860	90478	106440	183570	172520
MEDP289	3,4,5-Trimethoxycinnamic Acid	Organic Acid And Its Derivatives	141550	99519	99123	35727	76931	53180
MEDP290	3,5-Dimethoxy-4-Hydroxycinnamic Acid	Organic Acid And Its Derivatives	482070	445000	468430	436820	439590	457210
MEDP291	3,7-Dimethyluric Acid	Organic Acid And Its Derivatives	139420	134240	158840	37168	65004	76596
MEDP294	3-Methylcrotonyl Glycine	Organic Acid And Its Derivatives	32382	28400	42342	41793	54611	51671
MEDP295	4-Acetamidobutyric Acid	Organic Acid And Its Derivatives	5112200	5046600	5059200	11236000	11275000	10232000
MEDP296	4-Guanidinobutyric Acid	Organic Acid And Its Derivatives	3107400	2420100	2647700	4069700	3811000	3615700
MEDP297	5-Aminovaleric Acid	Organic Acid And Its Derivatives	64912000	66460000	62791000	16455000	17981000	19020000
MEDP298	6-Aminocaproic Acid	Organic Acid And Its Derivatives	94437000	90651000	92763000	45536000	48938000	52739000
MEDP299	7-Methyluric Acid	Organic Acid And Its Derivatives	1115900	983550	926300	1130300	1138000	1168800
MEDP301	Azelaic Acid	Organic Acid And Its Derivatives	901480	896250	873910	1294600	1237700	1141500
MEDP302	Caffeic Acid	Organic Acid And Its Derivatives	1233600	1247300	1183200	177100	216160	301260
MEDP303	Chlorogenic Acid	Organic Acid And Its	285010	270570	301480	180450	303110	446230

		Derivatives						
MEDP305	Creatinine	Organic Acid And Its Derivatives	2642200	2453200	2694600	3782600	3778400	3782400
MEDP307	Dl-2-Aminooctanoic Acid	Organic Acid And Its Derivatives	106060	107700	92790	141090	186550	207500
MEDP308	Dodecanedioic Aicd	Organic Acid And Its Derivatives	5833200	5390800	5631200	143130	195110	216400
MEDP310	D-Pipecolinic Acid	Organic Acid And Its Derivatives	1447800	1336300	1530800	1420300	1572100	1993800
MEDP313	Guanidineacetic Acid	Organic Acid And Its Derivatives	72661000	69126000	68798000	17204000	18578000	20273000
MEDP315	Hippuric Acid	Organic Acid And Its Derivatives	1349900	1309900	1350500	2460200	2355000	2154300
MEDP316	Homovanillic Acid	Organic Acid And Its Derivatives	159460	162300	202180	821700	817910	784910
MEDP317	Hydrocinnamic Acid	Organic Acid And Its Derivatives	9	9	9	189110	188730	155860
MEDP318	Kinurenine	Organic Acid And Its Derivatives	240250	212120	228970	173500	230710	228070
MEDP319	Kynurenic Acid	Organic Acid And Its Derivatives	4962400	4765600	5030800	15236000	24811000	32872000
MEDP321	L-Dihydroorotic Acid	Organic Acid And Its Derivatives	19689000	16193000	16793000	13416000	15029000	18499000
MEDP322	L-Homoserine	Organic Acid And Its Derivatives	9929200	10585000	10287000	8318900	8026100	7085700
MEDP325	Maleic Acid	Organic Acid And Its	14084000	14690000	12317000	19596000	19163000	18166000

		Derivatives						
MEDP326	N'-Formylkynurenine	Organic Acid And Its Derivatives	231030	270590	292170	9	33164	36214
MEDP327	N-Γ-Acetyl-N-2-Formyl-5-Methoxykynurenamine	Organic Acid And Its Derivatives	114260	112170	114190	171250	171200	169620
MEDP328	P-Aminohippuric Acid	Organic Acid And Its Derivatives	9	11089	19831	25781	10508	6092.9
MEDP331	Subericacid	Organic Acid And Its Derivatives	1378200	1389300	1413600	2186600	2080900	2021900
MEDP332	Cinnamic Acid	Organic Acid And Its Derivatives	12187000	11712000	12344000	6333600	6702100	6489100
MEDP333	Uric Acid	Organic Acid And Its Derivatives	121500000	111940000	108090000	151910000	154300000	166520000
MEDP334	Vanillic Acid	Organic Acid And Its Derivatives	6362400	6455200	6399600	2358100	2541300	2724700
MEDP336	Lysopc 14:0	LipidsOthersPhospholipid	1888100	1901900	2028400	3921700	4251700	4533300
MEDP338	Lysopc 16:0	LipidsOthersPhospholipid	102740000	100140000	120610000	226030000	196530000	179910000
MEDP340	Lysopc 16:1	LipidsOthersPhospholipid	4529000	4207000	4664400	7223500	7783000	7250400
MEDP342	Lysopc 18:0	LipidsOthersPhospholipid	111360000	113790000	131170000	274780000	259100000	288210000
MEDP344	Lysopc 18:1	LipidsOthersPhospholipid	83078000	77735000	94751000	208110000	173410000	152340000
MEDP346	Lysopc 18:2	LipidsOthersPhospholipid	26084000	21210000	31974000	85629000	67266000	41226000
MEDP350	Lysopc 20:1	LipidsOthersPhospholipid	3354200	3438400	3949600	14595000	13128000	14149000
MEDP352	Lysopc 20:2	LipidsOthersPhospholipid	2369700	2277600	2338700	6496800	6251200	6953300
MEDP356	Methyl Nicotinate	CoOthersEnzyme Factor & vitamin	899900	821580	862830	169410	239840	253190
MEDP361	4-Hydroxybenzaldehyde	Benzene and substituted	2534100	2349200	2509200	94045000	75607000	54720000

		derivatives						
MEDP362	Neopterin	Pteridines and derivatives	27433	39884	46292	27644	44145	25714
MEDP363	Biopterin	Pteridines and derivatives	431410	333020	281040	788360	864130	1036200
MEDP367	γ-Aminobutyric Acid	Organic Acid And Its Derivatives	408590	407590	406400	506030	697920	862480
MEDP368	Taurocholic Acid Sodium Salt Hydrate	Organic Acid And Its Derivatives	1590900	2223400	1534200	2370800	2275200	1605700
MEDP369	4-(Aminomethyl)-5-(Hydroxymethyl)-2-Methylpyridin-3-Ol	Pyridine And Pyridine Derivatives	1533500	1694200	1771800	551850	667220	742810
MEDP370	2-(Formylamino)Benzoic Acid	Benzene and substituted derivatives	516960	476990	475360	889980	834710	672410
MEDP371	Acetaminophen Glucuronide	Carbohydrate metabolomics	9	9	9	89644	91946	66423
MEDP372	Uridine 5-Monophosphate	Nucleotide metabolomics	75536	107960	62511	192660	132630	84669
MEDP373	N-Acetylglycine	Amino Acid metabolomics	3933900	3331000	3962100	4829200	5318600	5865800
MEDP374	Cyclic Amp	Nucleotide metabolomics	78680	69969	68484	5722400	4967400	4257400
MEDP375	Deoxyadenosine	Nucleotide metabolomics	10259000	9433900	8736100	2534000	4379000	5553400
MEDP378	N6-Succinyl Adenosine	Nucleotide metabolomics	2545200	2487900	2626900	17159000	13908000	7370700
MEDP379	3-Hydroxyhippuric Acid	Organic Acid And Its Derivatives	34760	25158	24423	88501	86617	105230
MEDP380	2-(Dimethylamino)Guanosine	Nucleotide metabolomics	945700	913630	894760	2750500	2452400	6946000
MEDP381	7-Methylguanine	Nucleotide metabolomics	233400000	229770000	233970000	150820000	155760000	153750000
MEDP382	8-Hydroxy-2-Deoxyguan	Nucleotide metabolomics	425710	435230	354380	139770	215320	225230

	osine							
MEDP383	B-Pseudouridine	Nucleotide metabolomics	962980	782800	778760	650310	566320	680260
MEDP384	Isoxanthopterin	Pteridines and derivatives	272230	272380	241700	661860	593750	828320
MEDP385	L-Sepiapterin	Pteridines and derivatives	9	9	9	22790	10727	13971
MEDP386	N-Acetylthreonine	Amino Acid metabolomics	1562000	1654500	1630400	4722100	4600600	4670600
MEDP387	H-Homoarg-Oh	Amino Acid metabolomics	1639700	1783600	1892800	2020600	2374100	1611100
MEDP389	Pantetheine	CoOthersEnzyme Factor & vitamin	40403000	35574000	34531000	39414000	40311000	40440000
MEDP390	Hypoxanthine-9-β-D-Arabinofuranoside	Nucleotide metabolomics	834710	701980	714830	9	498480	443190
MEDP391	Menaquinone	CoOthersEnzyme Factor & vitamin	1413100	857530	748470	1305900	696030	784560
MEDP393	Pyridoxine 5'-Phosphate	CoOthersEnzyme Factor & vitamin	79403	102560	135500	377860	322020	238950
MEDP395	L-Pipecolic Acid	Amino Acid metabolomics	777150	654120	606020	872450	894200	702910
MEDP396	2'-Deoxycytidine-5'-Monophosphate	Nucleotide metabolomics	9	9	9	32959	46875	53906
MEDP401	5'-Deoxy-5'-(Methylthio) Adenosine	Nucleotide metabolomics	373600	384950	402450	844990	700930	804290
MEDP403	Deoxycytidine	Nucleotide metabolomics	2876000	3027500	2448100	409910	398590	446360
MEDP405	3-Aminoisobutanoic Acid	Amino Acid metabolomics	513910	487650	477930	370770	544940	635510
MEDP407	Vitamin A	CoOthersEnzyme Factor & vitamin	116420	134120	149680	44116	72413	59994
MEDP408	11-Cis-Retinol	CoOthersEnzyme Factor & vitamin	117040	122340	119990	60884	88652	115580

MEDP410	Trans-Citridic Acid	Organic Acid And Its Derivatives	220130	190540	240470	224030	163890	246470
MEDP416	6-Phosphogluconic Acid Trisodium Salt	Carbohydrate metabolomics	425030	382720	314700	240060	238890	289050
MEDP423	Phosphoric Acid	Organic Acid And Its Derivatives	6560900	9	6521700	9	21624000	9073000
MEDP425	Imidazoleacetic acid	Indole And Its Derivatives	1483400	1442200	1426600	663110	657820	629480
MEDP426	Xanthurenic Acid	Organic Acid And Its Derivatives	26015	24925	27346	26954	26422	18039
MEDP428	Cholesterol	Lipids	782770	416910	620280	740120	642810	612460
MEDP429	Punicic Acid	Lipids_Fatty Acids	9889500	9554400	9198000	8026700	10459000	5860100
MEDP430	2-Aminoadipic Acid	Organic Acid And Its Derivatives	3208200	3246900	3005300	2857000	2674900	2267400
MEDP431	Hordenine	Benzene and substituted derivatives	845710	823490	846520	447530	450640	452090
MEDP434	Lysopc 17:0	LipidsOthersPhospholipid	2568000	2528600	2536400	6281900	6675600	8017200
MEDP435	6-Methylmercaptopurine	Nucleotide metabolomics	82265000	80780000	81286000	44651000	46790000	45351000
MEDP436	N-Feruloyl Putrescine	Phenolamides	19416000	19415000	23239000	30549000	31778000	28936000
MEDP437	Ergothioneine	Organic Acid And Its Derivatives	377600	391810	446880	942510	963260	1025100
MEDP440	Methoxyindoleacetic Acid	Indole And Its Derivatives	6108000	5894000	6031300	5924800	5829600	5285900
MEDP441	N-Caffeoyl Putrescine	Phenolamides	359730	376090	351770	51341	64694	64027
MEDP442	Sn-Glycero-3-Phosphocholine	Cholines	68916	63546	54800	584380	428230	341910

MEDP443	10-Formyl-Thf	Pteridines and derivatives	10795000	10831000	11023000	13590000	13694000	13281000
MEDP446	3-Hydroxykynurenine	Amino Acid metabolomics	435830	474070	489490	604810	473620	475220
MEDP450	P-Aminobenzoate	Benzene and substituted derivatives	861950	700350	694740	1403500	1891700	2058000
MEDP453	Indole	Indole And Its Derivatives	6444800	6277000	6474000	6793900	6575300	6056200
MEDP454	Isoquinoline	Benzene and substituted derivatives	7395400	7154100	7472400	3019200	3537600	3851800
MEDP455	Dihydrojasmone	Organic Acid And Its Derivatives	286300	242730	287860	682760	650910	618570
MEDP456	Piperidine	Benzene and substituted derivatives	16343000	15440000	15011000	7676900	8611200	9135000
MEDP457	5-Aminolevulinate	Organic Acid And Its Derivatives	1339000	1179100	1047200	539610	712620	703560
MEDP461	2-Methylguanosine	Nucleotide metabolomics	3229900	3497700	2927900	11216000	8935400	24491000
MEDP494	Lysopc 15:0	LipidsOthersPhospholipid	3480500	3219500	3520300	5947900	6650800	7311500
MEDP495	Lysope 18:2 (2N Isomer)	Lipids_Fatty Acids	5705.5	7370.3	8830.1	16754	15572	11395
MEDP498	Lysopc 18:3	LipidsOthersPhospholipid	3488900	3380100	4421700	8674400	8103000	6450200
MEDP504	Glucosamine	Carbohydrate metabolomics	2765200	2961900	2710000	3508400	4257800	3896500
MEDP507	2-Hydroxycinnamic acid	Benzene and substituted derivatives	75112000	65776000	65220000	64731000	66650000	57714000
MEDP509	trans-3-Indoleacrylic acid	Organic Acid And Its Derivatives	53297000	53173000	54859000	51052000	52193000	46777000
MEDP510	Acetyl-L-carnitine	Camitine	2237200	2383100	2520700	4380900	3440400	3547400
MEDP511	Jasmonic acid	Lipids_Fatty Acids	9	231510	9	380880	359360	350650
MEDP513	Hexadecanamide	Lipids_Fatty Acids	13511000	8873800	10770000	16700000	7135600	6865400
MEDP514	Thiamine	CoOthersEnzyme Factor &	9	9	9	3598400	3737300	2421200

MEDP517	Palmitoylcarnitine	vitamin Camitine	9	9	9	30241	32143	36751
MEDP518	DL-Stachydrine	Organic Acid And Its Derivatives	55685000	50767000	59502000	50795000	49072000	44831000
MEDP519	L-Norleucine	Amino Acid metabolomics	9461300	9644700	10863000	10388000	9886800	8778100
MEDP523	DL-Carnitine	Camitine	2853200	2881400	2865000	2011200	1972500	2286000
MEDP525	N-Acetyl-L-alanine	Organic Acid And Its Derivatives	27197000	27385000	27506000	41567000	42213000	40890000
MEDP529	Indole-3-acetamide	Indole And Its Derivatives	916320	817660	797390	1870400	1925900	1894600
MEDP531	(-)-trans-Caryophyllene	Lipids_Fatty Acids	1211900	1112800	1187900	1142100	1133500	1006800
MEDP535	Triethyl phosphate	Organic Acid And Its Derivatives	360510	357600	364270	359990	388780	415820
MEDP537	PAF C-16	Lipids	114110000	121840000	136100000	273180000	274120000	303480000
MEDP539	Ubiquinone-1	Lipids_Fatty Acids	13802000	13825000	13618000	6431400	6388800	6294100
MEDP540	7,8-dihydro-L-Biopterin	Pteridines and derivatives	726550	666740	601560	2774500	2164400	3342200
MEDP541	δ-Valerolactam	Pyridine And Pyridine Derivatives	6325100	6294400	6689600	10101000	10464000	10678000
MEDP545	2-Pyrrolidinone	Benzene and substituted derivatives	14017000	13314000	12376000	8145900	9584600	10196000
MEDP546	Oxindole	Indole And Its Derivatives	9	9	9	349520	377380	350970
MEDP550	18-Hydroxycorticosterone	Lipids	206640	215220	202550	495240	611950	822210
MEDP551	N-METHYL (-)EPHEDRINE	Benzene and substituted derivatives	496630	520060	479590	180190	237390	226170
MEDP556	Herniarin	Benzene and substituted derivatives	838900	1012400	727820	1548500	1675200	1779100

MEDP561	Farnesene	Lipids_Fatty Acids	223010	222960	227010	230930	232150	259800
MEDP574	Oleamide	Lipids_Fatty Acids	33858000	22623000	26750000	40345000	14229000	11277000
MEDP575	5-amino-1-[3,4-dihydroxy-5-(hydroxymethyl)oxolan-2-yl]imidazole-4-carboxamide	Nucleotide metabolomics	9	9	9	9	9	47076
MEDP577	Isobutyryl carnitine	Camitine	1385200	1442600	1612500	2503800	2351000	1920700
MEDP578	alpha-Cadinene	Lipids_Fatty Acids	146790	152330	154600	164420	161850	149090
MEDP579	gamma-Muurolene	Lipids_Fatty Acids	1160200	1152600	1179500	1124200	1138400	999640
MEDP583	Asp-Phe methyl ester	Organic Acid And Its Derivatives	15684000	14489000	16435000	18241000	16745000	14361000
MEDP584	Pterine	Pteridines and derivatives	569990	555620	520100	1220700	1341300	1156200
MEDP585	Stearidonic Acid	Lipids_Fatty Acids	1128100	1085500	1008800	444130	870690	415480
MEDP586	N-Acetylphenylalanine	Organic Acid And Its Derivatives	12758000	12294000	12789000	25295000	22766000	18959000
MEDP587	N-Alpha-acetyllysine	Organic Acid And Its Derivatives	39377000	39857000	38983000	85393000	83217000	79224000
MEDP588	Ritalinic acid	Organic Acid And Its Derivatives	374240	172330	187800	575800	976580	1031600
MEDP590	p-Mentha-1,3,8-triene	Lipids_Fatty Acids	2516000	2575000	1224200	520030	596880	628790
MEDP593	Thioguanine	Nucleotide metabolomics	115660	136400	128900	82554	124480	111560
MEDP594	Dihydroactinidiolide	Benzene and substituted derivatives	823230	901110	794460	351360	386360	417330
MEDP598	2'-Hydroxy-5'-methylacetophenone	Benzene and substituted derivatives	207440	146720	177710	87186	91077	134980
MEDP602	1-(4-Methoxyphenyl)-2-	Benzene and substituted	547190	603570	558170	7876600	6929500	6308900

	propanone	derivatives						
MEDP603	Cuminaldehyde	Lipids_Fatty Acids	13068000	12435000	13052000	6756500	6994300	6873900
MEDP606	2-(3,4-dimethoxyphenyl)ethanamine	Benzene and substituted derivatives	69735000	63502000	60135000	59986000	60707000	52631000
MEDP611	N-Acetyl-L-Histidine	Organic Acid And Its Derivatives	6041700	6080100	6192700	6480100	6311300	6241300
MEDP612	(S)-2,3-epoxysqualene	Terpenoid	413530	374900	422330	328620	365990	386040
MEDP617	2-Furoylglycine	Amino Acid metabolomics	533170	668320	562540	1424000	1177700	906430
MEDP618	2-Methylbutyroylcarnitine	Lipids_Fatty Acids	383950	521220	377430	1109100	1064800	821630
MEDP627	Isonicotinic acid	Organic Acid And Its Derivatives	8975900	9192900	9116200	9	9	1359400
MEDP631	Dimethyl fumarate	Lipids_Fatty Acids	58811000	54082000	59066000	50563000	48418000	44527000
MEDP632	beta-Cubebene	Lipids_Fatty Acids	177630	277230	250860	201200	334570	216680
MEDP636	Furfural	Organic Acid And Its Derivatives	481320	525880	469180	284910	342380	307520
MEDP637	L-phenylalanyl-L-proline	Organic Acid And Its Derivatives	18229000	18524000	18811000	2959800	3982600	4040100
MEDP638	LysoPE(16:1(9Z)/0:0)	Lipids_Fatty Acids	22256	25142	19548	33711	59941	79001
MEDP642	3-Hydroxy-DL-kynurenine	Amino Acid metabolomics	305200	327560	364910	869740	884110	749110
MEDP652	Methyl cinnamate	Benzene and substituted derivatives	111300	105840	94130	91468	91436	95236
MEDP653	Benzylcinnamate	Benzene and substituted derivatives	96584	59680	91130	91236	110100	99943
MEDP654	2-Phenylacetamide	Benzene and substituted	176100	183540	182590	274980	289340	280050

		derivatives						
MEDP656	Isoamyl butyrate	Fatty acyls	58009000	61978000	60568000	52186000	60748000	57304000
MEDP658	Ethyl dodecanoate	Fatty acyls	6188700	5368400	5512600	5990300	5573700	6030200
MEDP659	Propylpropionate	Fatty acyls	4568300	5156300	4382300	5960100	3521500	2642500
MEDP664	L- tyrosine methyl ester	Amino Acid metabolomics	680990	645230	698800	521580	614810	644660
MEDP665	Methyl isobutyl ketone	Ketones	3639000	3601700	3650400	513540	606710	529410
MEDP668	m-Cresol	Phenols And Its Derivatives	185290	181520	166060	164520	116660	145550
MEDP676	Butyl 3-methylbutanoate	Fatty acyls	21593000	18485000	20499000	18086000	18589000	17075000
MEDP680	Hexylamine	Amines	345170	336160	298670	54279	63206	64374
MEDP681	2,2,2-Trichloroethanol	Alcohol	19443000	20204000	19002000	19947000	20758000	21215000
MEDP685	Methylcysteine	Amino Acid metabolomics	24025000	22631000	22634000	21632000	19496000	17741000
MEDP686	2-Acetylfuran	Heterocyclic compound	16916000	18188000	16913000	17275000	16694000	17294000
MEDP688	Benzophenone	Benzene and substituted derivatives	32425	34919	37776	32106	31476	28359
MEDP689	TranexamicAcid	Organic Acid And Its Derivatives	67915	79215	97718	120280	100080	101460
MEDP692	Triethylamine	Hydrocarbon derivative	2054400	1688700	1908800	1749800	1547400	1590400
MEDP696	Octadecanamide	Fatty acyls	36623	31099	41431	41141	32391	38519
MEDP697	2,6-Di-tert-butyl-4-methylphenol	Benzene and substituted derivatives	369040	589780	563030	536670	519760	1194000
MEDP700	Methyl??beta-D-galactopyranoside	Carbohydrate metabolomics	3308600	3259600	3214800	2240000	2416000	2377700
MEDP701	(E)-2-Octen-1-ol	Alcohol	2446100	1984400	2021300	3981400	3329200	3317400
MEDP703	Atrazine	Heterocyclic compound	28952	17471	18891	33067	27285	31186
MEDP707	2-Pentadecanone	Ketones	1860400	1657300	1812300	1728600	1683100	1809800

MEDP709	Methyl dihydrojasmonate	Organic Acid And Its Derivatives	2640000	2710600	2535000	2384500	2351000	2507700
MEDP716	cis-Citral	Aldehyde	355050	467090	289440	283040	235680	284240
MEDP718	Methylisobutyrate	Organic Acid And Its Derivatives	184910	218030	229170	678680	657790	534960
MEDP719	N-Nitrosodiethylamine	Polyamine	239480	249160	247530	478490	557070	499080
MEDP724	3,5-Dinitrosalicylic acid	Organic Acid And Its Derivatives	1887100	1848100	1888800	2135500	2014000	2083700
MEDP729	Barbituric acid	Heterocyclic compound	1996200	1980400	2013600	3431600	3384000	2840900
MEDP733	o-Xylene	Benzene and substituted derivatives	4544200	4789700	4984000	4641000	4386600	4401700
MEDP734	2-Aminophenol	Phenols And Its Derivatives	73472	88070	89115	82926	78986	86059
MEDP736	1,3-diisopropylbenzene	Benzene and substituted derivatives	152450	184850	224780	105670	170210	109200
MEDP738	carene	Heterocyclic compound	189370	165730	175770	167850	160880	183930
MEDP739	Hexyl??Acetate	Fatty acyls	107580	114090	120570	292820	291530	272400
MEDP742	Terpinolene	Terpenoid	3935.4	2514.9	4503.5	4052.4	2290.7	2853.9
MEDP747	alpha-Terpinene	Terpenoid	6735600	6682300	6758400	6802100	6973300	6781000
MEDP752	DL-Leucine	Amino Acid metabolomics	567740000	561130000	566650000	376330000	391050000	402290000
MEDP765	3-(Methylthio)-1-propanol	Alcohol	284950	402980	296800	506890	601970	755880
MEDP773	Norambreinolide	Lactone	40487	35868	32483	33116	37514	37868
MEDP777	Diethyl malonate	Fatty acyls	87955	89860	79802	88308	93075	89492
MEDP789	2-Pentyl-3-phenyl-2-propenal	Aldehyde	77276	80189	88434	85929	75089	84127
MEDP792	PYRROLIDINE	Heterocyclic compound	2995600	2932600	2893100	1474500	1740100	1783400

MEDP794	Octanal	Aldehyde	1025100	1045900	906920	1920500	1849300	1672000
MEDP796	Pyrene	Benzene and substituted derivatives	76025	70484	82344	86443	70588	83597
MEDP799	(-)-Menthone	Ketones	8341800	8698600	7989800	8798900	8126300	7825200
MEDP810	Linalyl oxide	Alcohol	42613	37286	42965	57760	56367	60206
MEDP817	PROPYL HEXANOATE	Fatty acyls	3465100	3198600	3154300	3497100	3857800	3775100
MEDP818	1-PENTADECANOL	Alcohol	2694300	2717200	2698400	2877400	2726600	2602300
MEDP821	6-Methylnicotinamide	Pyridine And Pyridine Derivatives	824940	763580	697390	198280	238320	296730
MEDP824	Trimethoprim	Benzene and substituted derivatives	38564	24241	30009	23982	34071	9
MEDP827	ISOPHORONE	Ketones	15003	5915.2	5660.8	35944	18774	20509
MEDP831	1-Aminopropan-2-ol	Alcohol	2307600	2542100	2313200	11172000	9218600	7236100
MEDP834	3-Methylsalicylic acid	Organic Acid And Its Derivatives	147040	176060	132110	145120	171310	124170
MEDP836	Dibutyl phthalate	Benzene and substituted derivatives	27987000	27382000	26700000	29449000	30004000	30015000
MEDP839	Pulegone	Ketones	148380	161780	115420	136400	133020	133980
MEDP844	FURFURYL ALCOHOL	Alcohol	825670	858220	759710	1033100	754200	753970
MEDP845	1-Phenylethanol	Benzene and substituted derivatives	274280000	283510000	280930000	269010000	253840000	238460000
MEDP849	Nα-Acetyl-L-glutamine	Amino Acid metabolomics	2974900	3114000	3486400	6874000	7202400	7903100
MEDP853	Choline chloride	Others	76255000	75397000	75593000	50300000	48546000	44610000
MEDP859	glycylphenylalanine	Amino Acid metabolomics	3157900	3091100	3261100	1422500	1532500	1590200
MEDP874	tryptophan betaine	Organic Acid And Its Derivatives	1249500	1185300	1321100	1616300	1620600	1458200

MEDP876	O-Succinyl-L-Homoserine	Amino Acid	metabolomics	190950	149710	131290	238180	325710	272130
MEDP878	N-Methyl-L-Glutamate	Amino Acid	metabolomics	2906200	2978100	2747300	2782200	2473300	2183100
MEDP879	N-Methyl-D-Aspartic Acid	Amino Acid	metabolomics	6475900	6705800	5352100	2564900	2608800	2804200
MEDP880	N-Alpha-Acetyl-L-Asparagine	Amino Acid	metabolomics	9	9	9	1539900	1440100	1401700
MEDP881	Phosphocholine	Nucleotide metabolomics		4454700	3517900	3146000	3110700	3540500	3824300
MEDP883	2,4-Dihydroxypteridine	Pteridines and derivatives		366020	309030	310380	262220	333380	281150
MEDP884	L-Methionine Sulfoximine	Organic Acid And Derivatives	Its	99174	72073	135460	98893	108500	104110
MEDP885	Mesoxalate	Organic Acid And Derivatives	Its	7976200	9119900	9292900	8565700	7553300	6969500
MEDP886	N-Amidino-L-Aspartate	Organic Acid And Derivatives	Its	1097500	1159000	1078400	1443900	1623400	1433300
MEDP890	Lumichrome	Others		42110	34750	34680	17064	19021	17211
MEDP895	Dethiobiotin	Others		564040	578340	588800	93743	133340	147180
MEDP896	2',4'-Dihydroxyacetophenone	Benzene and substituted derivatives		2223900	2661700	3064400	5019500	5992900	4715900
MEDP899	3-Methyladenine	Nucleotide metabolomics		30217	22590	24453	9	9	5654.2
MEDP904	Oleate	Lipids		47607	54041	56977	41108	44240	37278

File S5. OPLS-DA analysis of differential metabolites in broiler chickens with TD.

Index	Compounds	Class	VIP	Fold_Change	Log2FC	Type
MEDP514	Thiamine	CoOthersEnzyme Factor & vitamin	1.191676496	361366.6667	18.46310391	up
MEDP880	N-Alpha-Acetyl-L-Asparagine	Amino Acid metabolomics	1.192016855	162285.1852	17.30817178	up
MEDN704	Oxaloacetic acid	Organic Acid And Its Derivatives	1.191925982	71369.62963	16.12302267	up
MEDP275	Indole-3-Acetic Acid	Indole And Its Derivatives	1.192017671	40507.77778	15.30591132	up
MEDP546	Oxindole	Indole And Its Derivatives	1.19199921	39921.11111	15.28486425	up
MEDN161	Guanosine 3',5'-Cyclic Monophosphate	Nucleotide metabolomics	1.192021573	23247.40741	14.50478221	up
MEDP317	Hydrocinnamic Acid	Organic Acid And Its Derivatives	1.191890213	19766.66667	14.27078198	up
MEDP156	5-Methyluridine	Nucleotide metabolomics	1.191931573	14625.92593	13.83624034	up
MEDP371	Acetaminophen Glucuronide	Carbohydrate metabolomics	1.191641427	9185.666667	13.16516872	up
MEDP396	2'-Deoxycytidine-5'-Monophosphate	Nucleotide metabolomics	1.191332024	4953.333333	12.27418399	up
MEDP244	All-Trans-13,14-Dihydroretinol	CoOthersEnzyme Factor & vitamin	1.190975662	4946.851852	12.27229498	up
MEDP517	Palmitoylcarnitine	Camitine	1.19194365	3671.666667	11.84221937	up
MEDP072	N-Isovaleroylglycine	Amino Acid metabolomics	1.189412933	2349.555556	11.19817217	up
MEDP385	L-Sepiapterin	Pteridines and derivatives	1.190062723	1758.814815	10.78038787	up
MEDP086	Urea	Amino Acid metabolomics	1.191487469	199.4709138	7.640034582	up
MEDP274	Indole-2-Carboxylic Acid	Indole And Its Derivatives	1.007667737	69.09087591	6.110423297	up
MEDP374	Cyclic Amp	Nucleotide metabolomics	1.190773767	68.8389144	6.105152441	up
MEDP169	Guanosine Monophosphate	Nucleotide metabolomics	1.173983916	32.2541483	5.011412816	up
MEDP361	4-Hydroxybenzaldehyde	Benzene and substituted derivatives	1.186747811	30.351302	4.923686501	up
MEDN555	Hydroxyphenyllactic acid	Carbohydrate metabolomics	1.190792546	18.35285957	4.197932962	up
MEDP602	1-(4-Methoxyphenyl)-2-propanone	Benzene and substituted derivatives	1.18992437	12.35568455	3.627103038	up

MEDP180	B-Nicotinamide Mononucleotide	Nucleotide metabolomics	1.139995363	8.017121538	3.003084345	up
MEDP442	Sn-Glycero-3-Phosphocholine	Cholines	1.174269461	7.23328812	2.854651619	up
MEDN523	Indolelactic acid	Indole And Its Derivatives	1.19144213	6.833414175	2.772606572	up
MEDN340	Pyrrole-2-Carboxylic Acid	Organic Acid And Its Derivatives	1.141366151	6.571608065	2.71624644	up
MEDP218	D-Mannitol	Carbohydrate metabolomics	1.19035819	6.218696381	2.636612181	up
MEDN338	Phenyllactate (Pla)	Organic Acid And Its Derivatives	1.191074948	6.207425706	2.633995089	up
MEDP378	N6-Succinyl Adenosine	Nucleotide metabolomics	1.132186851	5.017976501	2.327105715	up
MEDP319	Kynurenic Acid	Organic Acid And Its Derivatives	1.144988168	4.940713337	2.304719352	up
MEDN615	Carbamoyl phosphate	Organic Acid And Its Derivatives	1.156721067	4.92153709	2.299108967	up
MEDP316	Homovanillic Acid	Organic Acid And Its Derivatives	1.186413266	4.627476429	2.210225641	up
MEDP461	2-Methylguanosine	Nucleotide metabolomics	1.095517482	4.623520273	2.208991715	up
MEDP380	2-(Dimethylamino)Guanosine	Nucleotide metabolomics	1.07511738	4.411221129	2.141178083	up
MEDN166	Nicotinic Acid Adenine Dinucleotide	Nucleotide metabolomics	1.008310033	4.345176609	2.119414816	up
MEDP540	7,8-dihydro-L-Biopterin	Pteridines and derivatives	1.17068139	4.151239442	2.053542148	up
MEDP084	Trimethylamine N-Oxide	Amino Acid metabolomics	1.165962238	3.984560938	1.994420761	up
MEDN213	D-Sorbitol	Carbohydrate metabolomics	1.180243331	3.937804784	1.977391592	up
MEDP350	Lysopc 20:1	LipidsOthersPhospholipid	1.187957386	3.89789801	1.962696342	up
MEDP831	1-Aminopropan-2-ol	Alcohol	1.169551395	3.856915495	1.947447537	up
MEDN621	Indoxylsulfuric acid	Organic Acid And Its Derivatives	1.188079896	3.851660109	1.945480398	up
MEDN120	Dulcitol	Carbohydrate metabolomics	1.179084791	3.524003892	1.817215518	up
MEDP588	Ritalinic acid	Organic Acid And Its Derivatives	1.076489127	3.51863502	1.815015875	up
MEDP170	Hypoxanthine	Nucleotide metabolomics	1.073127603	3.511802029	1.812211518	up
MEDP034	3-N-Methyl-L-Histidine	Amino Acid metabolomics	1.184466874	3.461908619	1.791567644	up
MEDP046	Hexanoyl Glycine	Amino Acid metabolomics	1.180878925	3.457870594	1.789883879	up
MEDP379	3-Hydroxyhippuric Acid	Organic Acid And Its Derivatives	1.166554458	3.323982405	1.732912745	up
MEDN579	N-lactoyl-phenylalanine	Organic Acid And Its Derivatives	1.185229679	3.275492117	1.711711676	up

MEDP073	N-Phenylacetylglycine	Amino Acid metabolomics	1.178746042	3.259497941	1.704649764	up
MEDP066	N-Acetyl-L-Tyrosine	Amino Acid metabolomics	1.185151319	3.166144201	1.662726964	up
MEDP149	2'-Deoxyadenosine-5'-Monophosphate	Nucleotide metabolomics	1.180551886	3.101934328	1.633168143	up
MEDP550	18-Hydroxycorticosterone	Lipids	1.151707145	3.089956919	1.627586724	up
MEDN471	3-Methoxy-4-Hydroxyphenylethyleneglycol Sulfate	Organic Acid And Its Derivatives	1.188339701	2.9775256	1.574113912	up
MEDP718	Methylisobutyrate	Organic Acid And Its Derivatives	1.172259196	2.960608122	1.565893542	up
MEDP393	Pyridoxine 5'-Phosphate	CoOthersEnzyme Factor & vitamin	1.116148301	2.957289511	1.564275486	up
MEDP386	N-Acetylthreonine	Amino Acid metabolomics	1.191204115	2.887061833	1.529602007	up
MEDN093	3-Hydroxyanthranilic Acid	Benzoic Acid And Its Derivatives	1.181699556	2.857306777	1.514655941	up
MEDN528	(3-Methoxy-4-hydroxyphenyl)ethylene glycol sulfate	Organic Acid And Its Derivatives	1.187144544	2.841602948	1.506704983	up
MEDP352	Lysopc 20:2	LipidsOthersPhospholipid	1.189964123	2.820111652	1.495752282	up
MEDN485	D-Fructose-1,6-Biphosphate-Trisodium Salt	Carbohydrate metabolomics	1.096970781	2.758869807	1.464077376	up
MEDN210	1,5-Anhydro-D-Glucitol	Carbohydrate metabolomics	1.185158943	2.753902924	1.461477705	up
MEDP434	Lysopc 17:0	LipidsOthersPhospholipid	1.179879051	2.747897288	1.45832808	up
MEDP384	Isoxanthopterin	Pteridines and derivatives	1.16481198	2.650265163	1.40613671	up
MEDP069	N-Acetylputrescine	Organic Acid And Its Derivatives	1.182766213	2.62690765	1.393365483	up
MEDN324	L-3-Phenyllactic Acid	Organic Acid And Its Derivatives	1.188383042	2.618831654	1.388923322	up
MEDN502	2-Deoxyribose 1-Phosphate	Carbohydrate metabolomics	1.113222762	2.612000403	1.385155119	up
MEDN649	Anthranilic acid	Benzene and substituted derivatives	1.187749494	2.602179837	1.37972067	up
MEDP238	Vitamin D3	CoOthersEnzyme Factor & vitamin	1.143374883	2.601663624	1.379434444	up
MEDP638	LysoPE(16:1(9Z)/0:0)	Lipids_Fatty Acids	1.028316566	2.578989036	1.36680564	up
MEDN284	2-Hydroxyisocaproic Acid	Organic Acid And Its Derivatives	1.191267413	2.573480938	1.363721096	up

MEDP363	Biopterin	Pteridines and derivatives	1.138373123	2.571752418	1.362751761	up
MEDP642	3-Hydroxy-DL-kynurenine	Amino Acid metabolomics	1.176806437	2.508805517	1.327000637	up
MEDP739	Hexyl??Acetate	Fatty acyls	1.187419095	2.503360215	1.323865899	up
MEDP111	3-(4-Hydroxyphenyl)-Propionic Acid	Benzene and substituted derivatives	1.188239246	2.482777215	1.311954811	up
MEDP346	Lysopc 18:2	LipidsOthersPhospholipid	1.037675045	2.448920119	1.292145716	up
MEDN366	Lysope 16:0	LipidsOthersPhospholipid	1.163700191	2.433449527	1.283002851	up
MEDP012	L-Ornithine	Amino Acid metabolomics	1.172284939	2.41083662	1.269533884	up
MEDP437	Ergothioneine	Organic Acid And Its Derivatives	1.182787334	2.409680257	1.268841727	up
MEDN032	Allantoin	Organic Acid And Its Derivatives	1.152332505	2.401309315	1.26382125	up
MEDP455	Dihydrojasmone	Organic Acid And Its Derivatives	1.180728374	2.38984441	1.256916695	up
MEDP450	P-Aminobenzoate	Benzene and substituted derivatives	1.136377317	2.371778967	1.245969567	up
MEDP035	5-Hydroxy-L-Tryptophan	Amino Acid metabolomics	1.130004717	2.35394563	1.235080998	up
MEDP618	2-Methylbutyroylcarnitine	Lipids_Fatty Acids	1.12940272	2.3355138	1.22373997	up
MEDP062	N6-Acetyl-L-Lysine	Amino Acid metabolomics	1.177594168	2.310793834	1.20838855	up
MEDP342	Lysopc 18:0	LipidsOthersPhospholipid	1.180893593	2.307167714	1.206122881	up
MEDP849	Nα-Acetyl-L-glutamine	Amino Acid metabolomics	1.179386695	2.295437219	1.198768974	up
MEDP537	PAF C-16	Lipids	1.179543692	2.286735654	1.1932896	up
MEDN651	Succinic anhydride	Organic Acid And Its Derivatives	1.180595455	2.274940746	1.185828969	up
MEDN206	Citramalic Acid	Amino Acid metabolomics	1.176296831	2.261667319	1.177386731	up
MEDP584	Pterine	Pteridines and derivatives	1.181998454	2.2593288	1.175894242	up
MEDP529	Indole-3-acetamide	Indole And Its Derivatives	1.18522706	2.248150211	1.168738433	up
MEDP147	1-Methylhistidine	Amino Acid metabolomics	1.18958861	2.247399527	1.168256619	up
MEDN343	Shikimic Acid	Organic Acid And Its Derivatives	1.18511148	2.242811757	1.165308538	up
MEDP059	L-Theanine	Amino Acid metabolomics	1.15992666	2.209146436	1.143489053	up

MEDP145	1,7-Dimethylxanthine	Nucleotide metabolomics	1.142647656	2.205951393	1.141401002	up
MEDP251	Trigonelline	CoOthersEnzyme Factor & vitamin	1.179052181	2.199188871	1.136971511	up
MEDP336	Lysopc 14:0	LipidsOthersPhospholipid	1.18358954	2.183882167	1.126895017	up
MEDN200	L-Malic Acid	Amino Acid metabolomics	1.157055121	2.173385548	1.119944124	up
MEDP295	4-Acetamidobutyric Acid	Organic Acid And Its Derivatives	1.187638257	2.151596793	1.105407744	up
MEDN364	Lysope 18:0	LipidsOthersPhospholipid	1.134312456	2.129844083	1.090747821	up
MEDP587	N-Alpha-acetyllysine	Organic Acid And Its Derivatives	1.189506852	2.096432831	1.067936608	up
MEDP247	Nicotinuric Acid	CoOthersEnzyme Factor & vitamin	1.101082702	2.094585231	1.06666459	up
MEDN679	Maltol	Heterocyclic compound	1.117980416	2.09178619	1.064735396	up
MEDP344	Lysopc 18:1	LipidsOthersPhospholipid	1.144451259	2.088948365	1.062776832	up
MEDP719	N-Nitrosodiethylamine	Polyamine	1.181818693	2.084627192	1.0597894	up
MEDN128	Vanillin	Phenols And Its Derivatives	1.174261689	2.083194082	1.058797255	up
MEDP498	Lysopc 18:3	LipidsOthersPhospholipid	1.128554236	2.057232944	1.040705162	up
MEDN587	N-Acetylvaline	Organic Acid And Its Derivatives	1.005599614	2.054763781	1.038972549	up
MEDN720	N-(2-Methylbenzoyl)glycine	Amino Acid metabolomics	1.153630259	2.040631806	1.029015898	up
MEDP401	5'-Deoxy-5'-(Methylthio) Adenosine	Nucleotide metabolomics	1.175768908	2.024298019	1.017421701	up
MEDN376	9,10-DiHOME [(±)9,10-dihydroxy-12Z-octadecenoic acid]	Oxidized lipid	1.159970065	0.489638391	-1.030211416	down
MEDN481	2,4-Dihydroxybenzoic Acid	Benzene and substituted derivatives	1.186116821	0.480732246	-1.056694517	down
MEDP053	L-Dopa	Amino Acid metabolomics	1.072507589	0.479870732	-1.059282271	down
MEDP859	glycylphenylalanine	Amino Acid metabolomics	1.185994286	0.477933986	-1.065116733	down
MEDP890	Lumichrome	Others	1.169134937	0.477819616	-1.065462012	down
MEDP382	8-Hydroxy-2-Deoxyguanosine	Nucleotide metabolomics	1.09331965	0.477503867	-1.066415677	down
MEDP171	Inosine	Nucleotide metabolomics	1.166092147	0.474198053	-1.076438356	down

MEDP454	Isoquinoline	Benzene and substituted derivatives	1.170198389	0.472647683	-1.081162914	down
MEDN105	Taurocholic Acid	Bile Acids	1.147728252	0.469403215	-1.091100372	down
MEDN569	DL-o-Tyrosine	Organic Acid And Its Derivatives	1.107001857	0.469070137	-1.092124438	down
MEDP054	L-Glutamine	Amino Acid metabolomics	1.171312504	0.463484397	-1.109407323	down
MEDP539	Ubiquinone-1	Lipids_Fatty Acids	1.191882247	0.463433143	-1.10956687	down
MEDP594	Dihydroactinidiolide	Benzene and substituted derivatives	1.177705059	0.458571542	-1.124781268	down
MEDP241	Orotic Acid	CoOthersEnzyme Factor & vitamin	1.156621255	0.452867472	-1.142839174	down
MEDP425	Imidazoleacetic acid	Indole And Its Derivatives	1.190674988	0.448143468	-1.157967427	down
MEDP407	Vitamin A	CoOthersEnzyme Factor & vitamin	1.111886566	0.441064914	-1.180937093	down
MEDP375	Deoxyadenosine	Nucleotide metabolomics	1.047670891	0.438509972	-1.189318443	down
MEDP879	N-Methyl-D-Aspartic Acid	Amino Acid metabolomics	1.174122754	0.430451392	-1.216077764	down
MEDP551	N-METHYL (-)EPHEDRINE	Benzene and substituted derivatives	1.16788138	0.430233646	-1.216807741	down
MEDN010	L-Citrulline	Amino Acid metabolomics	1.189745603	0.417419064	-1.260431605	down
MEDP291	3,7-Dimethyluric Acid	Organic Acid And Its Derivatives	1.071654198	0.413336416	-1.27461162	down
MEDP334	Vanillic Acid	Organic Acid And Its Derivatives	1.18711772	0.396733135	-1.333759199	down
MEDP160	Adenosine	Nucleotide metabolomics	1.150913564	0.395630282	-1.33777524	down
MEDP369	4-(Aminomethyl)-5-(Hydroxymethyl)-2-Methylpyridin-3-Ol	Pyridine And Pyridine Derivatives	1.167437783	0.392415242	-1.349547018	down
MEDN115	Chenodeoxycholic Acid	Bile Acids	1.09054708	0.389718558	-1.359495463	down
MEDN407	3-Aminosalicylic Acid	Organic Acid And Its Derivatives	1.132755088	0.388675229	-1.363362931	down
MEDP212	N-Acetyl-5-Hydroxytryptamine	Tryptamines And Its Derivatives	1.0784453	0.373317844	-1.421523626	down
MEDN013	L-Isoleucine	Amino Acid metabolomics	1.184132117	0.372463066	-1.424830724	down
MEDP167	Guanosine	Nucleotide metabolomics	1.182660482	0.364935674	-1.454285907	down

MEDP150	2'-Deoxyinosine	Nucleotide metabolomics	1.055431443	0.360918438	-1.470255247	down
MEDP143	Biotin	CoOthersEnzyme Factor & vitamin	1.187618495	0.36060027	-1.471527616	down
MEDP250	Riboflavin	CoOthersEnzyme Factor & vitamin	1.190243612	0.356459877	-1.4881884	down
MEDN594	m-Coumaric acid	Benzene and substituted derivatives	1.1772073	0.356044477	-1.48987062	down
MEDN025	3-Hydroxy-3-Methylpentane-1,5-Dioic Acid	Amino Acid metabolomics	1.179323672	0.346626323	-1.528546877	down
MEDN045	L-Cysteine	Amino Acid metabolomics	1.167281041	0.345387195	-1.533713499	down
MEDP020	L-Methionine	Amino Acid metabolomics	1.173404659	0.323248947	-1.629282426	down
MEDN339	Phenylpyruvic Acid	Organic Acid And Its Derivatives	1.158652627	0.323149749	-1.629725225	down
MEDP821	6-Methylnicotinamide	Pyridine And Pyridine Derivatives	1.164103617	0.320804406	-1.640234138	down
MEDP056	L-Homocystine	Amino Acid metabolomics	1.170780654	0.302698649	-1.724045858	down
MEDP011	L-Lysine	Amino Acid metabolomics	1.16979012	0.300857617	-1.732847211	down
MEDN748	Methylparaben	Benzoic Acid And Its Derivatives	1.145425162	0.297077305	-1.751089697	down
MEDN159	Flavin Adenine Dinucleotide	Nucleotide metabolomics	1.142128295	0.287956485	-1.79607728	down
MEDN593	D-(+)-Malic acid	Amino Acid metabolomics	1.18856469	0.276959842	-1.85225129	down
MEDP590	p-Mentha-1,3,8-triene	Lipids_Fatty Acids	1.106492281	0.2764283	-1.855022772	down
MEDN042	L-Asparagine Anhydrous	Amino Acid metabolomics	1.179523436	0.275893344	-1.857817442	down
MEDP297	5-Aminovaleric Acid	Organic Acid And Its Derivatives	1.189238888	0.27531507	-1.860844512	down
MEDP313	Guanidineacetic Acid	Organic Acid And Its Derivatives	1.188440285	0.26618705	-1.909487707	down
MEDP163	Cytidine	Nucleotide metabolomics	1.147801691	0.260313277	-1.941679196	down
MEDN533	Xanthosine	Nucleotide metabolomics	1.107931027	0.259313864	-1.947228752	down
MEDP178	Uracil	Nucleotide metabolomics	1.131897829	0.258663222	-1.950853156	down
MEDP071	N-Glycyl-L-Leucine	Amino Acid metabolomics	1.175520283	0.258348508	-1.952609542	down
MEDP356	Methyl Nicotinate	CoOthersEnzyme Factor & vitamin	1.171588608	0.256331477	-1.963917444	down
MEDP106	Benzoic Acid	Benzene and substituted	1.157573104	0.254871886	-1.972155854	down

		derivatives				
MEDN124	Protocatechuic Acid	Phenols And Its Derivatives	1.189798373	0.254685299	-1.973212408	down
MEDN280	2-Aminoethanesulfonic Acid	Organic Acid And Its Derivatives	1.179256295	0.243360547	-2.038832795	down
MEDP282	1,3-Dimethyluric Acid	Organic Acid And Its Derivatives	1.156773412	0.241535333	-2.049693846	down
MEDP010	L-Arginine	Amino Acid metabolomics	1.18799331	0.23797212	-2.071135532	down
MEDP214	Tryptamine	Tryptamines And Its Derivatives	1.180338746	0.236575991	-2.079624427	down
MEDP148	1-Methylxanthine	Nucleotide metabolomics	1.181537911	0.232391979	-2.105367817	down
MEDP895	Dethiobiotin	Others	1.173678951	0.216189535	-2.209631403	down
MEDP089	N-Acetylhistamine	Organic Acid And Its Derivatives	1.189381676	0.215643385	-2.213280637	down
MEDP637	L-phenylalanyl-L-proline	Organic Acid And Its Derivatives	1.182919213	0.197654956	-2.338943962	down
MEDP302	Caffeic Acid	Organic Acid And Its Derivatives	1.171992219	0.189547229	-2.399370733	down
MEDP680	Hexylamine	Amines	1.188192172	0.185570408	-2.429961424	down
MEDN506	N-Acetylglucosamine 1-Phosphate	Carbohydrate metabolomics	1.182115087	0.178889555	-2.482858944	down
MEDP441	N-Caffeoyl Putrescine	Phenolamides	1.187857565	0.165560551	-2.594569137	down
MEDP665	Methyl isobutyl ketone	Ketones	1.190443262	0.15146863	-2.722909057	down
MEDP403	Deoxycytidine	Nucleotide metabolomics	1.188841474	0.150253844	-2.734526199	down
MEDP232	N-Acetyl-D-Glucosamine	Carbohydrate metabolomics	1.188874087	0.144256732	-2.79328945	down
MEDP246	Nicotinic Acid	CoOthersEnzyme Factor & vitamin	1.179955204	0.137522276	-2.862262771	down
MEDN406	3-(3-Hydroxyphenyl)Propionate Acid	Organic Acid And Its Derivatives	1.189502235	0.113173042	-3.143397743	down
MEDP166	Deoxyguanosine	Nucleotide metabolomics	1.159114263	0.091092009	-3.456531692	down
MEDP271	3-Indolepropionic Acid	Indole And Its Derivatives	1.176404847	0.071421569	-3.807496354	down
MEDP067	N-Acetylmannosamine	Amino Acid metabolomics	1.187835765	0.071122124	-3.813557782	down
MEDN098	2-Picolinic Acid	Pyridine And Pyridine Derivatives	1.183053396	0.058602346	-4.092897778	down
MEDP115	1,4-Dihydro-1-Methyl-4-Oxo-3-Pyridine carboxamide	Pyridine And Pyridine Derivatives	1.179576718	0.040631186	-4.621268697	down
MEDP308	Dodecanedioic Aicd	Organic Acid And Its Derivatives	1.188727059	0.032906165	-4.92549827	down

MEDN140	2,6-Dihydroxypurine	Nucleotide metabolomics	1.174360092	0.021739384	-5.523545124	down
MEDN647	Hydroquinone	Phenols And Its Derivatives	1.192052604	2.33701E-05	-15.38497357	down
MEDN403	Urocanic Acid	Organic Acid And Its Derivatives	1.191911347	1.10019E-05	-16.47188686	down
